# Supplementary material for: Inoculation and colonization of the entomopathogenic fungi, Isaria javanica and Purpureocillium lilacinum, in tomato plants, and their effect on seedling growth, mortality and adult emergence of Bemisia tabaci (Gennadius)
Source: PLoS One. 2023 May 22;18(5):e0285666. doi: 10.1371/journal.pone.0285666 (PMC10202273; doi:10.1371/journal.pone.0285666)
Supplement: S1 Fig — (A) Cage used to examine the effect of endophytic EPF on B. tabaci Population (B) Cage used to examine Adult Emergence of B. tabaci. (DOCX) [file pone.0285666.s001.docx]

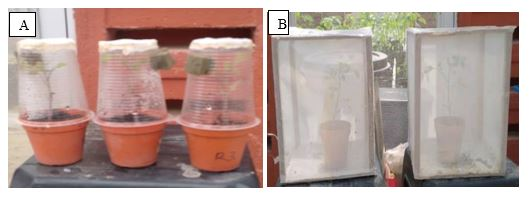


**S1 Fig. Bioassay cages used to evaluate the effect of endophytic EPF against *B. tabaci*.** (A) Cage used to examine effect of endophytic EPF on *B. tabaci* Population (B) Cage used to examine Adult Emergence of *B. tabaci*
