# Supplementary material for: Inoculation and colonization of the entomopathogenic fungi, Isaria javanica and Purpureocillium lilacinum, in tomato plants, and their effect on seedling growth, mortality and adult emergence of Bemisia tabaci (Gennadius)
Source: PLoS One. 2023 May 22;18(5):e0285666. doi: 10.1371/journal.pone.0285666 (PMC10202273; doi:10.1371/journal.pone.0285666)
Supplement: S2 Fig — (A) Leaf, (B) stem, and (C) root tissues following seed treatment with a fungal conidia suspensions. (DOCX) [file pone.0285666.s002.docx]

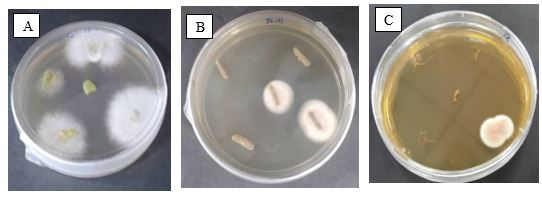


**S2 Fig. Examples of endophytic EPF (Cjc-03 and TS-01) re-isolation from the tomato plant tissues.** (A) Leaf, (B) stem, and (C) root tissues following seed treatment with fungal conidia suspension
