# Supplementary material for: Inoculation and colonization of the entomopathogenic fungi, Isaria javanica and Purpureocillium lilacinum, in tomato plants, and their effect on seedling growth, mortality and adult emergence of Bemisia tabaci (Gennadius)
Source: PLoS One. 2023 May 22;18(5):e0285666. doi: 10.1371/journal.pone.0285666 (PMC10202273; doi:10.1371/journal.pone.0285666)
Supplement: S3 Fig — (A) Cjc-03 (I. javanica) (B) TS-01 (P. lilacinum) with the noticeable spores indicated that the spores are alive on the plant tissues; Germination of conidia and appressorium formation in the stem tissues of (C) Cjc-03 (I. javanica), (D) TS-01 (P. lilacinum). (DOCX) [file pone.0285666.s003.docx]

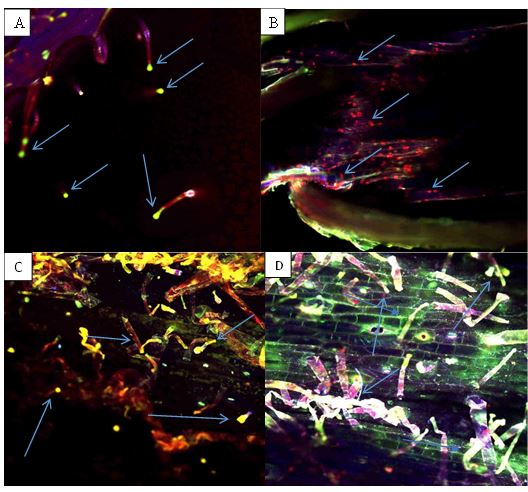


S3 Fig. Representativelaser scanning confocal microscopy images of 30 days old tomato plant tissues colonized by endophytic EPF. (A) Cjc-03 (*I. javanica*) (B) TS-01 (*P. lilacinum*) with the noticeable spores indicated that the spores are alive on the plant tissues; Germination of conidia and appressorium formation on the stem tissues of (C) Cjc-03 (*I. javanica*), (D) TS-01 (*P. lilacinum*).
