# Supplementary material for: Inoculation and colonization of the entomopathogenic fungi, Isaria javanica and Purpureocillium lilacinum, in tomato plants, and their effect on seedling growth, mortality and adult emergence of Bemisia tabaci (Gennadius)
Source: PLoS One. 2023 May 22;18(5):e0285666. doi: 10.1371/journal.pone.0285666 (PMC10202273; doi:10.1371/journal.pone.0285666)
Supplement: S1 Table — Species, accession number, origin, and EPF host species. (DOCX) [file pone.0285666.s004.docx]

**S1 Table. Fungal endophytes used in the study.** Species, accession number, origin, and host of EPF species

| **Isolates code** | **Species** | **GenBank Accession** | **Origin** | **Host** |
| --- | --- | --- | --- | --- |
| CjC-03 | *I. javanica* | MW857170 | UPM Serdang, Selangor Malaysia | *B. tabaci* |
| TS-01 | *P. lilacinum* | MW857173 | UPM Serdang, Selangor Malaysia | *B. tabaci* |
