# Supplementary material for: Inoculation and colonization of the entomopathogenic fungi, Isaria javanica and Purpureocillium lilacinum, in tomato plants, and their effect on seedling growth, mortality and adult emergence of Bemisia tabaci (Gennadius)
Source: PLoS One. 2023 May 22;18(5):e0285666. doi: 10.1371/journal.pone.0285666 (PMC10202273; doi:10.1371/journal.pone.0285666)
Supplement: Attachment — Submitted filename: Supporting information _Plos one.pdf [file pone.0285666.s006.pdf]

Supporting information

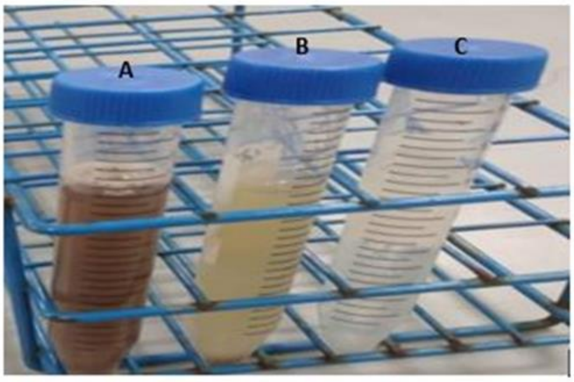

S1 Fig. Treatments used to inoculate tomato seeds. (A). *P. lilacinum* (TS-01); (B). *C. javanica* (Cjc-03); (C) 0.01% Tween 80 solution as control.

Comentado [R1]: Not interesting or useful

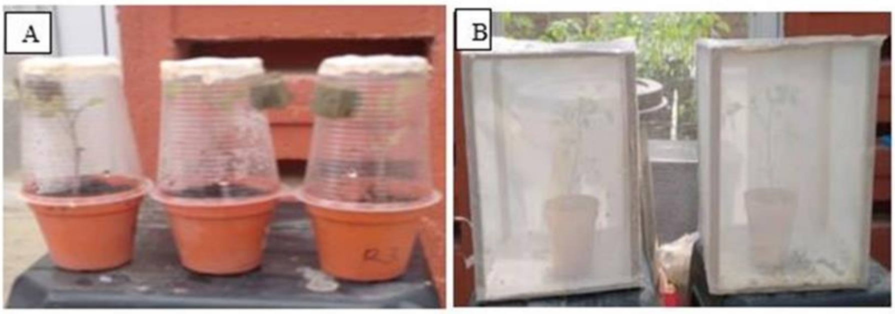

S2 Fig. Bioassay cages used to evaluate the effect of endophytic EPF against *B. tabaci*. (A) cage used to examine effect of endophytic EPF on *B. tabaci* Population (B) Cage used to examine Adult Emergence of *B. tabaci*

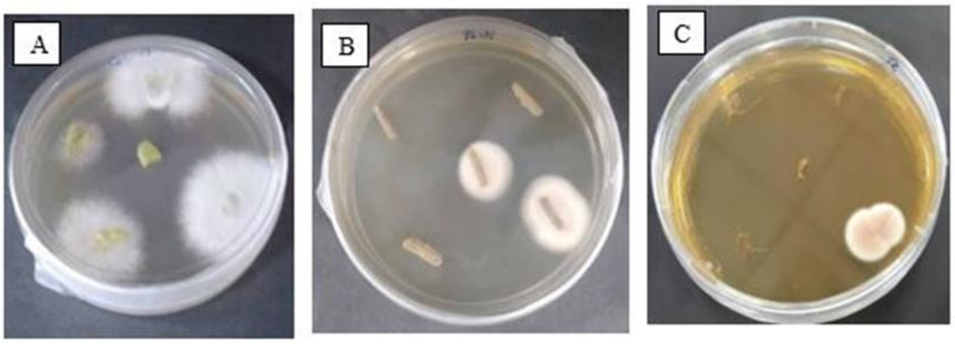

S3 Fig. Examples of endophytic EPF (Cjc-03 and TS-01) re-isolation from the tomato plant tissues. (A) Leaf, (B) stem, and (C) root tissues following seed treatment with fungal conidia suspension

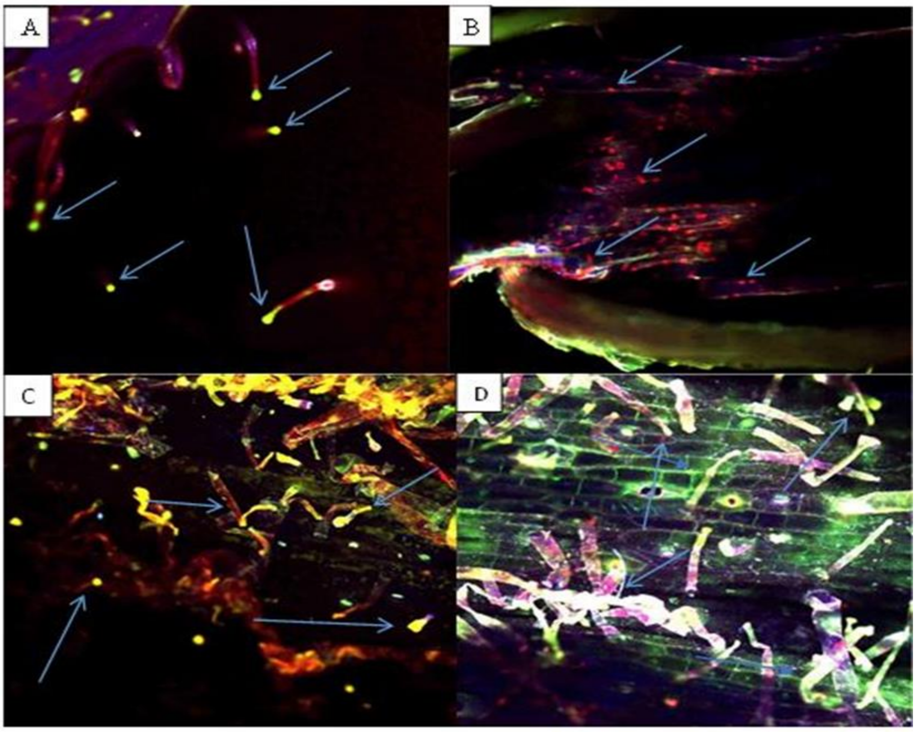

S4 Fig. Representative laser scanning confocal microscopy images of 30 days old tomato plant tissues colonized by endophytic EPF. (A) Cjc-03 (*C. javanica*) (B) TS-01 (*P.

lilacinum) with the noticeable spores indicated that the spores are alive on the plant tissues; Germination of conidia and appressorium formation on the stem tissues of (C) Cjc-03 (*C. javanica*), (D) TS-01 (*P. lilacinum*).

S1 Table. Fungal endophytes used in the study. Species, accession number, origin, and host of EPF species

Isolates code	Species	GenBank Accession	Origin	Host
CjC-03	*C. javanica*	MW857170	UPM Serdang, Selangor Malaysia	*B. tabaci*
TS-01	*P. lilacinum*	MW857173	UPM Serdang, Selangor Malaysia	*B. tabaci*
