# Supplementary material for: Inoculation and colonization of the entomopathogenic fungi, Isaria javanica and Purpureocillium lilacinum, in tomato plants, and their effect on seedling growth, mortality and adult emergence of Bemisia tabaci (Gennadius)
Source: PLoS One. 2023 May 22;18(5):e0285666. doi: 10.1371/journal.pone.0285666 (PMC10202273; doi:10.1371/journal.pone.0285666)
Supplement: Attachment — Submitted filename: PONE-D-22-19772_sks.pdf [file pone.0285666.s007.pdf]

PLOS ONE

Inoculation and colonization of entomopathogenic fungi, Cordyceps javanica and Purpureocillium lilacinum in tomato plants, and their effect on seedlings growth, population, and adult emergence of whitefly, B. tabaci (Gennadius).

--Manuscript Draft--

**Manuscript Number:**	PONE-D-22-19772
**Article Type:**	Research Article
**Full Title:**	Inoculation and colonization of entomopathogenic fungi, Cordyceps javanica and Purpureocillium lilacinum in tomato plants, and their effect on seedlings growth, population, and adult emergence of whitefly, B. tabaci (Gennadius).
**Short Title:**	Endophytic entopathogenic fungi Promote growth of tomato and negatively affected Whitefly, B. tabaci
**Corresponding Author:**	Syari Jamian, Ph.D<br>UPM - Serdang: Universiti Putra Malaysia<br>Serdang, Selangor MALAYSIA
**Keywords:**	colonization; biocontrol, endophytic; Bemisia tabaci; Cordyceps javanica; and Purpureocillium lilacinum
**Abstract:**	Entomopathogenic fungi (EPF) are natural enemies present in the insect population and have long been recognized as biological control agents against many insect pests. Some of the isolates have also been established as endophytes, benefiting their host plants without causing any symptoms or negative effects. Here we experimented to demonstrate two isolated fungal species of Cordyceps javanica and Purpureocillium lilacinum as an endophyte in the tomato plant by using the seed inoculation method and examining their effect on plant growth, population, and adult emergence of B. tabaci. Our study indicated that tomato seeds treated with a fungal suspension of C. javanica and P. lilacinum allowed their recovery up to 60 days after inoculation (DAI). Both endophytic isolates also reduced the significant population of adult B. tabaci in seedlings inoculated with, C. javanica (51.92±4.78%) , and P. lilacinum (45.32±0.20%) compared to the control treatment (19.29±2.35). However, the adult emergence means number was significantly high in the control treatments (57.50±2.66) compared to C. javanica (15.00±1.47) and P. lilacinum (28.75±4.78) treatments. This study provides the first report on the endophytic colonization of plants by fungal pathogens and their effects on the host plants and insect herbivores in Malaysia and some neighboring countries.
**Order of Authors:**	Ibrahim Sani, PhD
	Syari Jamian, Ph.D
	Norsazilawati Saad, Ph.D
	Sumaiyah Abdullah, Ph.D
	Erneeza Mohd Hata
	Johari Jalinas, Ph.D
	Siti Izera Ismail, Ph.D
**Additional Information:**	
**Question**	**Response**
**Financial Disclosure**<br><br>Enter a financial disclosure statement that describes the sources of funding for the work included in this submission. Review the submission guidelines for detailed requirements. View published research	This study was funded by the Fundamental Research Grant Scheme (FRGS) from the Ministry of Higher Education Malaysia (Grant no. 5540212; reference code FRGS/1/2019/WAB01/UPM/02/36). he funders had no role in study design, data collection and analysis, decision to publish, or preparation of the manuscript.

Powered by Editorial Manager® and ProduXion Manager® from Aries Systems Corporation

articles from *PLOS ONE* for specific examples.<br><br>This statement is required for submission and **will appear in the published article** if the submission is accepted. Please make sure it is accurate.<br><br>**Unfunded studies**<br>Enter: *The author(s) received no specific funding for this work.*<br><br>**Funded studies**<br>Enter a statement with the following details:<br>• Initials of the authors who received each award<br>• Grant numbers awarded to each author<br>• The full name of each funder<br>• URL of each funder website<br>• Did the sponsors or funders play any role in the study design, data collection and analysis, decision to publish, or preparation of the manuscript?<br>• **NO** - Include this sentence at the end of your statement: *The funders had no role in study design, data collection and analysis, decision to publish, or preparation of the manuscript.*<br>• **YES** - Specify the role(s) played.<br><br>* typeset	
**Competing Interests**<br><br>Use the instructions below to enter a competing interest statement for this submission. On behalf of all authors, disclose any competing interests that could be perceived to bias this work—acknowledging all financial support and any other relevant financial or non-financial competing interests.<br><br>This statement is required for submission and **will appear in the published article** if the submission is accepted. Please make sure it is accurate and that any funding sources listed in your Funding Information later in the submission form are also declared in your Financial Disclosure statement.	The authors have declared that no competing interests exist.

View published research articles from *PLOS ONE* for specific examples.<br><br>**NO authors have competing interests**<br><br>Enter: *The authors have declared that no competing interests exist.*<br><br>**Authors with competing interests**<br><br>Enter competing interest details beginning with this statement:<br><br>*I have read the journal's policy and the authors of this manuscript have the following competing interests: [insert competing interests here]*<br><br>* typeset	
**Ethics Statement**<br><br>Enter an ethics statement for this submission. This statement is required if the study involved:<br><br>• Human participants<br>• Human specimens or tissue<br>• Vertebrate animals or cephalopods<br>• Vertebrate embryos or tissues<br>• Field research<br><br>Write "N/A" if the submission does not require an ethics statement.<br><br>General guidance is provided below. Consult the submission guidelines for detailed instructions. **Make sure that all information entered here is included in the Methods section of the manuscript.**	N/A

**Format for specific study types**<br><br>**Human Subject Research (involving human participants and/or tissue)**<br>• Give the name of the institutional review board or ethics committee that approved the study<br>• Include the approval number and/or a statement indicating approval of this research<br>• Indicate the form of consent obtained (written/oral) or the reason that consent was not obtained (e.g. the data were analyzed anonymously)<br><br>**Animal Research (involving vertebrate animals, embryos or tissues)**<br>• Provide the name of the Institutional Animal Care and Use Committee (IACUC) or other relevant ethics board that reviewed the study protocol, and indicate whether they approved this research or granted a formal waiver of ethical approval<br>• Include an approval number if one was obtained<br>• If the study involved *non-human primates*, add *additional details* about animal welfare and steps taken to ameliorate suffering<br>• If anesthesia, euthanasia, or any kind of animal sacrifice is part of the study, include briefly which substances and/or methods were applied<br><br>**Field Research**<br><br>Include the following details if this study involves the collection of plant, animal, or other materials from a natural setting:<br>• Field permit number<br>• Name of the institution or relevant body that granted permission	
**Data Availability**<br><br>Authors are required to make all data underlying the findings described fully available, without restriction, and from the time of publication. PLOS allows rare exceptions to address legal and ethical concerns. See the PLOS Data Policy and FAQ for detailed information.	Yes - all data are fully available without restriction

A Data Availability Statement describing where the data can be found is required at submission. Your answers to this question constitute the Data Availability Statement and **will be published in the article**, if accepted.<br><br>**Important:** Stating 'data available on request from the author' is not sufficient. If your data are only available upon request, select 'No' for the first question and explain your exceptional situation in the text box.<br><br>Do the authors confirm that all data underlying the findings described in their manuscript are fully available without restriction?	
**Describe where the data may be found in full sentences. If you are copying our sample text, replace any instances of XXX with the appropriate details.**<br><br>• If the data are **held or will be held in a public repository**, include URLs, accession numbers or DOIs. If this information will only be available after acceptance, indicate this by ticking the box below. For example: *All XXX files are available from the XXX database (accession number(s) XXX, XXX.).*<br>• If the data are all contained **within the manuscript and/or Supporting Information files**, enter the following: *All relevant data are within the manuscript and its Supporting Information files.*<br>• If neither of these applies but you are able to provide **details of access elsewhere**, with or without limitations, please do so. For example:<br><br>*Data cannot be shared publicly because of [XXX]. Data are available from the XXX Institutional Data Access / Ethics Committee (contact via XXX) for researchers who meet the criteria for access to confidential data.*<br><br>*The data underlying the results presented in the study are available from (include the name of the third party*	All relevant data are within the manuscript and its Supporting Information files.

and contact information or URL).<br>• This text is appropriate if the data are owned by a third party and authors do not have permission to share the data.<br><br>* typeset	
Additional data availability information:	

Manuscript Click here to access/download;Manuscript;Plos one_Article.docx

Inoculation and Colonization of Entomopathogenic Fungi, *Cordyceps javanica* and *Purpureocillium lilacinum* in Tomato Plants, and their effect on Seedlings Growth, Population, and adult Emergence of Whitefly, *Bemisia tabaci* (Gennadius)

Ibrahim Sani[1,2], Syari Jamian[1,3*], Norsazilawati Saad[1], Sumaiyah Abdullah[1,] Erneeza Mohd Hata[4], and Johari Jalinas[4] Siti Izera Ismail[1,3*]

[1] Department of Plant Protection, Faculty of Agriculture, Universiti Putra Malaysia, Serdang 43400, Malaysia

[2] Department of Biology, Faculty of Natural and Applied Sciences, Umaru Musa Yar'adua University, P.M.B., Katsina 2218, Nigeria

[3] Laboratory of Climate-Smart Food Crop Production, Institute of Tropical Agriculture and Food Security (ITAFoS), Universiti Putra Malaysia, Serdang 43400, Malaysia

[4]Laboratory of Sustainable Agronomy & Crop Protection, Institute of Plantation Studies, Universiti Putra Malaysia, 43400 UPM Serdang, Selangor

[5] Centre for Insect Systematics, Department of Biological Sciences and Biotechnology, Faculty of Science & Technology, Universiti Kebangsaan Malaysia, Bangi 43600, Malaysia

syari@upm.edu.my (SJ); izera@upm.edu.my (S.I.I.)

Abstract

Entomopathogenic fungi (EPF) are natural enemies present in the insect population and have long been recognized as biological control agents against many insect pests. Some of the isolates have also been established as endophytes, benefiting their host plants without causing any symptoms or negative effects. Here we experimented to demonstrate two isolated fungal species of *Cordyceps javanica* and *Purpureocillium lilacinum* as an endophyte in the tomato plant by using the seed inoculation method and examining their effect on plant growth, population, and adult emergence of *B. tabaci*. Our study indicated that tomato seeds treated with a fungal suspension of *C. javanica* and *P. lilacinum* allowed their recovery up to 60 days after inoculation (DAI). Both endophytic isolates also reduced the significant population of adult *B. tabaci* in seedlings inoculated with, *C. javanica* (51.92±4.78%), and *P. lilacinum* (45.32±0.20%) compared to the control treatment (19.29±2.35). However, the adult emergence means number was significantly high in the control treatments (57.50±2.66) compared to *C. javanica* (15.00±1.47) and *P. lilacinum* (28.75±4.78) treatments. This study provides the first report on the endophytic colonization of plants by ~~fungal pathogens~~ ~~and~~ their effects on the host plants and insect herbivores in Malaysia and some neighboring countries.

Keywords: colonization, biocontrol, endophytic, *Bemisia tabaci*, *Cordyceps javanica*, and *Purpureocillium lilacinum*

Introduction

Entomopathogenic fungi (EPF) are natural enemies of the insect population and are often regarded as effective biological control agents in an integrated pest management (IPM) system [1,2]. Insect pests are usually controlled with EPF by either inundated or inoculative application of fungal propagules [3]. Several EPF has been reported to be sprayed commercially to control sap-sucking insect pests in the glasshouse [4]. In addition to direct applications, EPF has been discovered to colonize plants and grow endophytically, causing significant mortality among pest-infesting plants [5].

In many different plants, numerous genera of EPF have been isolated as endophytes. Some of them were identified as endophytes that occur naturally while others were introduced into the plant artificially using different methods of inoculation [6]. The ascomycetes fungi such as *Beauveria bassiana* and *Metarhizium anisopliae* are typical examples of fungal endophytes that naturally colonize plants and are artificially introduced into a wide variety of plants to reduce damage caused by insects pest including leaf-mining insects, and sap-sucking pests [4,7–10]. For example, the endophytic colonization of tomatoes by *B. bassiana* can significantly suppress *Tuta obsoluta* population with approximately 90% mortality [7,11].

In recent years, EPF species from the genera of *Cordyceps* and *Purpureocillium* have been reported to artificially colonized plant tissues for promoting plant growth and mortality of insect pests [12,13]. However, fungi-plant interaction has been demonstrated to last for several days after inoculation, protecting the plants against insect attacks [11,14]. Depending on the inoculation techniques, endophytes vary in their ability to colonize various plant parts and survive over a plant growth cycle [10]. Inoculation techniques include seed treatment, root dipping, stem injection soil drenching, and foliar application [15]. A range of these techniques

has been artificially studied for the endophytic colonization of tomato plants and efficiently promoting seedlings' growth and protecting them from the attack of several insect pests.

In Malaysia where this study was conducted, little or no information can be found on the effects of inoculated EPF endophytes on the host plants and insect herbivores. However, there are some reports of endophytic colonization of some plants by other fungal isolates [16,17], but no reports are yet to be available on the EPF establishment as an endophyte in tomatoes and any other plants.

The use of EPF as an artificial endophyte in tomatoes could reduce damage caused by insect pests especially whitefly, *Bemisia tabaci* which has recently become one of the major threats to tomato production in Malaysia. Therefore, the present study was conducted to evaluate the endophytic capability of tomato crops by some of the new native strains of EPF in Malaysia and their influence on plant growth, mortality, and adult emergence of *B. tabaci* as part of the biological control of whitefly infecting tomato plants.

Materials and methods

Plants, insects, and endophytic fungal isolates

Tomato seeds (MT1) used in this study were obtained from the Malaysian Agricultural Research and Development Institute (MARDI).

The stock culture of whiteflies that colonized tomato plants in the greenhouse was collected, reared, and maintained in a greenhouse, at the Faculty of Agriculture, Universiti Putra Malaysia (UPM). The *B. tabaci* were identified by using mitochondrial COI (mt COI) sequencing with the primers (C1-J-2195 and L2-N-3014) as described by Frohlich et al. [18]. The sequencing data were registered and assigned the accession number OM638559 (GenBank). *B. tabaci* population were reared on tomato plants for over 10 generations in a rearing cage at the Ladang

15, Faculty of Agriculture, UPM at (25-30°C and 60-80% RH with a photoperiod of 14L:12D h light: dark. Tomato plants were checked daily, and the damaged seedlings were replaced with new seedlings.

Two fungal isolates, *C. javanica* (Cjc-03) and *P. lilacinum* (TS-01) used in this study were derived from infected whitefly. The sequence data of the 2 isolates have been deposited in the NCBI GenBank database and the details of the isolates can be seen in the S1 table

Preparation of conidial suspension

The isolates were sub-cultured on potato dextrose agar medium (PDA) medium supplemented with 50mg/ml each of penicillin, streptomycin, and tetracycline. Conidia were harvested from 14 days old culture grown on PDA slants by flooding 0.01% of tween 80 on sporulated cultures and scrapped using a sterile spatula. Following that, the collected conidia were suspended in a 50 mL plastic tube, vortexed for 2 minutes, and filtered through four layers of muslin cloth to remove debris and create a clean stock suspension. The conidia viability was determined by inoculating 150µl of each fungal isolate suspension on the surface of fresh PDA plates [19]. Before incubation, a sterile microscope coverslip was placed on top of the agar on each plate. At 18-20 hours after incubation, the viability assessment was conducted under a light microscope at 400 magnifications by counting the number of germinated conidia out of 100 conidia in one randomly selected area. When the diameter of the conidium's germ tubes exceeds half of the diameter of the conidium, the conidia are considered viable [10]. For each isolate, the average of three replicate counts was calculated.

Tomato seed inoculation

The seed immersion method was performed in this experiment by following the method of Allegrucci et al [7]. Seeds were surface sterilized using 70% ethanol for 5 minutes and rinsed

5 times with sterile distilled water. The treated seeds were placed on sterile tissue filter papers to dry for 30 minutes in a laminar flow before being inoculated and planted [20].

About 10 g of surface-sterilized seeds were subdivided into three equal portions, out of which two portions were individually soaked in a tube containing 10 ml of a conidial suspension of $1x10^8$ conidia/ ml prepared from each of the two isolates (Cjc-03 and TS-01) (S1 Figs A and B). The third portion was soaked in sterile distilled water having 0.01% Tween 80 solution and served as a control treatment (S1 Fig C). At 24 hours after inoculation, the inoculated seeds were dried on sterile tissue paper in a laminar flow cabinet for 30 min, sown in multipots trays at a depth of 2 cm, containing approximately 20g of sterile peat moss planting media (autoclaved at 121° C for 30 minutes). The trays were maintained in the glasshouse at 25-30° C and 60-80%RH. During the experiments, plants were watered as needed and no fertilizer was used.

Assessment of endophytic colonization in inoculated tomato seedlings

Endophytic colonization was examined using two methods: (i) surface sterilization and PDA media plating, and (ii) live imaging using confocal laser-scanning microscopy (CLSM). For the first method, at each observation point, 5 seedlings were randomly selected per treatment for endophytic colonization assessment using a destructive sampling method [21]. The seedlings were then transported to the laboratory, washed, separated into leaves, stems, and roots, and trimmed into smaller pieces of about 1 cm^2 with sterile stainless scissors. These parts were surface sterilized with 70% ethanol for 5 min, rinsed 5 times in sterile distilled water, and then placed on sterile tissue paper to dry in a laminar flow cabinet [20]. Five pieces of each leaf, stem, and root from each inoculated seedling were randomly taken and placed in a petri dish containing a PDA slant, and each of the plates was labeled and replicated 3 times.

The Petri dishes containing the plant tissues were then incubated at 25±2 ° C and inspected daily to assess them for fungal growth. Plant pieces that exhibited fungal growth were recorded. A total of 60 plants and 180 plant pieces were examined and the presence or absence of fungal endophytes on leaves, stems, and roots was recorded after 15, 30, 45, and 60 DAI.

Re-isolated fungal colonies were morphologically identified by comparing the mycelia and growth pattern with the original culture and by observing the conidia and conidiophores from prepared slides using a light microscope.

The data were expressed as:

$$\text{Colonization frequency} = \left(\frac{\text{Number of Plant Pieces Colonized}}{\text{Total Number of Plant pieces}}\right) \text{x } 100$$

The second method, CLSM live imaging, was carried out as previously described by Nishi et al. [9]. A sterile stainless scissor was used to trim the sample of plant tissue to fit a glass microscope slide. CLSM observed samples within 1–2 hours of collection. about fifteen different plant parts were observed from each treatment. Selected images are representative of all images taken for a particular sample type.

Assessment of endophytic entomopathogenic fungi on tomato plant growth

The plant growth parameters: plant height, fresh weight, and dry weight were evaluated from the tomato plant used in the endophytic colonization of the tomato plant experiment as described in the above section. The height of the tomato plant was measured with a ruler at 15, 30, 45, and 60 DAI. At 60 DAI, five seedlings from each of the treated and untreated control plants were harvested and taken to the laboratory for the assessment of the fresh and dry weight of the root and aerial parts (stem and leaves). The fresh weight of the aerial part and root were weighed with an electronic balance and the same plant parts were placed inside a 20x20cm

paper bag and placed in the oven at 60⁰ C. After 3 days, the dried aerial and root parts were weighed using the same electronic balance [22].

Effect of endophytic entomopathogenic fungi on *B. tabaci* population and adult emergence

The hypothesis tested in this experiment was that EPF isolates, Cjc-03 and TS-01 applied as seed treatment dispersed with roots in peatmoss substrate to stem and root and retain the ability to infect *B. tabaci*. At 21 DAI, 6 seedlings from each treatment were transplanted in a 250ml pot (S2 Fig A). A transparent plastic container (15 cm heigh X 8 cm width) with fine mesh at the top was placed inside the rim of pots covering the aerial part of the plant (S2 Fig A). At 72 hours after transplanting, an adult whitefly was collected from a rearing cage with a manual aspirator and exposed to the aerial part of seedlings that had been grown from seeds inoculated with conidia of either *C. javanica* or *P. lilacinum* and untreated control seedlings. Afterward, 20-25 adult *B. tabaci* (≤ 5 days) were ~~placed~~ in each confined pot with seedlings. The populations of *B. tabaci* were estimated by counting the number of adults on each plant after 7 days.

~~For adult emergence, at 7 days after estimating the adult population as described above, plastic containers at the top of the pot and adult insects were removed from the aerial parts and transferred to a new cage (20 cm heigh x10 cm width) for observing adult emergence (S2 Fig B). The cages were kept in a glasshouse until after 45 days and the number of adults *B. tabaci* emerged from each treated seedling was counted and recorded.~~

Data analysis

The data of plant height were analyzed using factorial analysis of the variance, while the data for endophytic colonization, fresh and dry weight, population, and adult emergence of *B. tabaci*

were analyzed with a one-way analysis of variance (ANOVA) using the package *R* statistical software Version 3.6.1, and significant differences between the means were compared with the LSD-Fischer test (p = 0.05).

Results

Assessment of endophytic colonization in inoculated tomato Seedlings

The result of this study on endophytic colonization of tomatoes showed that all the 2 EPF isolates examined (CJc-03 and TS-01) were successfully developed as an endophyte in tomato plant (re-isolated from leaves, stem, and root) (S3 fig) following seed treatment. There was a decrease in colonization from the surface-sterilized plant tissues plated on PDA medium after 30 days of inoculation and no fungal isolate of *P. lilacinum* re-isolated at 60 days after inoculation (Fig 1). There are also no fungal isolates that were re-isolated from the control plants. However, surface sterilization is effective and successful because there is no fungal growth on the PDA medium that is plated with the last rinses of distilled water used for surface sterilization of tomato tissues.

The result of colonization frequency for both two isolates resulted in higher colonization on the stem and leaves in 15 and 30 days of assessment respectively and showed no significant difference of colonization on each day of assessment (15, 30 and 60 DAI), but significantly differ in the day interval of assessment (stem: F = 0.31 p = 0.60 ; F = 5.15 p = 0.029: leaves: F = 4.35, p = 0.06; F = 17.58, p = 0.0005) (Figs 1A and B). On the other hand, the highest root colonization by two fungal isolates was recorded at 30 days of assessment and significantly differ between the isolates on each day of assessment (15, 30 and 60 DAI) and day interval of assessment (F= 5.21 p = 0.045; F = 14.15, p = 0.001) (Fig 1C).

The endophytic colonization of the tomato plant by *C. javanica* and *P. javanica* was also observed in cortical cells of plants with confocal scanning electron micrographs (SEM) which expressed the growth of two fungal isolates on tomato plant tissues (S4 Figs A and B).

Effect of Endophytic entomopathogenic fungi on tomato seedling growth

The result of the effect of inoculated EPF on the growth of tomato plants; height, number of leaves, and fresh and dry weight are presented in fig 2 and 3. Data analyzed showed a significant increase of these parameters on tomato plants inoculated with EPF as compared to the control plant. At 15, 30, and 60 DAI, the following mean plant heights ± SE were recorded respectively: Cjc-03; 3.56 cm, 13.76 cm, 45.04 cm: TS-01; 5.90 cm, 14.06 cm, 46.28 cm (Fig 2A). The factorial analysis of variance showed significant effects of fungal endophyte, day after inoculation and their interaction on the height of tomato seedling (F =. 32.93, df = 2,32, $p < 0.001$; F = 820.91, df = 2,32, $p < 0.001$; F = 9.64, df = 4,32, $p < 0.01$. (Fig 3A).

The leaf number of tomato seedlings inoculated by Cjc-03 and TS-01 isolates showed a high and significant mean number compared to those in control tomato plants (F = 27.26, df = 2,6, $p < 0.001$) (Fig 2B).

At 60 DAI, analysis of variance (ANOVA) showed a significant increase in fresh and dry shoot and root biomass in seedlings treated seeds with Cjc-03 and TS-01 isolates as compared to the control seedlings. Fresh shoot and root showed a significant higher values weight of tomato seedlings colonized by Cjc-03 and TS-01 in comparison to the control plant (shoot of seedling F = 9.67, df = 2,6, $p = 0.013$; F = 4.83, df = 2,6, $p = 0.046$) (Figs 3A and B).

However, the mean value of the dry weight of shoot and root parts was significantly higher in fungal colonized plants than in the control plants. (Shoot dry weight: F = 9.1143, df = 2,6, p = 0.015; root dry weight: F = 3.91 df = 3,6 p = 0.05) (Fig 4 A and B).

Effect of inoculated tomato seedlings on the virulence, population, and adult emergence inhibition of *B. tabaci*

The tomato seedlings inoculated with two fungal isolates, *C. javanica* (Cjc-03) and *P. lilacinum* (TS-01) have significantly reduced the population of *B. tabaci* at 7 days after exposure to the aerial parts of the three weeks old tomato seedlings as compared with those in the control treatment. However, seedlings treated with ~~CJc-03~~ isolate caused the highest reduction (51.92±4.78%) of *B. tabaci* population than those treated with TS-01 (45.32±0.20%) isolate and there are no significant differences found among the plants treated with two isolates, but significantly differ with those of untreated control seedlings (19.29±2.35), (F = 31.36, df = 2,9, p = < 0.01) (Fig 5A).

The adult emergence rate was also recorded from the number of adults counted in the treated seedlings at 35 days after exposing adult *B. tabaci* in inoculation tomato seedlings. A significantly high mean number of adults was recorded in the control treatment (57.50±2.66) in comparison to the plants treated with ~~Cjw~~-03 (15.00±1.47) and TS-0 (28.75±4.78) (Fig 5B).

Discussion

The result of this research indicates that two isolates of EPF have the endophytic capability of tomato seedlings which could persist in roots, stems, and leaves until the end of the trials (60 days). In addition, inoculated seeds of tomatoes with *C. javanica*, and *P. lilacinum* isolates have significantly promoted plant growth and reduced the population and adult emergence of *B. tabaci* in comparison to control plants.

Previous research findings have reported the successful establishment of *C. javanica*, and *P. lilacinum* as endophytes for the control of insect pests on tomato plants and other ~~economic plants~~. Seed treatment of *Cordyceps* spp. has been successfully shown to be effective for endophytic colonization of eggplant [23], sorghum [24], and coffee [13]. Likewise, inoculation of *P. lilacinum* via seed treatment has been shown to colonize cotton [12,25].

The percentage of colonization of each isolate was shown to be proportional to the day of assessment with the second day (30 DAI) observed to have the highest percent colonization and the third day (60 DAI) to have the least percent. A similar trend was observed by Mantzoukas et al. [24] who recorded high percent colonization of sorghum leaves and stem by *B. bassiana, M. robertsii,* and *Isaria fumosorosea* at 30 DAI. Similarly, Sun et al. [23] recorded the least percentage colonization of plant tissues of eggplant (leaf, stem, and root) by *C. fumosorosea* at 60 DAI.

The current study found that tomato seed inoculated with an isolate of *C. javanica* and *P. lilacinum* promotes growth and plant biomass when compared with the uninoculated control plants. There are few reports of plant inoculations with EPF species of the genera *Cordyceps* and *Purpureocillium* promoting plant growth. For example, Sun et al. [23] reported that colonization of eggplant seedlings by *C. fumosorosea* increased plant height and other growth parameters including shoot length, root length number of leaves, and weight of fresh shoot and root. However, *P. lilacinum* has been reported to increase the number of nodes and dry biomass of cotton plants [12]. Several factors have been demonstrated fungal endophytes to enhance plants' growth (shoot length, root length number of leaves, and weight of fresh shoot and root) some of them include, the ability to nurture nutrient uptake and to withstand different biotic and abiotic stress [23,26].

The endophytic colonization of tomato by an isolate of *C. javanica* and *P. lilacinum* caused a significant reduction in whitefly populations and adult emergence following seed inoculation in comparison with the control plant. Although several studies demonstrated the significant reduction of insect pests on different host plants when inoculated with EPF [4,7,8,27], but plant inoculations with fungal pathogens in the genera of *Cordyceps* and *Purpureocellium* have been less reported to cause negative effects on *B. tabaci* and other insect pests. For instance, Sun et al. [23] reported that the inoculation of *C. fumosorosea* isolates in eggplant by seed immersion resulted in a reduction of the *B. tabaci* population. The inoculations of *B. bassiana* isolate in tomato plant by root drenching significantly reduced the number of eggs and nymphs of greenhouse whitefly, *Trialeurodes vaporariorum* (Westwood) (Hemiptera: Aleyrodidae) in comparison to the uninoculated control plants [8]. Similarly, Lopez and Sword [12], reported a reduction in the population of *Helicoverpa zea* due to the endophytic action of *B. bassiansa* and *Purpureocillium lilacinum* in cotton (*Gossypium hirsutum*).

The dead *B. tabaci* did not show any symptoms of mycosis. It has been previously reported that dead insects found in endophytically colonized plants show no symptoms of fungal infection [10]. The mechanisms underlying the negative effects caused by endophytic EPF are still largely unclear. However, based on our current study, EPF isolates, Cjc-03 and TS-01 were applied as seed treatment dispersed with roots in peatmoss substrate and retained the ability to infect *B. tabaci.*

Conclusion

In conclusion, this research provides the first report on the endophytic colonization of plants with EPF strains and its direct impact on promoting plant growth in Malaysia and some neighboring countries. Endophytic colonization has been detected with seed immersion

techniques after inoculating $1X10^8$ fungal suspension of one isolate each of *C. javanica* (Cjc-03) and *P. lilacinum* (TS-01). All the two EPF were endophytically colonized tomato seedlings and recovered during 15, 30, and 60 DAI in the leaves, stems, and roots. However, fungal endophytes were not recovered from the control of three plant parts. Moreover, the treated EPF endophyte has improved the height and biomass of tomato seedlings in comparison to those in non-treated seedlings. In addition, both endophytic isolates also reduced the population and adult emergence of *B. tabaci* on tomato seedlings as compared to the control treatment.

This research will serve as a base in Malaysia and add to several kinds of literature on the colonization of plant tissues by the EPF, and their effects on plant growth parameters. Furthermore, research on the endophytic inoculation and efficacy of these endophytic EPFs on different plants and insect pests could be utilized for the development of new IPM strategies.

Supporting information

S1 Fig. Treatments used to inoculate tomato seeds. (A). *P. lilacinum* (TS-01); (B). *C. javanica* (Cjc-03); (C) 0.01% Tween 80 solution as control.

S2 Fig. Bioassay cages used to evaluate the effect of endophytic EPF against *B. tabaci*. (A) cage used to examine the effect of endophytic EPF on *B. tabaci* Population (B) Cage used to examine Adult Emergence of *B. tabaci*

S3 Fig. Examples of endophytic EPF (Cjc-03 and TS-01) re-isolation from the tomato plant tissues. (A) Leaf, (B) stem, and (C) root tissues following seed treatment with a fungal conidia suspension

S4 Fig. Representative laser scanning confocal microscopy images of 30 days old tomato plant tissues colonized by endophytic EPF. (A) Cjc-03 (*C. javanica*) (B) TS-01 (*P. lilacinum*) with the noticeable spores indicated that the spores are alive on the plant tissues;

Germination of conidia and appressorium formation on the stem tissues of (C) Cjc-03 (*C. javanica*), (D) TS-01 (*P. lilacinum*).

S1 Table. Fungal endophytes used in the study. Species, accession number, origin, and host of EPF species

Acknowledgments:

The authors would like to express their gratitude to the Tertiary Education Trust Fund (TETFund), Nigeria for providing a Ph.D. scholarship and Universiti Putra Malaysia (UPM) for the general support during the study.

Author Contributions

Conceptualization: I.S., S.J., and J.J**.**

Data curation: S.A., N.S and I.S**.**

Formal analysis: J.J and I.S

Funding acquisition: N.S and S.J

Investigation: I.S., and E.M.H.

Methodology: J.J and I.S

Validation: S.J and S.I.I

Writing – original draft: I.S and JJ.

Writing – review & editing: S.J and S.I.I

References

1. Jiang W, Peng Y, Ye J, Wen Y, Liu G, Xie J. Effects of the entomopathogenic fungus *Metarhizium anisopliae* on the mortality and immune response of *Locusta migratoria.* Insects. 2020;11(1):1–12.

2. Sani I, Jamian S, Ismail SI, Saad N, Abdullah S, Hata EM, et al. Identification of

entomopathogenic fungi *Metarhizium anisopliae* and *Purpureocillium lilacinum* from oil palm plantation soils in Universiti Putra Malaysia. Malays J Microbiol. 2022;18(1):105–12.

3. Liu F, Huang W, Wu K, Qiu Z, Huang Y, Ling E. Exploiting Innate Immunity for Biological Pest Control. In: Advances in Insect Physiology. Academic Press Inc.; 2017. pp. 199–230. https://linkinghub.elsevier.com/retrieve/pii/S0065280617300012

4. González-Mas N, Sánchez-Ortiz A, Valverde-García P, Quesada-Moraga E. Effects of endophytic entomopathogenic ascomycetes on the life-history traits of *Aphis gossypii* glover and its interactions with melon plants. Insects. 2019;10(6):1–16.

5. Wei QY, Li YY, Xu C, Wu YX, Zhang YR, Liu H. Endophytic colonization by *Beauveria bassiana* increases the resistance of tomatoes against *Bemisia tabaci*. Arthropod Plant Interact. 2020;14(3):289–300. doi.org/10.1007/s11829-020-09746-9

6. Vega FE. Insect pathology and fungal endophytes. J Invertebr Pathol. 2008;98(3):277–9.

7. Allegrucci N, Velazquez MS, Russo ML, Perez E, Scorsetti AC. Endophytic colonisation of tomato by the entomopathogenic fungus *Beauveria bassiana*: The use of different inoculation techniques and their effects on the tomato leafminer *Tuta absoluta* (Lepidoptera: Gelechiidae). J Plant Prot Res. 2017;57(4):331–7.

8. Barra-Bucarei L, González MG, Iglesias AF, Aguayo GS, Peñalosa MG, Vera PV. *Beauveria bassiana* multifunction as an endophyte: Growth promotion and biologic control of *Trialeurodes vaporariorum,* (westwood) (hemiptera: Aleyrodidae) in tomato. Insects. 2020;11(9):1–15.

9. Nishi O, Sushida H, Higashi Y, Iida Y. Epiphytic and endophytic colonisation of tomato plants by the entomopathogenic fungus *Beauveria bassiana* strain GHA. Mycology. 2020;11:1–9. doi.org/10.1080/21501203.2019.1707723

10. Muvea AM, Meyhöfer R, Subramanian S, Poehling HM, Ekesi S, Maniania NK. Colonization of onions by endophytic fungi and their impacts on the biology of *Thrips tabaci*. PLoS One. 2014;9(9):1–7.

11. Silva ACL, Silva GA, Abib PHN, Carolino AT, Samuels RI. Endophytic colonization of tomato plants by the entomopathogenic fungus *Beauveria bassiana* for controlling the South American tomato pinworm, *Tuta absoluta*. CABI Agric Biosci. 2020;1(1):1–9. doi.org/10.1186/s43170-020-00002-x

12. Lopez DC, Sword GA. The endophytic fungal entomopathogens *Beauveria bassiana* and *Purpureocillium lilacinum* enhance the growth of cultivated cotton (*Gossypium hirsutum*) and negatively affect survival of the cotton bollworm (*Helicoverpa zea)*. Biol Control [Internet]. 2015;89:53–60. doi.org/10.1016/j.biocontrol.2015.03.010

13. Vega FE. The use of fungal entomopathogens as endophytes in biological control: a review. Mycologia. 2018;110(1):4–30.

14. Garrido-Jurado, I., Resquı´n-Romero, G., Amarilla, S.P., Rı´os-Moreno, A., Carrasco, L., Quesada-Moraga E. Transient endophytic colonization of melon plants by entomopathogenic fungi after foliar application for the control of *Bemisia tabaci* Gennadius (Hemiptera: Aleyrodidae). J Pest Sci (2004). 2017;90:319–30.

15. Mantzoukas S, Eliopoulos PA. Endophytic entomopathogenic fungi: A valuable biological control tool against plant pests. Appl Sci. 2020;10(1):1–13.

16. Hamzah TNT, Lee SY, Hidayat A, Terhem R, Faridah-Hanum I, Mohamed R. Diversity and characterization of endophytic fungi isolated from the tropical mangrove species, *Rhizophora mucronata*, and identification of potential antagonists against the soil-borne fungus, *Fusarium solani*. Front Microbiol. 2018;9(01707):1–17.

17. Siddiquee S, Yusuf UK, Zainudin NAIM. Morphological and molecular detection of *Fusarium chlamydosporum* from root endophytes of *Dendrobium crumenatum*. African

J Biotechnol. 2010;9(26):4081–90.

18. Frohlich DR, Torres-Jerez I, Bedford ID, Markham PG, Brown JK. A phylogeographical analysis of the *Bemisia tabaci* species complex based on mitochondrial DNA markers. Mol Ecol [Internet]. 1999 [cited 2022 Mar 11];8(10):1683–91. https://pubmed.ncbi.nlm.nih.gov/10583831/

19. Oliveira DGP, Pauli G, Mascarin GM, Delalibera I. A protocol for determination of conidial viability of the fungal entomopathogens *Beauveria bassiana* and *Metarhizium anisopliae* from commercial products. J Microbiol Methods. 2015;119:44–52.

20. Tefera T, Vidal S. Effect of inoculation method and plant growth medium on endophytic colonization of sorghum by the entomopathogenic fungus *Beauveria bassiana*. BioControl. 2009;54(5):663–9.

21. Bamisile BS, Senyo Akutse K, Dash CK, Qasim M, Ramos Aguila LC, Ashraf HJ, et al. Effects of Seedling Age on Colonization Patterns of Citrus limon Plants by Endophytic *Beauveria bassiana* and *Metarhizium anisopliae* and Their Influence on Seedlings Growth. J Fungi. 2020;6(1):29.

22. Canassa F, Tall S, Moral RA, Lara IAR d., Delalibera I, Meyling N V. Effects of bean seed treatment by the entomopathogenic fungi *Metarhizium robertsii* and *Beauveria bassiana* on plant growth, spider mite populations and behavior of predatory mites. Biol Control. 2019;132:199–208.

23. Sun T, Shen Z, Shaukat M, Du C, Ali S. Endophytic isolates of *Cordyceps fumosorosea* to enhance the growth of *Solanum melongena* and reduce the survival of whitefly (*Bemisia tabaci*). Insects. 2020;11(2):8–10.

24. Mantzoukas S, Chondrogiannis C, Grammatikopoulos G. Effects of three endophytic entomopathogens on sweet sorghum and on the larvae of the stalk borer *Sesamia nonagrioides*. Entomol Exp Appl. 2015;154(1):78–87.

25. Lopez DC, Zhu-Salzman K, Ek-Ramos MJ, Sword GA. The entomopathogenic fungal endophytes *Purpureocillium lilacinum* (formerly *Paecilomyces lilacinus*) and *Beauveria bassiana* negatively affect cotton aphid reproduction under both greenhouse and field conditions. PLoS One. 2014;9(8):1–8.

26. Elena GJ, Beatriz PJ, Alejandro P, Roberto LE. *Metarhizium anisopliae* (Metschnikoff) Sorokin promotes growth and has endophytic activity in tomato plants. Adv Biol Res (Rennes). 2011;5(1):22–7.

27. Rasool S, Vidkjær NH, Hooshmand K, Jensen B, Fomsgaard IS, Meyling N V. Seed inoculations with entomopathogenic fungi affect aphid populations coinciding with modulation of plant secondary metabolite profiles across plant families. New Phytol. 2021;229(3):1715–27.

Figure Click here to access/download;Figure;Figs_Manuscript.docx

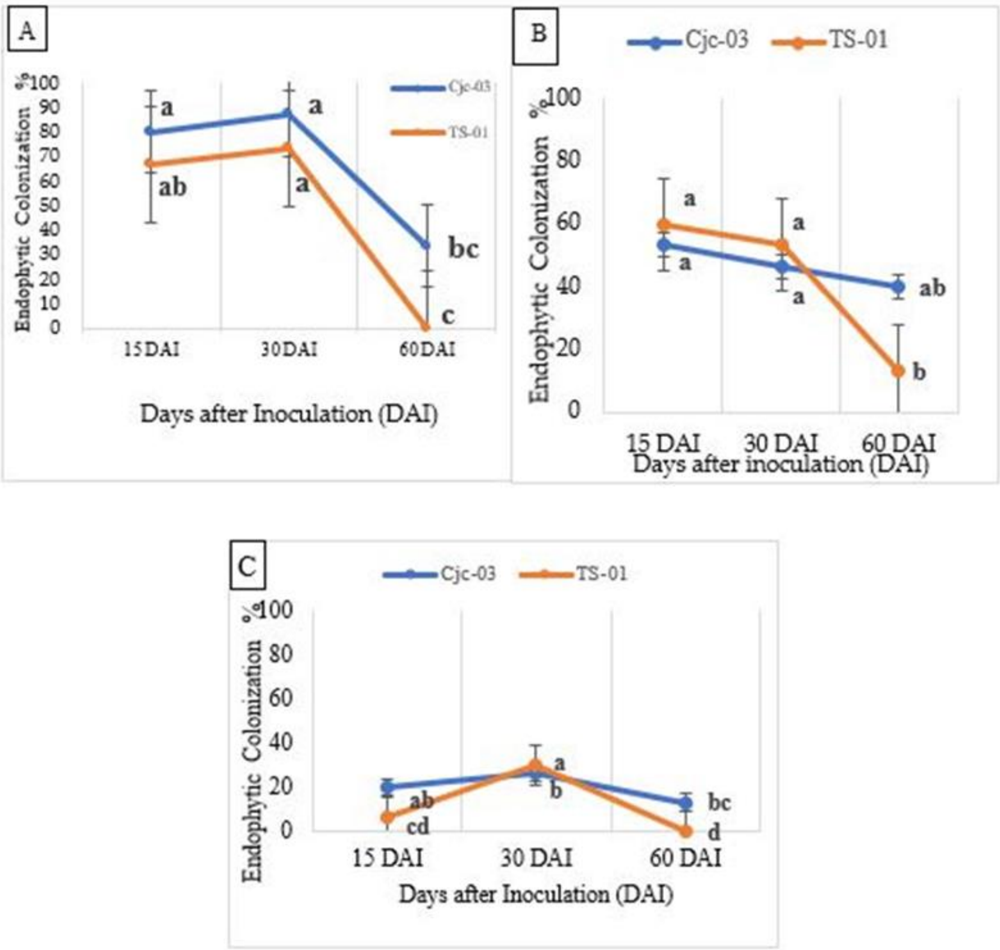

Fig 1. Percentage colonization of different plant parts (leaves, root, and stem) of tomato seedlings inoculated with fungal isolates (Cjc-03 and TS-01) at different seedlings ages (15, 30- and 45-days). (A: Leave part; B: Stem part; C: Root part).

The endophytic colonization of the tomato plant by *C. javanica* and *P. javanica* was also observed in cortical cells of plants with confocal scanning electron micrographs (SEM) which expressed the growth of two fungal isolates on tomato plant tissues (S4 Figs A and B).

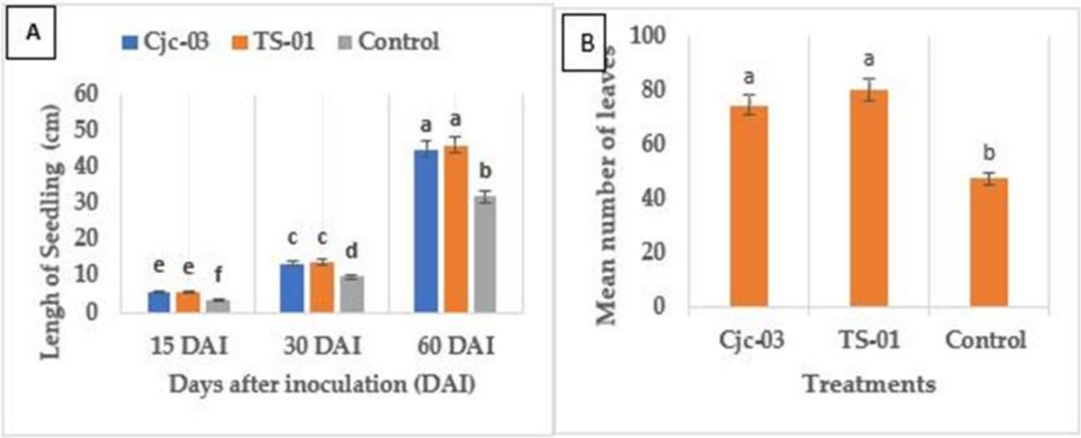

Fig 2. mean length (±SE) of tomato seedlings and number of leaves after inoculations of tomato seeds with fungal isolates (Cjc-03 and TS-01) or control (sterile distilled water containing 0.01% Tween 80). (A): Length of tomato plants measured at 15, 30, and 60 days after inoculations of tomato seeds with fungal suspension or control. (B): the number of leaves counted at 60 DAI of tomato seeds in fungal suspension or control. The different letters over the bars represent significant differences among the treatments according to Fisher's LSD test ($p < 0.05$).

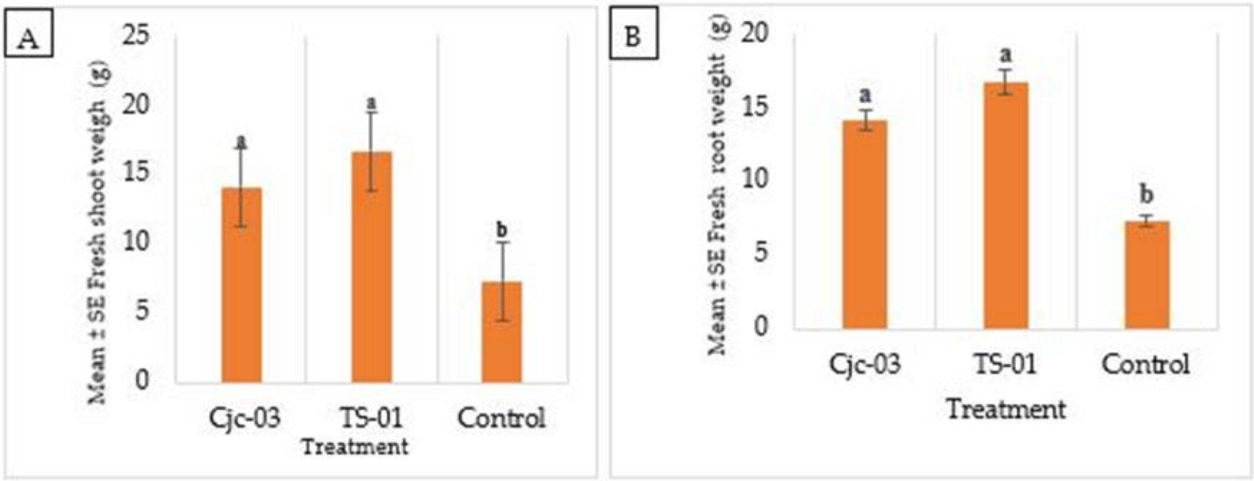

Fig 3. Mean (± SE) fresh weight (g) of tomato seedlings at 60 DAI with EPF isolates of *C.* (Cjc-03 and TS-01) or sterile distilled water containing 0.01% Tween 80 (Control). (A)

shoot part weight (B), root part weight. The different letters over the bars represent significant differences among the treatments according to Fisher's LSD test ($p < 0.05$).

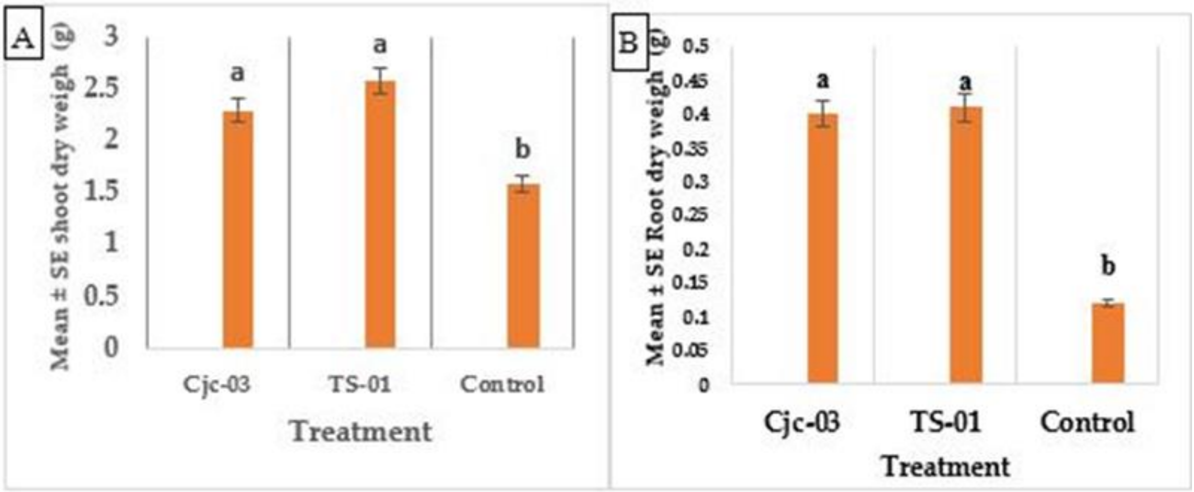

Fig 4. Mean (± SE) dry weight (g) of tomato seedlings at 60 DAI with EPF isolates of (Cjc-03 and TS-01) or sterile distilled water containing 0.01% Tween 80 (Control). (A) shoot part weight (B), root part weight. The different letters over the bars represent significant differences among the treatments according to Fisher's LSD test ($p < 0.05$).

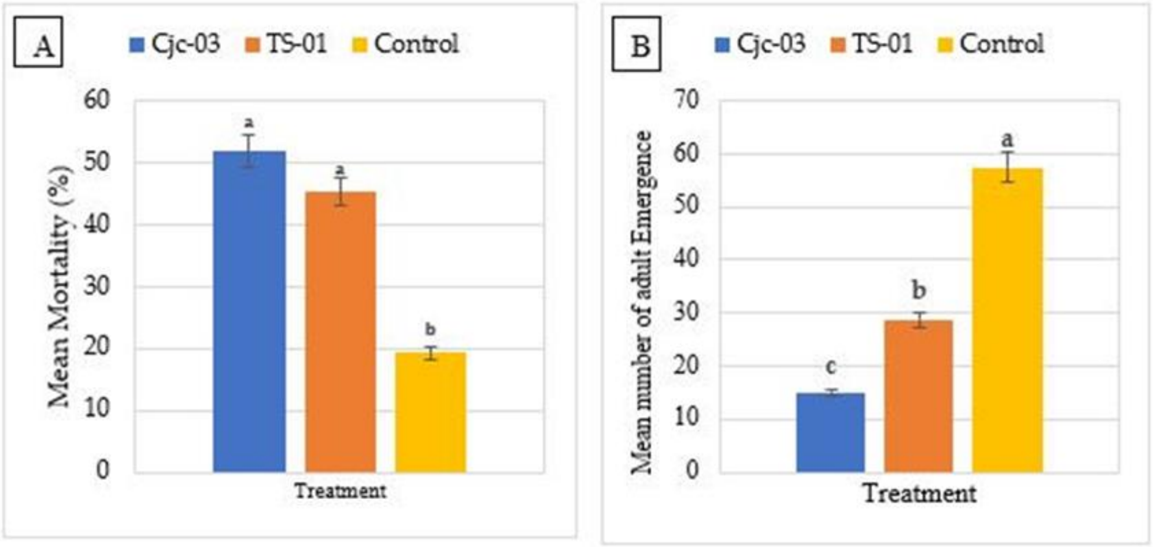

Fig 5. Effect of endophytic EPF isolates of (Cjc-03 and TS-01) on the mortality and adult emergence of *B. tabaci* exposed to the inoculated tomato seedling (A): Mean percent

mortality (SE ±) of adult *B. tabaci* exposed to tomato seedlings at 21 DAI. (B): Mean number (SE ±) of adult *B. tabaci* emergence on inoculated plants at 35 days after exposing adult *B. tabaci* on inoculated tomato seedlings. Treatments with the same letter were not significantly different according to Fisher's LSD test at $p = 0.05$.

Click here to access/download
Supporting Information
Supporting information _Plos one.docx
