# Supplementary material for: Inoculation and colonization of the entomopathogenic fungi, Isaria javanica and Purpureocillium lilacinum, in tomato plants, and their effect on seedling growth, mortality and adult emergence of Bemisia tabaci (Gennadius)
Source: PLoS One. 2023 May 22;18(5):e0285666. doi: 10.1371/journal.pone.0285666 (PMC10202273; doi:10.1371/journal.pone.0285666)
Supplement: Attachment — Submitted filename: PONE-D-22-19772_R1_reviewer (1).pdf [file pone.0285666.s010.pdf]

PLOS ONE

Inoculation and colonization of the entomopathogenic fungi, Isaria javanica and Purpureocillium lilacinum, in tomato plants, and their effect on seedling growth, mortality and adult emergence of Bemisia tabaci (Gennadius)

--Manuscript Draft--

**Manuscript Number:**	PONE-D-22-19772R1
**Article Type:**	Research Article
**Full Title:**	Inoculation and colonization of the entomopathogenic fungi, Isaria javanica and Purpureocillium lilacinum, in tomato plants, and their effect on seedling growth, mortality and adult emergence of Bemisia tabaci (Gennadius)
**Short Title:**	Endophytic entopathogenic fungi Promote growth of tomato and negatively affected Whitefly, B. tabaci
**Corresponding Author:**	Syari Jamian, Ph.D<br>UPM - Serdang: Universiti Putra Malaysia<br>Serdang, Selangor MALAYSIA
**Keywords:**	Keywords: biocontrol, endophyte, mortality, plant growth,
**Abstract:**	Entomopathogenic fungi (EPF) are natural enemies present in the insect population and have long been recognized as biological control agents against many insect pests. Some isolates have also been established as endophytes, benefiting their host plants without causing any symptoms or negative effects. Here we demonstrated two fungal species, Isaria javanica (Frieder. & Bally) Samson & Hywel-jone 2005 and Purpureocillium lilacinum (Thom) Luangsa-ard, Hou- braken, Hywel-Jones & Samson (2011) as an endophytes in tomato plants by using the seed inoculation method and examining their effect on plant growth, mortality, and adult emergence of B. tabaci.Our study indicated that tomato seeds treated with a fungal suspensions of I. javanica and P. lilacinum allowed their recovery from plant tissues (root, stem and leaf) up to 60 days after inoculation (DAI). Both endophytic isolates also significantly caused mortality of adult B. tabaci on seedlings inoculated with, I. javanica (51.92±4.78%), and P. lilacinum (45.32±0.20%) compared to the control treatment (19.29±2.35). However, the adult emergence mean number was significantly high in the control treatments (57.50±2.66) compared to I. javanica (15.00±1.47) and P. lilacinum (28.75±4.78) treatments.This study provides evidence that native endophytic isolates of I. javanica and P. lilacinum have a biocontrol potentials on whiteflies and could explored as plant growth promoters.
**Order of Authors:**	Ibrahim Sani, PhD
	Syari Jamian, Ph.D
	Norsazilawati Saad, Ph.D
	Sumaiyah Abdullah, Ph.D
	Erneeza Mohd Hata
	Johari Jalinas, Ph.D
	Siti Izera Ismail, Ph.D
**Response to Reviewers:**	The authors are very much thankful to the reviewers for their meticulous review and valuable comments. We have now incorporated all the two reviewers' comments and suggestions to improve the manuscript. Details of our response to each comment, corrections and suggestions are given in a separate file title "response of reviewer comments". However, funding information have been removed in the acknowledgments section and has been corrected as directed.
**Additional Information:**	
**Question**	**Response**
**Financial Disclosure**	This study was funded by the Fundamental Research Grant Scheme (FRGS) from the

Enter a financial disclosure statement that describes the sources of funding for the work included in this submission. Review the submission guidelines for detailed requirements. View published research articles from *PLOS ONE* for specific examples.<br><br>This statement is required for submission and **will appear in the published article** if the submission is accepted. Please make sure it is accurate.<br><br>**Unfunded studies**<br>Enter: *The author(s) received no specific funding for this work.*<br><br>**Funded studies**<br>Enter a statement with the following details:<br>• Initials of the authors who received each award<br>• Grant numbers awarded to each author<br>• The full name of each funder<br>• URL of each funder website<br>• Did the sponsors or funders play any role in the study design, data collection and analysis, decision to publish, or preparation of the manuscript?<br>• **NO** - Include this sentence at the end of your statement: *The funders had no role in study design, data collection and analysis, decision to publish, or preparation of the manuscript.*<br>• **YES** - Specify the role(s) played.<br><br>* typeset	Ministry of Higher Education Malaysia (Grant no. 5540212; reference code FRGS/1/2019/WAB01/UPM/02/36). he funders had no role in study design, data collection and analysis, decision to publish, or preparation of the manuscript.
**Competing Interests**<br><br>Use the instructions below to enter a competing interest statement for this submission. On behalf of all authors, disclose any competing interests that could be perceived to bias this work—acknowledging all financial support and any other relevant financial or non-financial competing interests.<br><br>This statement is required for submission and **will appear in the published article** if	The authors have declared that no competing interests exist.

Powered by Editorial Manager® and ProduXion Manager® from Aries Systems Corporation

the submission is accepted. Please make sure it is accurate and that any funding sources listed in your Funding Information later in the submission form are also declared in your Financial Disclosure statement.<br><br>View published research articles from *PLOS ONE* for specific examples.<br><br>**NO authors have competing interests**<br><br>Enter: *The authors have declared that no competing interests exist.*<br><br>**Authors with competing interests**<br><br>Enter competing interest details beginning with this statement:<br><br>*I have read the journal's policy and the authors of this manuscript have the following competing interests: [insert competing interests here]*<br><br>* typeset	
**Ethics Statement**<br><br>Enter an ethics statement for this submission. This statement is required if the study involved:<br><br>• Human participants<br>• Human specimens or tissue<br>• Vertebrate animals or cephalopods<br>• Vertebrate embryos or tissues<br>• Field research<br><br>Write "N/A" if the submission does not require an ethics statement.<br><br>General guidance is provided below. Consult the submission guidelines for detailed instructions. **Make sure that all information entered here is included in the Methods section of the manuscript.**	N/A

Powered by Editorial Manager® and ProduXion Manager® from Aries Systems Corporation

**Format for specific study types**<br><br>**Human Subject Research (involving human participants and/or tissue)**<br>• Give the name of the institutional review board or ethics committee that approved the study<br>• Include the approval number and/or a statement indicating approval of this research<br>• Indicate the form of consent obtained (written/oral) or the reason that consent was not obtained (e.g. the data were analyzed anonymously)<br><br>**Animal Research (involving vertebrate animals, embryos or tissues)**<br>• Provide the name of the Institutional Animal Care and Use Committee (IACUC) or other relevant ethics board that reviewed the study protocol, and indicate whether they approved this research or granted a formal waiver of ethical approval<br>• Include an approval number if one was obtained<br>• If the study involved *non-human primates*, add *additional details* about animal welfare and steps taken to ameliorate suffering<br>• If anesthesia, euthanasia, or any kind of animal sacrifice is part of the study, include briefly which substances and/or methods were applied<br><br>**Field Research**<br><br>Include the following details if this study involves the collection of plant, animal, or other materials from a natural setting:<br>• Field permit number<br>• Name of the institution or relevant body that granted permission	
**Data Availability**<br><br>Authors are required to make all data underlying the findings described fully available, without restriction, and from the time of publication. PLOS allows rare exceptions to address legal and ethical concerns. See the PLOS Data Policy and FAQ for detailed information.	Yes - all data are fully available without restriction

A Data Availability Statement describing where the data can be found is required at submission. Your answers to this question constitute the Data Availability Statement and **will be published in the article**, if accepted.<br><br>**Important:** Stating 'data available on request from the author' is not sufficient. If your data are only available upon request, select 'No' for the first question and explain your exceptional situation in the text box.<br><br>Do the authors confirm that all data underlying the findings described in their manuscript are fully available without restriction?	
**Describe where the data may be found in full sentences. If you are copying our sample text, replace any instances of XXX with the appropriate details.**<br><br>• If the data are **held or will be held in a public repository**, include URLs, accession numbers or DOIs. If this information will only be available after acceptance, indicate this by ticking the box below. For example: *All XXX files are available from the XXX database (accession number(s) XXX, XXX.*).<br>• If the data are all contained **within the manuscript and/or Supporting Information files**, enter the following: *All relevant data are within the manuscript and its Supporting Information files.*<br>• If neither of these applies but you are able to provide **details of access elsewhere**, with or without limitations, please do so. For example:<br><br>*Data cannot be shared publicly because of [XXX]. Data are available from the XXX Institutional Data Access / Ethics Committee (contact via XXX) for researchers who meet the criteria for access to confidential data.*<br><br>*The data underlying the results presented in the study are available from (include the name of the third party*	All relevant data are within the manuscript and its Supporting Information files.

and contact information or URL).<br>• This text is appropriate if the data are owned by a third party and authors do not have permission to share the data.<br><br>* typeset	
Additional data availability information:	

Powered by Editorial Manager® and ProduXion Manager® from Aries Systems Corporation

Manuscript Click here to access/download;Manuscript;Revised Manuscript.docx

Inoculation and colonization of the entomopathogenic fungi, *Isaria javanica* and *Purpureocillium lilacinum*, in tomato plants, and their effect on seedling growth, mortality and adult emergence of *Bemisia tabaci* (Gennadius)

Ibrahim Sani[1,2], Syari Jamian[1,3*], Norsazilawati Saad[1], Sumaiyah Abdullah[1], Erneeza Mohd Hata[4], Johari Jalinas[5] and Siti Izera Ismail[1,3*]

[1]Department of Plant Protection, Faculty of Agriculture, Universiti Putra Malaysia, Serdang 43400, Malaysia

[2] Department of Biology, Faculty of Natural and Applied Sciences, Umaru Musa Yar'adua University, P.M.B., Katsina 2218, Nigeria

[3] Laboratory of Climate-Smart Food Crop Production, Institute of Tropical Agriculture and Food Security (ITAFoS), Universiti Putra Malaysia, Serdang 43400, Malaysia

[4]Laboratory of Sustainable Agronomy & Crop Protection, Institute of Plantation Studies, Universiti Putra Malaysia, 43400 UPM Serdang, Selangor

[5] Centre for Insect Systematics, Department of Biological Sciences and Biotechnology, Faculty of Science & Technology, Universiti Kebangsaan Malaysia, Bangi 43600, Malaysia

syari@upm.edu.my (SJ);izera@upm.edu.my (SII)

Abstract

Entomopathogenic fungi (EPF) are natural enemies present in the insect population and have long been recognized as biological control agents against many insect pests. Some isolates have also been established as endophytes, benefiting their host plants without causing any symptoms or negative effects. Here we demonstrated two fungal species, *Isaria javanica* (Frieder. & Bally) Samson & Hywel-jone 2005 and *Purpureocillium lilacinum* (Thom) Luangsa-ard, Hou- braken, Hywel-Jones & Samson (2011) as an endophytes in tomato plants by using the seed inoculation method and examining their effect on plant growth, mortality, and adult emergence of *B. tabaci*.Our study indicated that tomato seeds treated with a fungal suspensions of *I. javanica* and *P. lilacinum* allowed their recovery from plant tissues (root, stem and leaf) up to 60 days after inoculation (DAI). Both endophytic isolates also significantly caused mortality of adult *B. tabaci* on seedlings inoculated with, *I. javanica* (51.92±4.78%), and *P. lilacinum* (45.32±0.20%) compared to the control treatment (19.29±2.35). However, the adult emergence mean number was significantly high in the control treatments (57.50±2.66) compared to *I. javanica* (15.00±1.47) and *P. lilacinum* (28.75±4.78) treatments.This study provides evidence that native endophytic isolates of *I. javanica* and *P. lilacinum* have a biocontrol potentials on whiteflies and could explored as plant growth promoters.

Keywords: biocontrol, endophyte, mortality, plant growth,

Introduction

Entomopathogenic fungi (EPF) are natural enemies of the insect population and are often regarded as effective biological control agents in an integrated pest management (IPM) system [1,2]. Insect pests are usually controlled with EPF by either inundative or inoculative application of fungal propagules [3]. Several EPF have been commercializedfor sap-sucking pests control [4]. In addition to direct applications, EPF has been discovered to colonize plants and grow endophytically, causing significant mortality among plants infested with pests including *B. tabaci* on tomato plants [5].

In many different plants, numerous genera of EPF have been isolated as endophytes. Some of them were identified as endophytes that occur naturally while others were introduced into the plant artificially using different methods of inoculation [6]. The ascomycetes fungi such as *Beauveria bassiana* and *Metarhizium anisopliae* are typical examples of fungal endophytes that naturally colonize plants and have also been artificially introduced into a wide variety of plants to reduce damage caused by insect pests including leaf-mining insects, and sap-sucking pests [4,7–10]. For example, the endophytic colonization of tomatoes by *B. bassiana* can significantly suppress *Tuta obsoluta*population causing approximately 90% mortality [7,11].

In recent years, EPF species from the genera *Isaria* and *Purpureocillium* have been reported to artificially colonized plant tissues, promoting plant growth and mortality of insect pests [12,13]. However, fungal-plant interaction has been demonstrated to last for several months after inoculation, protecting the plants against insect attacks [11,14]. Depending on the inoculation techniques, endophytes vary in their ability to colonize various plant parts and survive over a plant growth cycle [10]. Inoculation techniques include seed treatment, root dipping, stem injection soil drenching, and foliar application [15]. A range of these

techniques has been artificially studied for the endophytic colonization of tomato plants and efficiently promoting seedlings' growth and protecting them from the attack of several insect pests.

In Malaysia where this study was conducted, little or no information can be found on the effects of inoculated EPF endophytes on the host plants and insect herbivores. However, there are some reports of endophytic colonization of some plants by other fungal isolates [16,17], but no reports are yet to be available on the EPF establishment as an endophyte in tomatoes and any other plants.

The use of EPF as an artificial endophyte in tomatoes could reduce damage caused by insect pests especially the whitefly, *B. tabaci,* which has recently become one of the major threats to tomato production in Malaysia [18,19]. Therefore, the present study was conducted to evaluate the endophytic capability in tomato plants by some of the new native strains of EPF in Malaysia and their influence on plant growth, mortality, and adult emergence of *B. tabaci* as part of the IPM of whitefly..

Materials and methods

Plants, insects, and endophytic fungal isolates

Tomato seeds (MT1 variety) used in this study were obtained from the Malaysian Agricultural Research and Development Institute (MARDI). The stock culture of whiteflies that colonized tomato plants in the greenhouse was collected, reared, and maintained in a greenhouse, at the Faculty of Agriculture, Universiti Putra Malaysia (UPM). *B. tabaci* was identified by using mitochondrial COI (mt COI) sequencing with the primers (C1-J-2195 and L2-N-3014) as described by Frohlich et al. [20]. The sequencing data was registered and assigned the accession number OM638559 (GenBank). *B. tabaci* population were reared on

tomato plants for over 10 generations in a rearing cage at the Ladang 15, Faculty of Agriculture, Universiti Putra Malaysia (UPM) at (25-30°C and 60-80% RH with a photoperiod of 12L:12D h .Tomato plants were checked daily, and the damaged seedlings were replaced with new seedlings. The two fungal isolates, *I. javanica* (Cjc-03) and *P. lilacinum* (TS-01) used in this study were found naturally infesting the whiteflies, *B. tabaci* on chili and tomato plants and their virulence were evaluated against nymphs and adult *B. tabaci* under laboratory and glasshouse condition. The isolates were identified by morphological characterization and molecular identification (ITS region amplification).

The sequence data of the 2 isolates have been deposited in the NCBI GenBank database and the details of the isolates can be seen in Table S1 table

Preparation of conidial suspension

The isolates were sub-cultured on potato dextrose agar medium (PDA) medium supplemented with 50mg/ml each of penicillin, streptomycin, and tetracycline. Conidia were harvested from 14 days old culture grown on PDA slants by flooding 0.01% of tween 80 on sporulated cultures and scrapped using a sterile spatula. Following that, the collected conidia were suspended in a 50 mL plastic tube, vortexed for 2 minutes, and filtered through four layers of muslin cloth to remove debris and create a clean stock suspension. The conidia viability was determined by inoculating 150µl of each fungal isolate suspension on the surface of fresh PDA plates [19]. Before incubation, a sterile microscope coverslip was placed on top of the agar on each plate. At 18-20 hours after incubation, the viability assessment was conducted under a light microscope at 400 magnifications by counting the number of germinated conidia out of 100 conidia in one randomly selected area. When the diameter of the conidium's germ tubes exceeds half of the diameter of the conidium, the

conidia were considered viable [10]. For each isolate, an average of three replicate counts was calculated.

Tomato seed inoculation

The seed immersion method was performed in this experiment by following the method of Allegrucci et al [7]. Seeds were surface sterilized using 70% ethanol for 5 minutes and rinsed 5 times with sterile distilled water. The treated seeds were placed on sterile tissue filter papers to dry for 30 minutes in a laminar flow before being inoculated and planted [7,11,22]. About 10 g of surface-sterilized seeds were subdivided into three equal portions, out of which two portions were individually soaked in a tube containing 10 ml of a conidial suspension of $1x10^8$ conidia/ ml prepared from each of the two isolates (Cjc-03 and TS-01) . The third portion was soaked in sterile distilled water having 0.01% Tween 80 solution and served as a control treatment. At 24 hours after inoculation, the inoculated seeds were dried on sterile tissue paper in a laminar flow cabinet for 30 min, sown in multipots trays at a depth of 2 cm, containing approximately 20g of sterile peat moss planting media (autoclaved at 121° C for 30 minutes). The trays were maintained in the glasshouse at 25-30° C and 60-80%RH. During the experiments, plants were watered as needed and no fertilizer was used.

Assessment of endophytic colonization in inoculated tomato seedlings

Endophytic colonization was examined using two methods: (i) surface sterilization and PDA media plating, and (ii) live imaging using confocal laser-scanning microscopy (CLSM). For the first method, at each observation point, 5 seedlings were randomly selected per treatment for endophytic colonization assessment using a destructive sampling method [23]. The seedlings were then transported to the laboratory, washed, separated into leaves, stems, and roots, and trimmed into smaller pieces of about 1 cm^2 with sterile stainless

scissors. These parts were surface sterilized with 70% ethanol for 5 min, rinsed 5 times in sterile distilled water, and then placed on sterile tissue paper to dry in a laminar flow cabinet [22]. Five pieces of each leaf, stem, and root from each inoculated seedling were randomly taken and placed in a petri dish containing a PDA slant, and each of the plates was labeled and replicated 3 times.

The Petri dishes containing the plant tissues were then incubated at 25±2 º C and inspected daily to assess them for fungal growth. Plant pieces that exhibited fungal growth were recorded. A total of 60 plants and 180 plant pieces were examined and the presence or absence of fungal endophytes on leaves, stems, and roots was recorded after 15, 30, 45, and 60 DAI.

Re-isolated fungal colonies were morphologically identified by comparing the mycelia and growth pattern with the original culture and by observing the conidia and conidiophores from prepared slides using a light microscope.

The data were expressed as:

$$\text{Colonization frequency} = \left(\frac{\text{Number of Plant Pieces Colonized}}{\text{Total Number of Plant pieces}}\right) \text{ x } 100$$

The second method, CLSM live imaging, was carried out as previously described by Nishi et al. [9]. A sterile stainless scissor was used to trim the sample of plant tissue to fit a glass microscope slide. CLSM observed samples within 1–2 hours of collection. about fifteen different plant parts were observed from each treatment. Selected images are representative of all images taken for a particular sample type.

Assessment of endophytic entomopathogenic fungi on tomato plant growth

The plant growth parameters: plant height, fresh weight, and dry weight were evaluated from the tomato plant used in the endophytic colonization of the tomato plant experiment as described in the above section. The height of the tomato plant was measured with a ruler at 15, 30, 45, and 60 DAI. At 60 DAI, five seedlings from each of the treated and untreated control plants were harvested and taken to the laboratory for the assessment of the fresh and dry weight of the root and aerial parts (stem and leaves). The fresh weight of the aerial part and root were weighed with an electronic balance and the same plant parts were placed inside a 20x20cm paper bag and placed in the oven at 60° C. After 3 days, the dried aerial and root parts were weighed using the same electronic balance [24].

Effect of endophytic entomopathogenic fungi on *B. tabaci* mortality and adult emergence

The hypothesis tested in this experiment was that EPF isolates, Cjc-03 and TS-01 applied as seed treatment dispersed with roots in peatmoss substrate to stem and root and retain the ability to kill *B. tabaci*. At 21 DAI, 6 seedlings from each treatment were transplanted in a 250ml pot (S1 Fig A). A transparent plastic container (15 cm heigh X 8 cm width) with fine mesh at the top was placed inside the rim of pots covering the aerial part of the plant (S1 Fig A). At 72 hours after transplanting, an adult whiteflies were collected from a rearing cage with a manual aspirator and exposed to the aerial part of seedlings that had been grown from seeds inoculated with conidia of either *I. javanica* or *P. lilacinum* and untreated control seedlings. Afterward, 20-25 adult *B. tabaci* (≤ 5 days)were released in each confined pot with seedlings and adults were allowed to feed and oviposit for 7 days. The mortality of *B. tabaci* were recorded by counting the number of dead adults dropped off the plant on each

cage. At 7 days post *B. tabaci* release, all surviving adult insects were removed from the aerial parts. Eggs laid were observed and seedlings were transferred to a new cage (20 cm heigh x10 cm width) for observing adult emergence of the first-generation adults (S1 Fig B). The eggs and subsequent stages were monitored over a period of 35 days, while recording first-generation adults *B.* tabaci.

Data analysis

The data of plant height were analyzed using factorial analysis of the variance, while the data for endophytic colonization, fresh and dry weight, population, and adult emergence of *B. tabaci* were analyzed with a one-way analysis of variance (ANOVA) using the package *R* statistical software Version 3.6.1, and significant differences between the means were compared with the LSD-Fischer test (p = 0.05).

Results

Assessment of endophytic colonization of inoculated tomatoSeedlings

The result of this study on endophytic colonization of tomatoes showed that both of the EPF isolates examined (Cjc-03 and TS-01) successfully developed as endophytes in tomato plants (re-isolated from leaves, stem, and root) (S2 fig) following seed treatment. There was a decrease in colonization from the surface-sterilized plant tissues plated on PDA medium after 30 days of inoculation and no *P. lilacinum* was re-isolated at 60 days after inoculation (Fig 1). No fungal isolates were re-isolated from the control plants. However, surface sterilization is effective and successful because there was no fungal growth on the PDA medium that was plated with the last rinses of distilled water used for surface sterilization of tomato tissues.

The result of colonization frequency for both two isolates resulted in higher colonization on the stem and leaves at 15 and 30 days of assessment respectively and showed no significant difference of colonization on each day of assessment (15, 30 and 60 DAI), but significantly differed in the day interval of assessment (stem: F = 0.31 p = 0.60 ; F = 5.15 p = 0.029: leaves: F = 4.35, p = 0.06; F = 17.58, p = 0.0005) (Figs 1A and B). On the other hand, the highest root colonization by two fungal isolates was recorded at 30 days of assessment and significantly differed at each day of assessment (15, 30 and 60 DAI) (F= 5.21 p = 0.045; F = 14.15, p = 0.001) (Fig 1C).

Endophytic colonization of both isolates (Cjc-03 and TS-01) in tomato plants was also examined using live imaging by CLSM. A noticeable spore, germination of conidia and appressorium formation observed indicated that the spores are alive on the tomato plant tissues (S3 Fig.1).

Effect of Endophytic entomopathogenic fungi on tomato seedling growth

The effects of inoculated EPF on the growth of tomato plants; height, number of leaves (leaflets), and fresh and dry weight are presented in figs 2 and 3. Data analyzed showed a significant increase of these parameters on tomato plants inoculated with EPF as compared to the control plant. At 15, 30, and 60 DAI, the following mean plant heights ± SE were recorded respectively: Cjc-03; 3.56 cm, 13.76 cm, 45.04 cm: TS-01; 5.90 cm, 14.06 cm, 46.28 cm (Fig 2A).The factorial analysis of variance showed significant effects of fungal endophyte, day after inoculation and their interaction on the height of tomato seedling (F =. 32.93, df = 2,32, $p < 0.001$; F = 820.91, df = 2,32, $p < 0.001$; F = 9.64, df = 4,32, $p < 0.01$. (Fig 3A).

The leaves(leaflets) number of tomato seedlings inoculated by Cjc-03 and TS-01 isolates showed a high and significant mean number (74.67and 80.67) compared to those in control tomato plants (47.67) ($F = 27.26$, df = 2,6, $p< 0.001$) (Fig 2B).

At 60 DAI, analysis of variance (ANOVA) showed a significant increase in fresh and dry shoot and root biomass in seedlings treated seeds with Cjc-03 and TS-01 isolates as compared to the control seedlings. Fresh shoot and root showed a significant higher values weight (14.14g; 14.21 and 16.74g; 16.81g) of tomato seedlings colonized by Cjc-03 and TS-01 respectively, in comparison to the control plant (7.31g; 7.51g) (shoot of seedling $F = 9.67$, df = 2,6, $p = 0.013$; root of seedlings $F = 4.83$, df = 2,6, $p = 0.046$) (Figs 2A and B).

The mean value of the dry weight of shoot and root parts was significantly higher in fungal colonized plants than in the control plants. (Shoot dry weight: $F = 9.1143$, df = 2,6, $p = 0.015$; root dry weight: $F = 3.91$df = 3,6 $p = 0.05$) (Figs 4A and B).

Effects of endophytically-colonized tomato seedlings on the mortality, and adult emergence of *B. tabaci*

The endophytic fungal isolates, *I. javanica* (Cjc-03) and *P. lilacinum* (TS-01) have significantly increased mortality rate of *B. tabaci* feeding on colonized tomato seedlings at 7 days after exposure to the aerial parts when compared with those of the control treatment. However, seedlings treated with Cjc-03 isolate caused the highest reduction (51.92±4.78%) of *B. tabaci* population than those treated with TS-01 (45.32±0.20%) isolate and there are no significant differences found among the plants treated with two isolates, but significantly differ with those of untreated control seedlings (19.29±2.35), ($F = 31.36$, df = 2,9, $p = < 0.01$) (Fig 5A).

.

Endophytic fungi mediated reduction in adult *B. tabaci* emergence in the first generation. Both two isolates induced a significant reduction in number of adult emerged at assesment day (35 days post-exposure).A significantly high mean number of adults emergence was recorded in the control treatment (57.50±2.66) in comparison to the plants treated with Cjc-03 (15.00±1.47) and TS-0 (28.75±4.78) ($F = 43.89$, $df = 2,9$, $p = < 0.01$) (Fig 5B).

Discussion

The result of this research indicates that two EPF isolates havethe capacity to colonize tomato plants endophytically which persisted in roots, stems, and leaves until the end of the trials (60 days). In addition, inoculated tomato seeds with *I. javanica,* and *P. lilacinum* isolates have significantly promoted plant growth and reduced the population and adult emergence of *B. tabaci* in comparison to control plants.

Previous research findings have reported the successful establishment of *I. javanica,* and *P. lilacinum* as endophytes for the control of insect pests in tomato plants and other economically important plants. Seed treatment of *Isaria* spp. has been successfully shown to be effective for endophytic colonization of Pepper [25], eggplant [26], sorghum [27], and English oak [13]. Likewise, inoculation of *P. lilacinum* via seed treatment has been shown to colonize cotton [12,28].

The percentage of colonization of each isolate was shown to be proportional to the day of assessment with the second period (30 DAI) observed to have the highest percent colonization and the third period (60 DAI) to have the lowest percentage. A similar trend was observed by Mantzoukas et al. [27] who recorded high percent colonization of sorghum leaves and stem by *B. bassiana, M. robertsii,* and *Isaria fumosorosea* at 30 DAI. Similarly,

Sun et al. [26] recorded the lowest percentage colonization of plant tissues of eggplant (leaf, stem, and root) by *C. fumosorosea* at 60 DAI.

The current study found that tomato seed inoculated with an isolate of *I. javanica* or *P. lilacinum* promoted growth and plant biomass when compared with the uninoculated control plants. There are few reports of plant inoculations with EPF species of the genera *Isaria* and *Purpureocillium* promoting plant growth. For example, Sun et al. [26] reported that colonization of eggplant seedlings by *C. fumosorosea* increased plant height and other growth parameters including shoot length, root length, number of leaves, and weight of fresh shoot and root. However, *P. lilacinum* has been reported to increase the number of nodes and dry biomass of cotton plants [12]. Several factors have been demonstrated fungal endophytes to enhance plants' growth (shoot length, root length number of leaves, and weight of fresh shoot and root) some of them include, the ability to nurture nutrient uptake and to withstand different biotic and abiotic stress [26,29].

The endophytic colonization of tomato by *I. javanica* and *P. lilacinum* caused a significant reduction in whitefly populations and adult emergence following seed inoculation in comparison with the control plants. Although several studies demonstrated a significant reduction of insect pests on different host plants when inoculated with EPF [4,7,8,30], but plant inoculations with fungal pathogens of the genera of *Isaria* and *Purpureocellium*have been less reported to cause negative effects on *B. tabaci* and other insect pests. For instance, Sun et al. [26] reported that the inoculation of *C. fumosorosea* isolates in eggplant by seed immersion resulted in a reduction of the *B. tabaci* population. The inoculations of *B. bassiana* isolate in tomato plant by root drenching significantly reduced the number of eggs and nymphs of greenhouse whitefly, *Trialeurodes vaporariorum* (Westwood) (Hemiptera: Aleyrodidae) in comparison to the uninoculated control plants [8]. Similarly, Lopez and

Sword [12], reported a reduction in the population of *Helicoverpazea* due to the endophytic action of *B. bassiansa* and *Purpureocillium lilacinum* in cotton (*Gossypium hirsutum*).

The dead *B. tabaci* did not show any symptoms of mycosis. It has been previously reported that dead insects found in endophytically colonized plants show no symptoms of fungal infection [10]**.** The mechanisms underlying the negative effects of endophytic EPF on *B. tabaci* are still largely unclear. However, based on our current study, EPF isolates, Cjc-03 and TS-01 applied as seed treatment, dispersed with roots in peatmoss substrate and root and retained the ability to kill *B. tabaci.*

Conclusion

In conclusion, this research provides the first report on the endophytic colonization of plants with EPF strains and its direct impact on promoting plant growth in Malaysia and some neighboring countries. Endophytic colonization has been detected with seed immersion techniques after inoculating $1x10^8$ fungal suspension of one isolate each of *I. javanica* (Cjc-03) or *P. lilacinum* (TS-01). Both EPF colonized endophytically tomato seedlings and were recovered during 15, 30, and 60 DAI in the leaves, stems, and roots. Moreover, the plants treated with EPF endophyte have improved the height and biomass of tomato seedlings in comparison to those of non-treated seedlings. In addition, both endophytic isolates also reduced the population and adult emergence of *B. tabaci* on tomato seedlings as compared to the control treatment.

Furthermore, research on the endophytic inoculation and efficacy of EPFs on different plants and insect pests could be utilized for the development of new IPM strategies.

Supporting information

S1 Fig. Bioassay cages used to evaluate the effect of endophytic EPF against *B. tabaci*.(A) cage used to examinethe effect of endophytic EPF on *B. tabaci* Population(B)Cage used to examine Adult Emergence of *B. tabaci*

S2 Fig. Examples of endophytic EPF (Cjc-03 and TS-01) re-isolation from the tomato plant tissues. (A) Leaf, (B) stem, and (C) root tissues following seed treatment with a fungal conidia suspensions

S3 Fig. Representative laser scanning confocal microscopy images of 30 days old tomato plant tissues colonized by endophytic EPF. (A) Cjc-03 (*I. javanica*) (B) TS-01 (*P. lilacinum*) with the noticeable spores indicated that the spores are alive on the plant tissues; Germination of conidia and appressorium formation on the stem tissues of (C) Cjc-03 (*I. javanica*), (D) TS-01 (*P. lilacinum*).

S1 Table. Fungal endophytes used in the study. Species, accession number, origin, and EPF host species

Acknowledgments:

We thank Dr. Razak Terhem and Arisa Azim for technical assistance.

Author Contributions

Conceptualization: I.S., S.J., and J.J.

Data curation: S.A., N.S and I.S.

Formal analysis: J.J and I.S

Funding acquisition: N.S and S.J

Investigation: I.S., and E.M.H.

Methodology: J.J and I.S

Validation: S.J and S.I.I

Writing – original draft: I.S and JJ.

Writing – review & editing: S.J and S.I.I

References

1. Jiang W, Peng Y, Ye J, Wen Y, Liu G, Xie J. Effects of the entomopathogenic fungus *Metarhizium anisopliae* on the mortality and immune response of *Locusta migratoria.* Insects. 2020;11(1):1–12.

2. Sani I, Jamian S, Ismail SI, Saad N, Abdullah S, Hata EM, et al. Identification of entomopathogenic fungi *Metarhizium anisopliae* and *Purpureocillium lilacinum* from oil palm plantation soils in Universiti Putra Malaysia. Malays J Microbiol. 2022;18(1):105–12.

3. Liu F, Huang W, Wu K, Qiu Z, Huang Y, Ling E. Exploiting Innate Immunity for Biological Pest Control. In: Advances in Insect Physiology . Academic Press Inc.; 2017 . pp. 199–230. https://linkinghub.elsevier.com/retrieve/pii/S0065280617300012

4. González-Mas N, Sánchez-Ortiz A, Valverde-García P, Quesada-Moraga E. Effects of endophytic entomopathogenic ascomycetes on the life-history traits of *Aphis gossypii* glover and its interactions with melon plants. Insects. 2019;10(6):1–16.

5. Wei QY, Li YY, Xu C, Wu YX, Zhang YR, Liu H. Endophytic colonization by *Beauveria bassiana* increases the resistance of tomatoes against *Bemisia tabaci*. Arthropod Plant Interact . 2020;14(3):289–300. https://doi.org/10.1007/s11829-020-09746-9

6. Vega FE. Insect pathology and fungal endophytes. J Invertebr Pathol. 2008;98(3):277–9.

7. Allegrucci N, Velazquez MS, Russo ML, Perez E, Scorsetti AC. Endophytic colonisation of tomato by the entomopathogenic fungus *Beauveria bassiana:* The use of different inoculation techniques and their effects on the tomato leafminer *Tuta absoluta* (Lepidoptera: Gelechiidae). J Plant Prot Res. 2017;57(4):331–7.

8. Barra-Bucarei L, González MG, Iglesias AF, Aguayo GS, Peñalosa MG, Vera PV. *Beauveria bassiana* multifunction as an endophyte: Growth promotion and biologic control of *Trialeurodes vaporariorum*, (westwood) (hemiptera: Aleyrodidae) in tomato. Insects. 2020;11(9):1–15.

9. Nishi O, Sushida H, Higashi Y, Iida Y. Epiphytic and endophytic colonisation of tomato plants by the entomopathogenic fungus *Beauveria bassiana* strain GHA. Mycology . 2020;11:1–9. https://doi.org/10.1080/21501203.2019.1707723

10. Muvea AM, Meyhöfer R, Subramanian S, Poehling HM, Ekesi S, Maniania NK. Colonization of onions by endophytic fungi and their impacts on the biology of *Thrips tabaci*. PLoS One. 2014;9(9):1–7.

11. Silva ACL, Silva GA, Abib PHN, Carolino AT, Samuels RI. Endophytic colonization of tomato plants by the entomopathogenic fungus *Beauveria bassiana* for controlling the South American tomato pinworm, *Tuta absoluta*. CABI Agric Biosci . 2020;1(1):1–9. https://doi.org/10.1186/s43170-020-00002-x

12. Lopez DC, Sword GA. The endophytic fungal entomopathogens *Beauveria bassiana* and *Purpureocillium lilacinum* enhance the growth of cultivated cotton (*Gossypium hirsutum*) and negatively affect survival of the cotton bollworm (*Helicoverpa zea*). Biol Control . 2015;89:53–60. http://dx.doi.org/10.1016/j.biocontrol.2015.03.010

13. Vega FE. The use of fungal entomopathogens as endophytes in biological control: a

review. Mycologia. 2018;110(1):4–30.

14. Garrido-Jurado, I., Resquı´n-Romero, G., Amarilla, S.P., Rı´os-Moreno, A., Carrasco, L., Quesada-Moraga E. Transient endophytic colonization of melon plants by entomopathogenic fungi after foliar application for the control of *Bemisia tabaci* Gennadius (Hemiptera: Aleyrodidae). J Pest Sci (2004). 2017;90:319–30.

15. Mantzoukas S, Eliopoulos PA. Endophytic entomopathogenic fungi: A valuable biological control tool against plant pests. Appl Sci. 2020;10(1):1–13.

16. Hamzah TNT, Lee SY, Hidayat A, Terhem R, Faridah-Hanum I, Mohamed R. Diversity and characterization of endophytic fungi isolated from the tropical mangrove species, *Rhizophora mucronata*, and identification of potential antagonists against the soil-borne fungus, *Fusarium solani*. Front Microbiol. 2018;9(01707):1–17.

17. Siddiquee S, Yusuf UK, Zainudin NAIM. Morphological and molecular detection of *Fusarium chlamydosporum* from root endophytes of *Dendrobium crumenatum*. African J Biotechnol. 2010;9(26):4081–90.

18. Saad KA, Roff MNM, Hallett RH, Idris AB. Aphid-induced Defences in Chilli Affect Preferences of the Whitefly, *Bemisia tabaci* (Hemiptera: Aleyrodidae). Sci Rep . 2015;5(13697):1–9. http://dx.doi.org/10.1038/srep13697

19. Sani, I., Ismail, S.I., Saad, N., Abdullah, S., Jalinas, J., Jamian S. Insect Pests of Vegetables in Malaysia and their Management using Entomopathogenic Fungi. Serangga. 2020;25(3):126–43.

20. Frohlich DR, Torres-Jerez I, Bedford ID, Markham PG, Brown JK. A phylogeographical analysis of the *Bemisia tabaci* species complex based on mitochondrial DNA markers. Mol Ecol . 1999 ;8(10):1683–91. https://pubmed.ncbi.nlm.nih.gov/10583831/

21. Oliveira DGP, Pauli G, Mascarin GM, Delalibera I. A protocol for determination of conidial viability of the fungal entomopathogens *Beauveria bassiana* and *Metarhizium anisopliae* from commercial products. J Microbiol Methods. 2015;119:44–52.

22. Tefera T, Vidal S. Effect of inoculation method and plant growth medium on endophytic colonization of sorghum by the entomopathogenic fungus *Beauveria bassiana*. BioControl. 2009;54(5):663–9.

23. Bamisile BS, Senyo Akutse K, Dash CK, Qasim M, Ramos Aguila LC, Ashraf HJ, et al. Effects of Seedling Age on Colonization Patterns of Citrus limon Plants by Endophytic *Beauveria bassiana* and *Metarhizium anisopliae* and Their Influence on Seedlings Growth. J Fungi. 2020;6(1):29.

24. Canassa F, Tall S, Moral RA, Lara IAR d., Delalibera I, Meyling N V. Effects of bean seed treatment by the entomopathogenic fungi *Metarhizium robertsii* and *Beauveria bassiana* on plant growth, spider mite populations and behavior of predatory mites. Biol Control. 2019;132:199–208.

25. Bocco R, Lee M, Kim D, Ahn S, Park JW, Lee SY, et al. Endophytic *Isaria javanica* pf185 persists after spraying and controls *Myzus persicae* (Hemiptera: Aphididae) and *Colletotrichum acutatum* (Glomerellales: Glomerellaceae) in pepper. Insects. 2021;12(7):1–18.

26. Sun T, Shen Z, Shaukat M, Du C, Ali S. Endophytic isolates of *Cordyceps fumosorosea* to enhance the growth of *Solanum melongena* and reduce the survival of whitefly (*Bemisia tabaci*). Insects. 2020;11(2):8–10.

27. Mantzoukas S, Chondrogiannis C, Grammatikopoulos G. Effects of three endophytic entomopathogens on sweet sorghum and on the larvae of the stalk borer *Sesamia nonagrioides*. Entomol Exp Appl. 2015;154(1):78–87.

28. Lopez DC, Zhu-Salzman K, Ek-Ramos MJ, Sword GA. The entomopathogenic fungal endophytes *Purpureocillium lilacinum* (formerly *Paecilomyces lilacinus*) and Beauveria bassiana negatively affect cotton aphid reproduction under both greenhouse and field

conditions. PLoS One. 2014;9(8):1–8.
29. Elena GJ, Beatriz PJ, Alejandro P, Roberto LE. *Metarhizium anisopliae* (Metschnikoff) Sorokin promotes growth and has endophytic activity in tomato plants. Adv Biol Res (Rennes). 2011;5(1):22–7.
30. Rasool S, Vidkjær NH, Hooshmand K, Jensen B, Fomsgaard IS, Meyling N V. Seed inoculations with entomopathogenic fungi affect aphid populations coinciding with modulation of plant secondary metabolite profiles across plant families. New Phytol. 2021;229(3):1715–27.

Figure
Click here to access/download;Figure;Figs_PlosOne_Manuscrip_With no

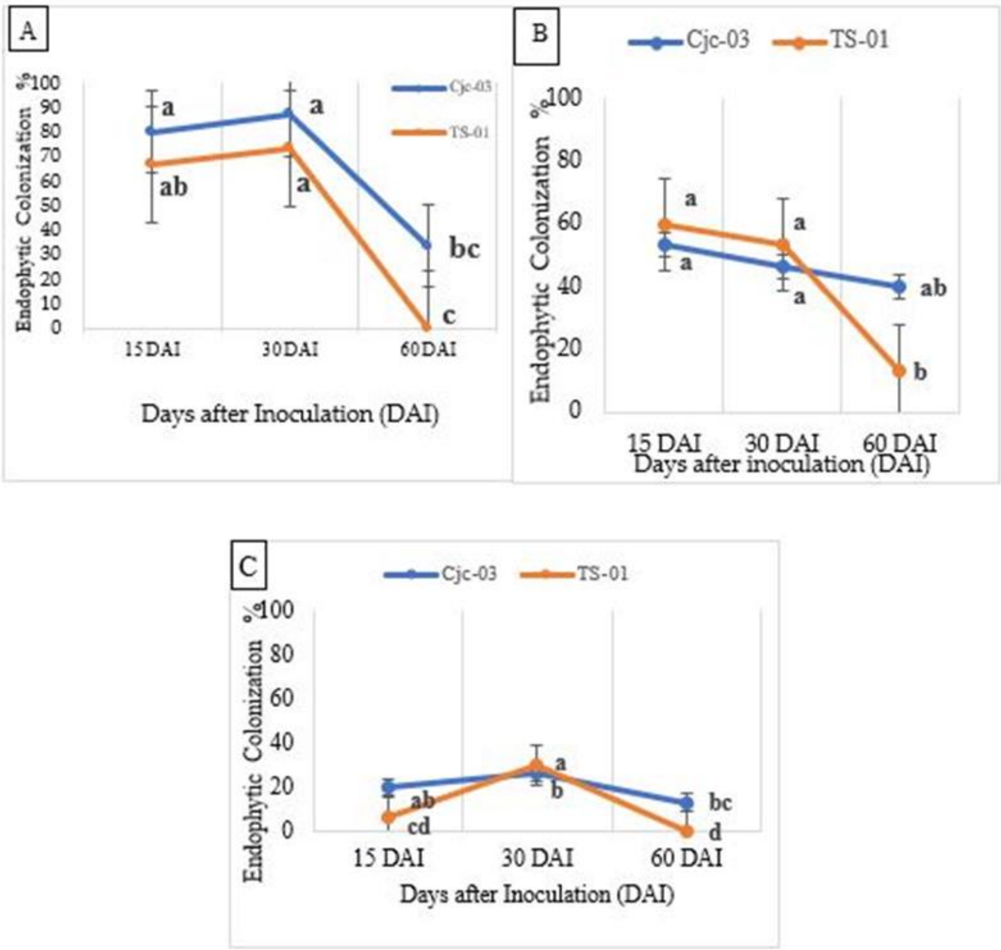

Fig 1. Percentage colonization of different plant parts (leaves, roots, and stems) of tomato seedlings inoculated with fungal isolates (Cjc-03 and TS-01) and evaluated at different time after inoculation (15, 30- and 45-days). (A: leave ; B: Stem ; C: Root).

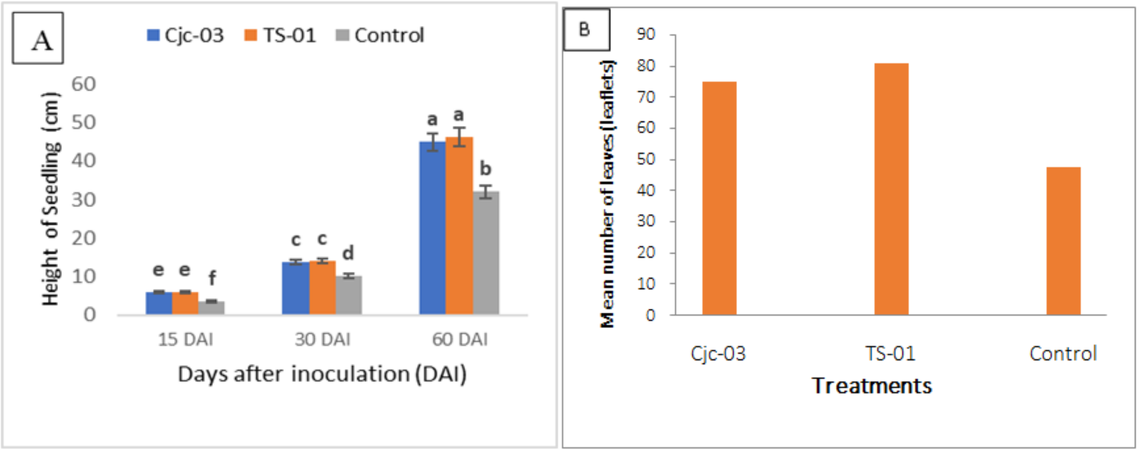

Fig 2. Mean height (±SE) of tomato seedlings and number of leaves (leaflets) after inoculations of tomato seeds with fungal isolates (Cjc-03 and TS-01) or control (sterile distilled water containing 0.01% Tween 80). (A): Length of tomato plants (shoot) measured at 15, 30, and 60 days after inoculations of tomato seeds with fungal suspension or control. (B): the number of leaves (leaflet) counted at 60 DAI of tomato seeds in fungal suspension or control. The different letters over the bars represent significant differences among the treatments according to Fisher's LSD test ($p < 0.05$).

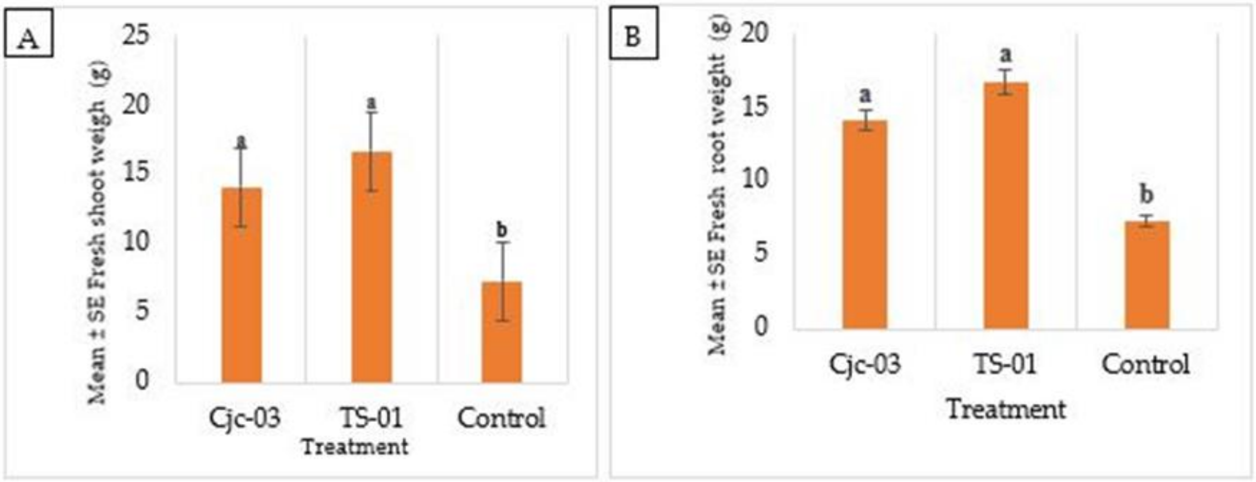

Fig 3.Mean (± SE) fresh weight (g) of tomato seedlings at 60 DAI treated with 2 EPF isolates of *C.* (Cjc-03 and TS-01) or sterile distilled water containing 0.01% Tween 80 (Control).(A) shoot weight (B), root weight. The different letters over the bars represent significant differences among the treatments according to Fisher's LSD test ($p < 0.05$).

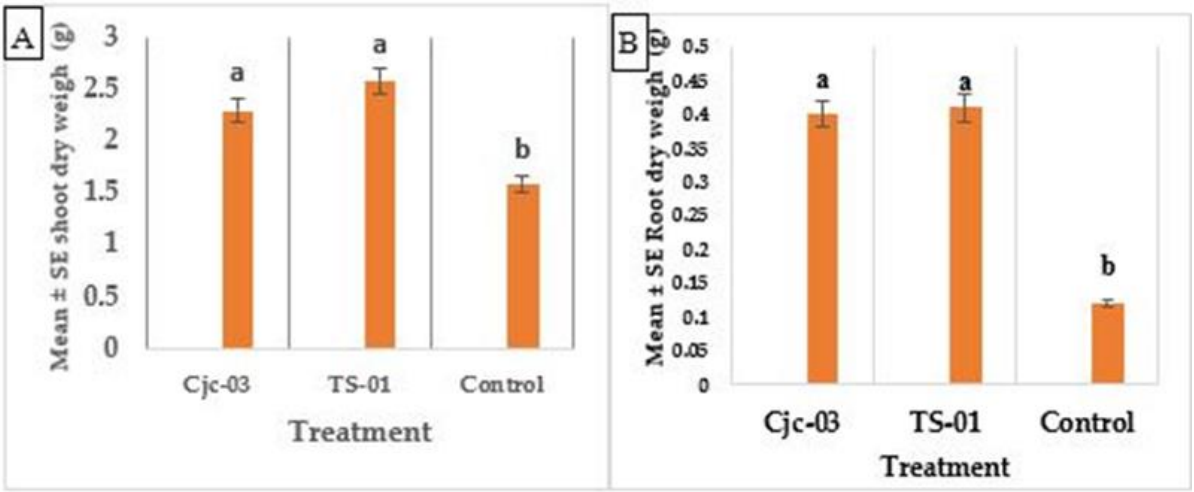

Fig 4.Mean (± SE) dry weight (g) of tomato seedlings at 60 DAI treated with 2 EPF isolates of (Cjc-03 and TS-01) or sterile distilled water containing 0.01% Tween 80 (Control). (A) Shoots weight (B), root weight. The different letters over the bars represent significant differences among the treatments according to Fisher's LSD test ($p < 0.05$).

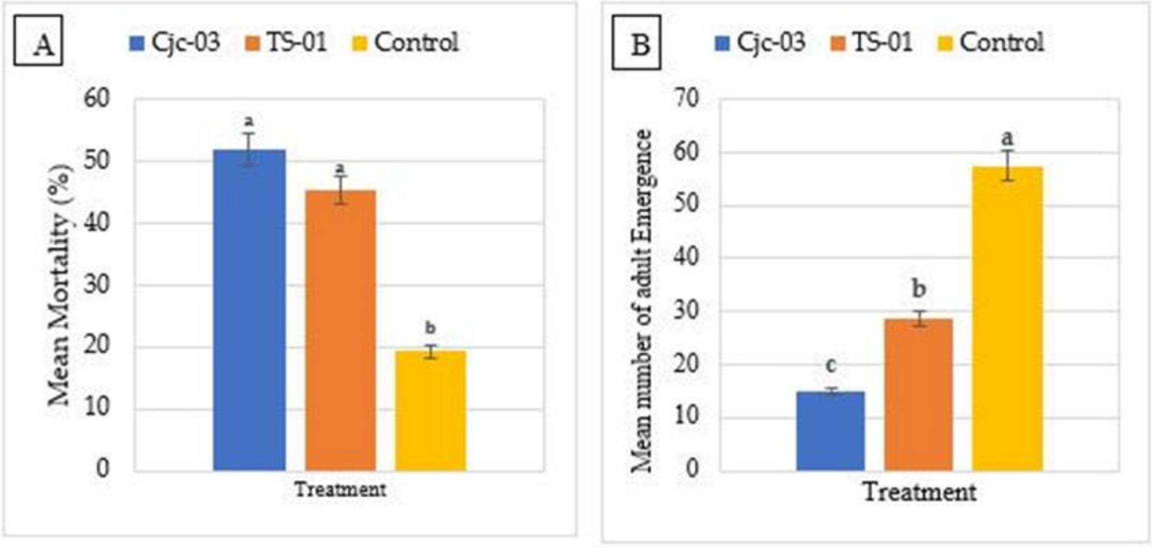

Fig 5. Effect of endophytic EPF isolates (Cjc-03 and TS-01) on the mortality and adult emergence of *B. tabaci* exposed to the inoculated tomato seedling (A): Mean percent mortality (SE ±) of adult *B. tabaci* exposed to tomato seedlings at 21 DAI. (B): Mean number (SE ±) of adult *B. tabaci* emergence on inoculated plants at 35 days after exposing adult *B.*

tabaci on inoculated tomato seedlings. Treatments with the same letter were not significantly different according to Fisher's LSD test at $p = 0.05$.

Figure Click here to access/download;Figure;Figs_Manuscript.docx

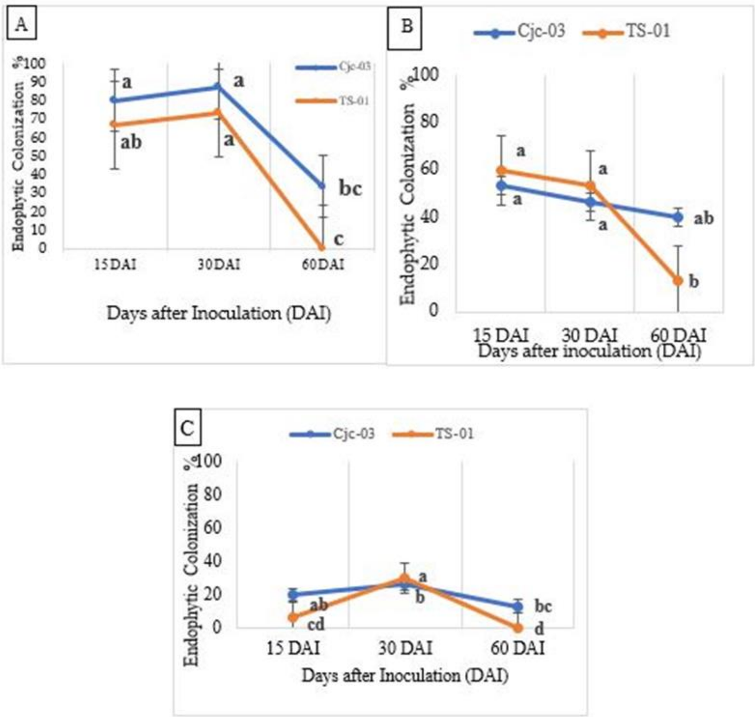

Fig 1.Percentage colonization of different plant parts (leaves, roots, and stems) of tomato seedlings inoculated with fungal isolates (Cjc-03 and TS-01) and evaluated at different time after inoculationseedlings ages (15, 30- and 45-days). (A: lLeave part; B: Stem part; C: Root part).

The endophytic colonization of the tomato plant by *C. javanica* and *P. javanica* was also observed in cortical cells of plants with confocal scanning electron micrographs (SEM) which expressed the growth of two fungal isolates on tomato plant tissues (S4 Figs A and B).

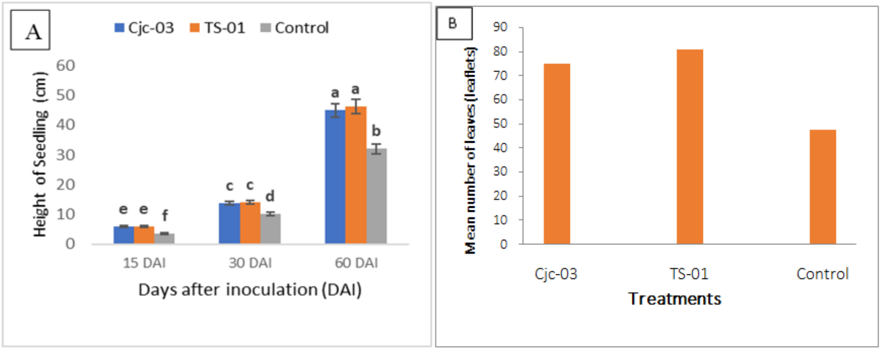

Formatted: Normal, Line spacing: single

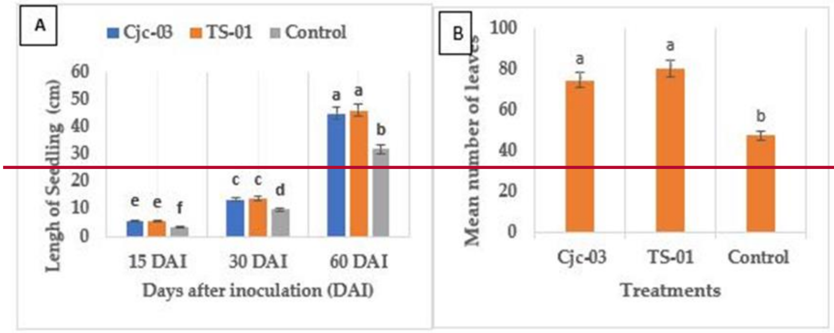

Formatted: Font: Times New Roman, 12 pt

Fig 2. mean ~~length~~height (±SE) of tomato seedlings and number of leaves (leaflets) after inoculations of tomato seeds with fungal isolates (Cjc-03 and TS-01) or control (sterile distilled water containing 0.01% Tween 80). (A): Length of tomato plants (shoot) measured at 15, 30, and 60 days after inoculations of tomato seeds with fungal suspension or control. (B): the number of leaves (leaflet) counted at 60 DAI of tomato seeds in fungal suspension or control. The different letters over the bars represent significant differences among the treatments according to Fisher's LSD test ($p < 0.05$).

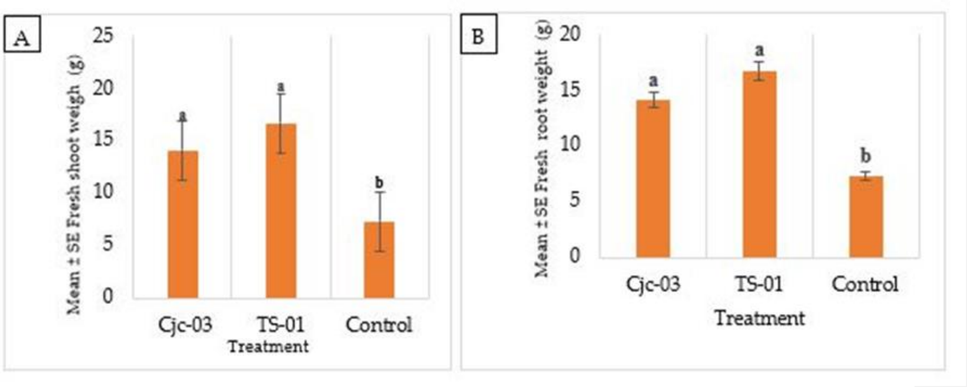

Fig 3.Mean (± SE) fresh weight (g) of tomato seedlings at 60 DAI treated with 2 EPF isolates of *C.* (Cjc-03 and TS-01) or sterile distilled water containing 0.01% Tween 80 (Control).(A) shoot ~~part~~ weight (B), root ~~part~~ weight. The different letters over the bars represent significant differences among the treatments according to Fisher's LSD test ($p < 0.05$).

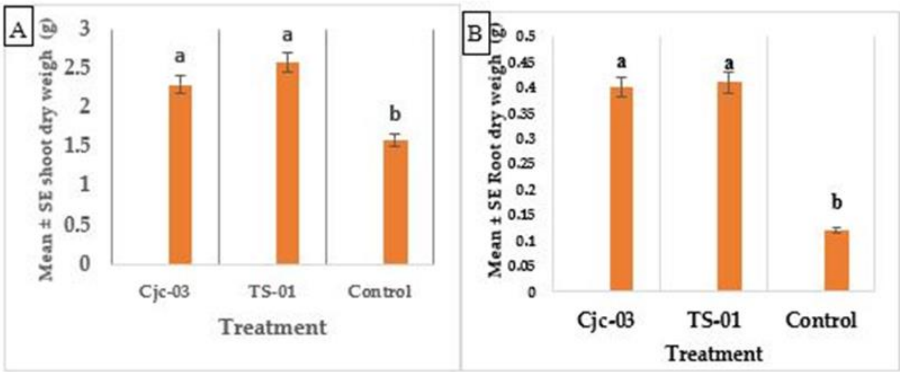

Fig 4.Mean (± SE) dry weight (g) of tomato seedlings at 60 DAI treated with 2 EPF isolates of (Cjc-03 and TS-01) or sterile distilled water containing 0.01% Tween 80 (Control). (A) ~~shoot~~Shoots ~~part~~ weight (B), root ~~part~~ weight. The different letters over the

bars represent significant differences among the treatments according to Fisher's LSD test (p < 0.05).

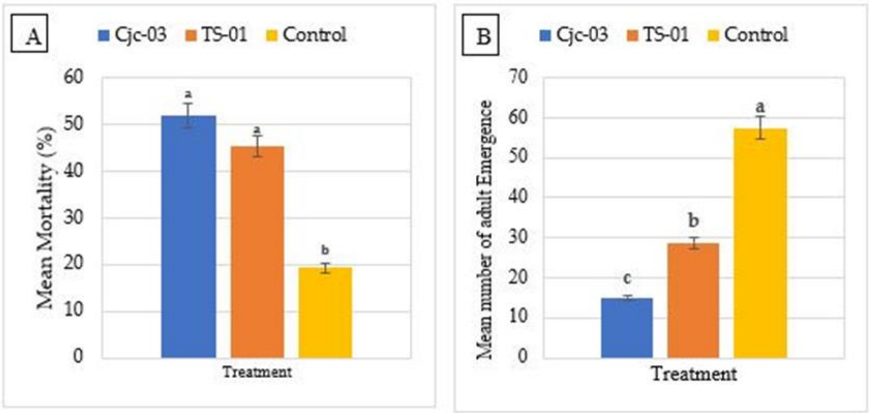

Fig 5. Effect of endophytic EPF isolates ~~of~~ (Cjc-03 and TS-01) on the mortality and adult emergence of *B. tabaci* exposed to the inoculated tomato seedling (A): Mean percent mortality (SE ±) of adult *B. tabaci* exposed to tomato seedlings at 21 DAI. (B): Mean number (SE ±) of adult *B. tabaci* emergence on inoculated plants at 35 days after exposing adult *B. tabaci* on inoculated tomato seedlings. Treatments with the same letter were not significantly different according to Fisher's LSD test at $p = 0.05$.

Click here to access/download
Supporting Information
Supporting information _No track changes.docx

Click here to access/download
Supporting Information
Supporting information _Plos one.docx

Inoculation and Colonization of the e~~E~~ntomopathogenic Fungi, ~~*Cordyceps*~~ *Isaria* *javanica* and *Purpureocillium lilacinum*, in Tomato Plants, and their effect on Seedlings Growth, Mortality~~Population,~~ and adult Emergence of ~~Whitefly,~~ *Bemisia tabaci* (Gennadius)

Ibrahim Sani[1,2], Syari Jamian[1,3*],Norsazilawati Saad[1], Sumaiyah Abdullah[1,]Erneeza Mohd Hata[4], ~~and~~ Johari Jalinas[~~5~~4] and Siti Izera Ismail[1,3*]

[1]Department of Plant Protection, Faculty of Agriculture, Universiti Putra Malaysia, Serdang 43400, Malaysia

[2] Department of Biology, Faculty of Natural and Applied Sciences, Umaru Musa Yar'adua University, P.M.B., Katsina 2218, Nigeria

[3] Laboratory of Climate-Smart Food Crop Production, Institute of Tropical Agriculture and Food Security (ITAFoS), Universiti Putra Malaysia, Serdang 43400, Malaysia

[4]Laboratory of Sustainable Agronomy & Crop Protection, Institute of Plantation Studies, Universiti Putra Malaysia, 43400 UPM Serdang, Selangor

[5] Centre for Insect Systematics, Department of Biological Sciences and Biotechnology, Faculty of Science & Technology, Universiti Kebangsaan Malaysia, Bangi 43600, Malaysia

syari@upm.edu.my (SJ);izera@upm.edu.my (S.I.I.)

Formatted: Font: (Default) Times New Roman, Bold, Kern at 18 pt

Formatted: Font: (Default) Times New Roman, Bold, Kern at 18 pt

Formatted: Font: (Default) Times New Roman, Bold, Kern at 18 pt

Formatted: Font: Bold, English (United States)

Formatted: Font: Bold, English (United States)

Formatted: Font: Bold, English (United States)

Formatted: Font: (Default) Times New Roman, 24 pt, Bold, No underline, Font color: Auto, Kern at 18 pt

Formatted: Font: (Default) Times New Roman, Kern at 18 pt

Formatted: Font: (Default) Times New Roman, No underline, Font color: Auto, Kern at 18 pt

Abstract

Entomopathogenic fungi (EPF) are natural enemies present in the insect population and have long been recognized as biological control agents against many insect pests. Some ~~of the~~ isolates have also been established as endophytes, benefiting their host plants without causing any symptoms or negative effects. Here we ~~experimented to~~ demonstrated two ~~isolated~~ fungal species, ~~of *Cordyceps*~~ *Isaria javanica* (Frieder. & Bally) Samson & Hywel-jone 2005 and *Purpureocillium lilacinum* (Thom) Luangsa-ard, Hou- braken, Hywel-Jones & Samson (2011) as an endophytes in ~~the~~ tomato plants by using the seed inoculation method and examining their effect on plant growth, ~~population~~mortality, and adult emergence of *B. tabaci*.Our study indicated that tomato seeds treated with a fungal suspensions of *I*~~*C*~~. *javanica* and *P. lilacinum* allowed their recovery from plant tissues (root, stem and leaf) up to 60 days after inoculation (DAI). Both endophytic isolates also significantly ~~reduced~~ caused mortality ~~the significant population~~ of adult *B. tabaci* o~~i~~n seedlings inoculated with, *I*~~*C*~~. *javanica* (51.92±4.78%), and *P. lilacinum* (45.32±0.20%) compared to the control treatment (19.29±2.35). However, the adult emergence mean~~s~~ number was significantly high in the control treatments (57.50±2.66) compared to *I*~~*C*~~. *javanica* (15.00±1.47) and *P. lilacinum* (28.75±4.78) treatments.This study provides evidence that native ~~the first report on the~~ endophytic isolates of *I. javanica* and *P. lilacinum* have a biocontrol potentials on whiteflies and could explored as plant growth promoters. ~~colonization of plants by fungal pathogens and their effects on the host plants and insect herbivores in Malaysia and some neighboring countries.~~

Keywords: ~~colonization,~~ biocontrol, endophyt~~eic~~, mortality, plant growth, ~~*Bemisia tabaci*, *Cordyceps javanica*, and *Purpureocillium lilacinum*~~

Formatted: Font: (Default) Times New Roman, 18 pt, Bold, No underline, Font color: Auto, Kern at 18 pt

Formatted: Font: 9 pt

Formatted: No underline, Font color: Auto

Formatted: No underline, Font color: Auto

Formatted: No underline, Font color: Auto

Formatted: No underline, Font color: Auto

Formatted: No underline, Font color: Auto

Formatted: No underline, Font color: Auto

Formatted: No underline, Font color: Auto

Formatted: No underline, Font color: Auto

Introduction

Entomopathogenic fungi (EPF) are natural enemies of the insect population and are often regarded as effective biological control agents in an integrated pest management (IPM) system [1,2]. Insect pests are usually controlled with EPF by either inundative~~ed~~ or inoculative application of fungal propagules [3]. Several EPF have~~s~~ been ~~reported to be sprayed~~ commercialized~~ly to ~~ for ~~control ~~sap-sucking ~~insect ~~pests control ~~in the glasshouse~~ [4]. In addition to direct applications, EPF has been discovered to colonize plants and grow endophytically, causing significant mortality among plants ~~pest ~~infested with pests including *B. tabaci* on tomato plants, ~~ing plants ~~[5].

In many different plants, numerous genera of EPF have been isolated as endophytes. Some of them were identified as endophytes that occur naturally while others were introduced into the plant artificially using different methods of inoculation [6]. The ascomycetes fungi such as *Beauveria bassiana* and *Metarhizium anisopliae* are typical examples of fungal endophytes that naturally colonize plants and have also been~~are~~ artificially introduced into a wide variety of plants to reduce damage caused by insect~~s~~ pests including leaf-mining insects, and sap-sucking pests [4,7–10]. For example, the endophytic colonization of tomatoes by *B. bassiana* can significantly suppress *Tuta obsoluta*population ~~with ~~causing approximately 90% mortality[7,11].

In recent years, EPF species from the genera ~~of ~~*Isaria*~~*Cordyceps*~~ and *Purpureocillium* have been reported to artificially colonized plant tissues, ~~for~~ promoting plant growth and mortality

Formatted: Font: Bold, No underline, Font color: Auto, English (United States)
Formatted: Font color: Auto
Formatted: No underline, Font color: Auto
Field Code Changed
Field Code Changed
Formatted: Not Superscript/ Subscript
Field Code Changed
Formatted: Not Superscript/ Subscript
Formatted: Font: (Default) Times New Roman, 12 pt, Font color: Auto, Not Superscript/ Subscript
Formatted: Font: 16 pt, Not Superscript/ Subscript
Field Code Changed
Formatted: Not Superscript/ Subscript
Field Code Changed
Formatted: Not Superscript/ Subscript
Field Code Changed
Formatted: Not Superscript/ Subscript
Field Code Changed
Formatted: Not Superscript/ Subscript
Formatted: Not Superscript/ Subscript
Formatted: Font color: Auto, Not Superscript/ Subscript
Formatted: Not Superscript/ Subscript

of insect pests [12,13]. However, fungal~~i~~-plant interaction has been demonstrated to last for several ~~days~~ months after inoculation, protecting the plants against insect attacks [11,14]. Depending on the inoculation techniques, endophytes vary in their ability to colonize various plant parts and survive over a plant growth cycle [10]. Inoculation techniques include seed treatment, root dipping, stem injection soil drenching, and foliar application [15]. A range of these techniques has been artificially studied for the endophytic colonization of tomato plants and efficiently promoting seedlings' growth and protecting them from the attack of several insect pests.

In Malaysia where this study was conducted, little or no information can be found on the effects of inoculated EPF endophytes on the host plants and insect herbivores. However, there are some reports of endophytic colonization of some plants by other fungal isolates [16,17], but no reports are yet to be available on the EPF establishment as an endophyte in tomatoes and any other plants.

The use of EPF as an artificial endophyte in tomatoes could reduce damage caused by insect pests especially the whitefly, *B.~~emisia~~ tabaci*, which has recently become one of the major threats to tomato production in Malaysia [18,19]. Therefore, the present study was conducted to evaluate the endophytic capability ~~of~~ in tomato ~~crops~~ plants by some of the new native strains of EPF in Malaysia and their influence on plant growth, mortality, and adult emergence of *B. tabaci* as part of the ~~biological control~~IPM of whitefly.~~ infecting tomato plants~~.

Materials and methods

Formatted: Not Superscript/ Subscript
Field Code Changed
Field Code Changed
Field Code Changed
Field Code Changed
Formatted: Not Superscript/ Subscript
Formatted: Not Superscript/ Subscript
Field Code Changed
Formatted: Not Superscript/ Subscript
Formatted: Not Superscript/ Subscript
Field Code Changed
Formatted: Not Superscript/ Subscript
Formatted: Not Superscript/ Subscript
Formatted: Font: Bold, English (United States), Not Superscript/ Subscript
Formatted: Font color: Auto

Plants, insects, and endophytic fungal isolates

Tomato seeds (MT1 variety) used in this study were obtained from the Malaysian Agricultural Research and Development Institute (MARDI).

The stock culture of whiteflies that colonized tomato plants in the greenhouse was collected, reared, and maintained in a greenhouse, at the Faculty of Agriculture, Universiti Putra Malaysia (UPM). ~~The~~ *B. tabaci* was~~ere~~ identified by using mitochondrial COI (mt COI) sequencing with the primers (C1-J-2195 and L2-N-3014) as described by Frohlich et al. [20]. The sequencing data was~~ere~~ registered and assigned the accession number OM638559 (GenBank). *B. tabaci* population were reared on tomato plants for over 10 generations in a rearing cage at the Ladang 15, Faculty of Agriculture, Universiti Putra Malaysia (UPM) at (25-30°C and 60-80% RH with a photoperiod of 12~~4~~L:12D h ~~light: dark~~.Tomato plants were checked daily, and the damaged seedlings were replaced with new seedlings.

The t~~T~~wo fungal isolates, *I*~~*C*~~*. javanica* (Cjc-03) and *P. lilacinum* (TS-01) used in this study were ~~derived from infected whitefly.~~ found naturally infesting the whiteflies, *B. tabaci* on chili and tomato plants and their virulence were evaluated against nymphs and adult *B. tabaci* under laboratory and glasshouse condition. The isolates were identified by morphological characterization and molecular identification (ITS region amplification).

The sequence data of the 2 isolates have been deposited in the NCBI GenBank database and the details of the isolates can be seen in ~~the~~ Table S1 table

Preparation of conidial suspension

The isolates were sub-cultured on potato dextrose agar medium (PDA) medium supplemented with 50mg/ml each of penicillin, streptomycin, and tetracycline. Conidia were harvested from 14 days old culture grown on PDA slants by flooding 0.01% of tween 80 on

Formatted: Font: Not Superscript/ Subscript
Formatted: Not Superscript/ Subscript
Formatted: Font color: Auto
Formatted: Not Superscript/ Subscript
Field Code Changed
Formatted: Not Superscript/ Subscript
Formatted: Not Superscript/ Subscript
Formatted: Font: (Default) Times New Roman, 12 pt, Font color: Auto, Not Superscript/ Subscript
Formatted: Font: 16 pt, Not Superscript/ Subscript
Formatted: Not Superscript/ Subscript
Formatted: Not Superscript/ Subscript
Formatted: Not Superscript/ Subscript
Formatted: Font color: Auto, Not Superscript/ Subscript
Formatted: Font color: Auto, Not Superscript/ Subscript
Formatted: Font color: Auto
Formatted: Font: Not Superscript/ Subscript
Formatted: Not Superscript/ Subscript
Formatted: Not Superscript/ Subscript

sporulated cultures and scrapped using a sterile spatula. Following that, the collected conidia were suspended in a 50 mL plastic tube, vortexed for 2 minutes, and filtered through four layers of muslin cloth to remove debris and create a clean stock suspension. The conidia viability was determined by inoculating 150µl of each fungal isolate suspension on the surface of fresh PDA plates [19]. Before incubation, a sterile microscope coverslip was placed on top of the agar on each plate. At 18-20 hours after incubation, the viability assessment was conducted under a light microscope at 400 magnifications by counting the number of germinated conidia out of 100 conidia in one randomly selected area. When the diameter of the conidium's germ tubes exceeds half of the diameter of the conidium, the conidia ~~are~~ were considered viable [10]. For each isolate, ~~the~~ an average of three replicate counts was calculated.

Tomato seed inoculation

The seed immersion method was performed in this experiment by following the method of Allegrucci et al [7].Seeds were surface sterilized using 70% ethanol for 5 minutes and rinsed 5 times with sterile distilled water. The treated seeds were placed on sterile tissue filter papers to dry for 30 minutes in a laminar flow before being inoculated and planted [7,11,22].

About 10 g of surface-sterilized seeds were subdivided into three equal portions, out of which two portions were individually soaked in a tube containing 10 ml of a conidial suspension of $1x10^8$ conidia/ ml prepared from each of the two isolates (Cjc-03 and TS-01) ~~(S1 Figs A and B)~~. The third portion was soaked in sterile distilled water having 0.01% Tween 80 solution and served as a control treatment ~~(S1 Fig C)~~. At 24 hours after inoculation, the inoculated seeds were dried on sterile tissue paper in a laminar flow cabinet for 30 min, sown in multipots trays at a depth of 2 cm, containing

Field Code Changed

Formatted: Not Superscript/ Subscript

Formatted: Not Superscript/ Subscript

Field Code Changed

Formatted: Font: Not Superscript/ Subscript

Formatted: Not Superscript/ Subscript

Field Code Changed

Field Code Changed

Formatted: Not Superscript/ Subscript

Formatted: Left, Indent: Left: 0", Hanging: 0.44", No widow/orphan control, Don't adjust space between Latin and Asian text, Don't adjust space between Asian text and numbers

Formatted: Not Superscript/ Subscript

Formatted: Not Superscript/ Subscript

approximately 20g of sterile peat moss planting media (autoclaved at 121° C for 30 minutes). The trays were maintained in the glasshouse at 25-30° C and 60-80%RH. During the experiments, plants were watered as needed and no fertilizer was used.

Assessment of endophytic colonization in inoculated tomato seedlings

Endophytic colonization was examined using two methods: (i) surface sterilization and PDA media plating, and (ii) live imaging using confocal laser-scanning microscopy (CLSM). For the first method, at each observation point, 5 seedlings were randomly selected per treatment for endophytic colonization assessment using a destructive sampling method [23]. The seedlings were then transported to the laboratory, washed, separated into leaves, stems, and roots, and trimmed into smaller pieces of about 1 cm^2 with sterile stainless scissors. These parts were surface sterilized with 70% ethanol for 5 min, rinsed 5 times in sterile distilled water, and then placed on sterile tissue paper to dry in a laminar flow cabinet [22]. Five pieces of each leaf, stem, and root from each inoculated seedling were randomly taken and placed in a petri dish containing a PDA slant, and each of the plates was labeled and replicated 3 times.

The Petri dishes containing the plant tissues were then incubated at 25±2 $^{\circ}$ C and inspected daily to assess them for fungal growth. Plant pieces that exhibited fungal growth were recorded. A total of 60 plants and 180 plant pieces were examined and the presence or absence of fungal endophytes on leaves, stems, and roots was recorded after 15, 30, 45, and 60 DAI.

Re-isolated fungal colonies were morphologically identified by comparing the mycelia and growth pattern with the original culture and by observing the conidia and conidiophores from prepared slides using a light microscope.

The data were expressed as:

$$\text{Colonization frequency} = \left(\frac{\text{Number of Plant Pieces Colonized}}{\text{Total Number of Plant pieces}}\right) \text{ x } 100$$

The second method, CLSM live imaging, was carried out as previously described by Nishi et al.[9]. A sterile stainless scissor was used to trim the sample of plant tissue to fit a glass microscope slide. CLSM observed samples within 1–2 hours of collection. about fifteen different plant parts were observed from each treatment. Selected images are representative of all images taken for a particular sample type.

Assessment of endophytic entomopathogenic fungi on tomato plant growth

The plant growth parameters: plant height, fresh weight, and dry weight were evaluated from the tomato plant used in the endophytic colonization of the tomato plant experiment as described in the above section. The height of the tomato plant was measured with a ruler at 15, 30, 45, and 60 DAI. At 60 DAI, five seedlings from each of the treated and untreated control plants were harvested and taken to the laboratory for the assessment of the fresh and dry weight of the root and aerial parts (stem and leaves). The fresh weight of the aerial part and root were weighed with an electronic balance and the same plant parts were placed inside a 20x20cm paper bag and placed in the oven at 60° C. After 3 days, the dried aerial and root parts were weighed using the same electronic balance [24].

Effect of endophytic entomopathogenic fungi on *B. tabaci* ~~population~~ mortality and adult emergence

The hypothesis tested in this experiment was that EPF isolates, Cjc-03 and TS-01 applied as seed treatment dispersed with roots in peatmoss substrate to stem and root and retain the

Formatted: Not Superscript/ Subscript

Formatted: Not Superscript/ Subscript

Formatted: Font: (Intl) Times New Roman, Not Superscript/ Subscript

Formatted: Font: (Intl) Times New Roman, Not Superscript/ Subscript

Formatted: Font: (Intl) Times New Roman, Not Superscript/ Subscript

Formatted: Not Superscript/ Subscript

Field Code Changed

Formatted: Not Superscript/ Subscript

Formatted: Font: Not Superscript/ Subscript

Formatted: Not Superscript/ Subscript

Field Code Changed

Formatted: Not Superscript/ Subscript

Formatted: Font: Not Superscript/ Subscript

Formatted: Not Superscript/ Subscript

ability to infect kill *B. tabaci*. At 21 DAI, 6 seedlings from each treatment were transplanted in a 250ml pot (S12 Fig A). A transparent plastic container (15 cm heigh X 8 cm width) with fine mesh at the top was placed inside the rim of pots covering the aerial part of the plant (S12 Fig A). At 72 hours after transplanting, an adult whitefliesy wereas collected from a rearing cage with a manual aspirator and exposed to the aerial part of seedlings that had been grown from seeds inoculated with conidia of either IC. *javanica* or *P. lilacinum* and untreated control seedlings. Afterward, 20-25 adult *B. tabaci* (≤ 5 days)were placed released in each confined pot with seedlings and adults were allowed to feed and oviposit for 7 days. The populations mortality of *B. tabaci* were estimated recorded by counting the number of dead adults dropped off the plant on each plant cageafter 7 days.

For adult emergence, aAt 7 days afterpost *B. tabaci* release, estimating the adult population as described above, plastic containers at the top of the pot and all surviving adult insects were removed from the aerial parts. Eggs laid were observed and seedlings were transferred to a new cage (20 cm heigh x10 cm width) for observing adult emergence of the first-generation adults (S12 Fig B). The eggs and subsequent stages were monitored over a period of 35 days, while recording first-generation adults *B. tabaci*. The cages were kept in a glasshouse until after 45 days and the number of adults *B. tabaci* emerged from each treated seedling was counted and recorded.

Data analysis

The data of plant height were analyzed using factorial analysis of the variance, while the data for endophytic colonization, fresh and dry weight, population, and adult emergence of *B. tabaci* were analyzed with a one-way analysis of variance (ANOVA) using the package *R* statistical software Version 3.6.1, and significant differences between the means were compared with the LSD-Fischer test (p = 0.05).

Formatted: Not Superscript/ Subscript

Formatted: Not Superscript/ Subscript

Formatted: Not Superscript/ Subscript

Formatted: Font: (Default) Times New Roman, 12 pt, Font color: Auto, Not Superscript/ Subscript

Formatted: Font: (Default) Times New Roman, Font color: Auto, Not Superscript/ Subscript

Formatted: Not Superscript/ Subscript

Formatted: Not Superscript/ Subscript

Formatted: Font color: Auto, Not Superscript/ Subscript

Formatted: Font color: Auto, Not Superscript/ Subscript

Formatted: Font color: Auto, Not Superscript/ Subscript

Formatted: Font color: Auto, Not Superscript/ Subscript

Formatted: Font color: Auto, Not Superscript/ Subscript

Formatted: Font color: Auto, Not Superscript/ Subscript

Formatted: Font color: Auto, Not Superscript/ Subscript

Formatted: Font color: Auto, Not Superscript/ Subscript

Formatted: Font color: Auto, Not Superscript/ Subscript

Formatted: Font: Not Italic, Font color: Auto, Not Superscript/ Subscript

Formatted: Not Superscript/ Subscript

Formatted: Not Superscript/ Subscript

Formatted: Not Superscript/ Subscript

Results

Assessment of endophytic colonization of~~in~~ inoculated tomato Seedlings

The result of this study on endophytic colonization of tomatoes showed that ~~all~~ both of the ~~2~~ EPF isolates examined (Cj~~J~~c-03 and TS-01) ~~were~~ successfully developed as ~~an~~ endophytes in tomato plants (re-isolated from leaves, stem, and root) (S2~~3~~ fig) following seed treatment. There was a decrease in colonization from the surface-sterilized plant tissues plated on PDA medium after 30 days of inoculation and no ~~fungal isolate of~~ *P. lilacinum* was re-isolated at 60 days after inoculation (Fig 1). ~~There are also~~ N~~n~~o fungal isolates ~~that~~ were re-isolated from the control plants. However, surface sterilization is effective and successful because there was~~is~~ no fungal growth on the PDA medium that ~~is~~ was plated with the last rinses of distilled water used for surface sterilization of tomato tissues.

The result of colonization frequency for both two isolates resulted in higher colonization on the stem and leaves ~~in~~ at 15 and 30 days of assessment respectively and showed no significant difference of colonization on each day of assessment (15, 30 and 60 DAI), but significantly differed in the day interval of assessment (stem: F = 0.31 p = 0.60 ; F = 5.15 p = 0.029: leaves: F = 4.35, p = 0.06; F = 17.58, p = 0.0005) (Figs 1A and B). On the other hand, the highest root colonization by two fungal isolates was recorded at 30 days of assessment and significantly differed ~~between the isolates on~~ at each day of assessment (15, 30 and 60 DAI) ~~and day interval of assessment~~ (F= 5.21 p = 0.045; F = 14.15, p = 0.001) (Fig 1C).

~~**The endophytic colonization of the tomato plant by *C. javanica* and *P. javanica* was also observed in cortical cells of plants with confocal scanning electron micrographs (SEM) which expressed the growth of two fungal isolates on tomato plant tissues (S4 Figs A**~~

Formatted: Font: Bold, English (United States), Not Superscript/ Subscript

Formatted: Font color: Auto

Formatted: Font: Not Superscript/ Subscript

Formatted: Not Superscript/ Subscript

Formatted: Not Superscript/ Subscript

Formatted: Not Superscript/ Subscript

Formatted: Not Superscript/ Subscript

Formatted: Font color: Auto, Not Superscript/ Subscript

Formatted: Not Superscript/ Subscript

Formatted: Font color: Auto, Not Superscript/ Subscript

Formatted: Not Superscript/ Subscript

Formatted: Not Superscript/ Subscript

Formatted: Font: (Default) Times New Roman, 12 pt, Bold, Not Superscript/ Subscript

Formatted: Normal, Don't adjust space between Latin and Asian text, Don't adjust space between Asian text and numbers

~~and B).~~Endophytic colonization of both isolates (Cjc-03 and TS-01) in tomato plants was also examined using live imaging by CLSM. A noticeable spore, germination of conidia and appressorium formation observed indicated that the spores are alive on the tomato plant tissues (S3 Fig.1).

Effect of Endophytic entomopathogenic fungi on tomato seedling growth

~~The result of T~~the effects of inoculated EPF on the growth of tomato plants; height, number of leaves (leaflets), and fresh and dry weight are presented in figs 2 and 3. Data analyzed showed a significant increase of these parameters on tomato plants inoculated with EPF as compared to the control plant. At 15, 30, and 60 DAI, the following mean plant heights ± SE were recorded respectively: Cjc-03; 3.56 cm, 13.76 cm, 45.04 cm: TS-01; 5.90 cm, 14.06 cm, 46.28 cm (Fig 2A).The factorial analysis of variance showed significant effects of fungal endophyte, day after inoculation and their interaction on the height of tomato seedling (F =. 32.93, df = 2,32, $p < 0.001$; F = 820.91, df = 2,32, $p < 0.001$; F = 9.64, df = 4,32, $p < 0.01$. (Fig 3A).

The lea~~f~~ves(leaflets) number of tomato seedlings inoculated by Cjc-03 and TS-01 isolates showed a high and significant mean number (74.67and 80.67) compared to those in control tomato plants (47.67) (F = 27.26, df = 2,6, $p < 0.001$) (Fig 2B).

At 60 DAI, analysis of variance (ANOVA) showed a significant increase in fresh and dry shoot and root biomass in seedlings treated seeds with Cjc-03 and TS-01 isolates as compared to the control seedlings. Fresh shoot and root showed a significant higher values weight (14.14g; 14.21 and 16.74g; 16.81g) of tomato seedlings colonized by Cjc-03 and TS-01 respectively, in comparison to the control plant (7.31g; 7.51g) (shoot of seedling F = 9.67, df = 2,6, $p = 0.013$; root of seedlings F = 4.83, df = 2,6, $p = 0.046$) (Figs~~2~~3A and B).

Formatted: Font: (Default) Times New Roman, 12 pt, Not Superscript/ Subscript

Formatted: Font: (Default) Times New Roman, 12 pt, English (United States), Not Superscript/ Subscript

Formatted: Font: (Default) Times New Roman, 12 pt, Not Superscript/ Subscript

Formatted: Font: (Default) Times New Roman, 12 pt, English (United States), Not Superscript/ Subscript

Formatted: Font: Italic

Formatted: Font: Not Bold, English (Malaysia), Not Superscript/ Subscript

Formatted: Font: Not Bold, English (Malaysia), Not Superscript/ Subscript

Formatted: Font: Not Bold, English (Malaysia), Not Superscript/ Subscript

Formatted: Font: Not Bold, English (Malaysia), Not Superscript/ Subscript

Formatted: Font: Not Bold, English (Malaysia), Not Superscript/ Subscript

Formatted: Font: Not Bold, English (Malaysia), Not Superscript/ Subscript

Formatted: Font: Not Bold, English (Malaysia), Not Superscript/ Subscript

Formatted: Font: Not Bold, Font color: Auto, English (Malaysia), Not Superscript/ Subscript

Formatted: Font: Not Bold, English (Malaysia), Not Superscript/ Subscript

Formatted: Font: Not Bold, English (Malaysia), Not Superscript/ Subscript

Formatted: Font: Not Bold, English (Malaysia), Not Superscript/ Subscript

~~However,~~ T~~t~~he mean value of the dry weight of shoot and root parts was significantly higher in fungal colonized plants than in the control plants. (Shoot dry weight: F = 9.1143, df = 2,6, p = 0.015; root dry weight: F = 3.91df = 3,6 p = 0.05) (Fig 4 A and B).

Effect**s** of ~~inoculated~~ endophytically-colonized tomato seedlings on the ~~virulence, population~~Mortality, and adult emergence ~~inhibition~~ of *B. tabaci*

The endophytic~~tomato seedlings inoculated with two~~ fungal isolates, *I~~C~~. javanica* (Cjc-03) and *P. lilacinum* (TS-01) have significantly ~~reduced~~ increased mortality rate ~~the population~~ of *B. tabaci* feeding on colonized tomato seedlings at 7 days after exposure to the aerial parts ~~of the three weeks old tomato seedlings~~ ~~as~~ when compared with those ~~in~~ of the control treatment. However, seedlings treated with Cj~~J~~c-03 isolate caused the highest reduction (51.92±4.78%) of *B. tabaci* population than those treated with TS-01 (45.32±0.20%) isolate and there are no significant differences found among the plants treated with two isolates, but significantly differ with those of untreated control seedlings (19.29±2.35), (F = 31.36, df = 2,9, p = < 0.01) (Fig 5A).

~~The adult emergence rate was also recorded from the number of adults counted in the treated seedlings at 35 days after exposing adult *B. tabaci* in inoculation tomato seedlings.~~

Endophytic fungi mediated reduction in adult *B. tabaci* emergence in the first generation. Both two isolates induced a significant reduction in number of adult emerged at assesment day (35 days post-exposure). ~~-~~A significantly high mean number of adults emergence was recorded in the control treatment (57.50±2.66) in comparison to the plants treated with Cjc~~w~~-03 (15.00±1.47) and TS-0 (28.75±4.78) (F = 43.89, df = 2,9, p = < 0.01) (Fig 5B).

Discussion

Formatted: Font: Not Bold, English (Malaysia), Not Superscript/ Subscript
Formatted: Font: English (Malaysia), Not Superscript/ Subscript
Formatted: Font: English (Malaysia), Not Superscript/ Subscript
Formatted: Font: English (Malaysia), Not Superscript/ Subscript
Formatted: Font: Not Bold, English (Malaysia), Not Superscript/ Subscript
Formatted: Font: Not Bold, English (Malaysia), Not Superscript/ Subscript
Formatted: Font: Not Bold, English (Malaysia), Not Superscript/ Subscript
Formatted: Font color: Auto
Formatted: Font: Not Bold, Font color: Auto, English (Malaysia), Not Superscript/ Subscript
Formatted: Font: Not Bold, English (Malaysia), Not Superscript/ Subscript
Formatted: Font: Not Bold, Font color: Auto, English (Malaysia), Not Superscript/ Subscript
Formatted: Font: Not Bold, English (Malaysia), Not Superscript/ Subscript
Formatted: Font: (Default) Times New Roman, 12 pt, Not Bold

The result of this research indicates that two EPF isolates ~~of EPF~~ have ~~the~~ capacity to colonize tomato plants endophytically ~~endophytic capability of tomato seedlings~~ which ~~could~~ persisted in roots, stems, and leaves until the end of the trials (60 days). In addition, inoculated tomato seeds ~~of tomatoes~~ with *I~~C~~. javanica*, and *P. lilacinum* isolates have significantly promoted plant growth and reduced the population and adult emergence of *B. tabaci* in comparison to control plants.

Previous research findings have reported the successful establishment of *I~~C~~. javanica*, and *P. lilacinum* as endophytes for the control of insect pests i~~o~~n tomato plants and other economically important plants. Seed treatment of ~~*Cordyceps*~~ *Isaria* spp. has been successfully shown to be effective for endophytic colonization of Pepper [25] eggplant [26], sorghum [27], and ~~coffee~~ English oak [13]. Likewise, inoculation of *P. lilacinum* via seed treatment has been shown to colonize cotton [12,28].

The percentage of colonization of each isolate was shown to be proportional to the day of assessment with the second ~~day~~ period (30 DAI) observed to have the highest percent colonization and the third ~~day~~ period (60 DAI) to have the ~~least~~ lowest percentage. A similar trend was observed by Mantzoukas et al. [27] who recorded high percent colonization of sorghum leaves and stem by *B. bassiana*, *M. robertsii*, and *Isaria fumosorosea* at 30 DAI. Similarly, Sun et al. [26] recorded the lowest~~east~~ percentage colonization of plant tissues of eggplant (leaf, stem, and root) by *C. fumosorosea* at 60 DAI.

The current study found that tomato seed inoculated with an isolate of *I~~C~~. javanica* ~~and~~ or *P. lilacinum* promoted~~s~~ growth and plant biomass when compared with the uninoculated control plants. There are few reports of plant inoculations with EPF species of the genera ~~*Cordyceps*~~ *Isaria* and *Purpureocillium* promoting plant growth. For example, Sun et al. [26] reported that colonization of eggplant seedlings by *C. fumosorosea* increased plant height and other

Formatted: Font: Not Bold, English (Malaysia), Not Superscript/ Subscript
Formatted ...
Formatted ...
Formatted ...
Formatted ...
Formatted ...
Formatted ...
Formatted ...
Formatted ...
Formatted: Font: Not Bold, Not Superscript/ Subscript
Formatted: Font: Not Bold, English (Malaysia)
Field Code Changed
Formatted ...
Field Code Changed
Formatted ...
Formatted ...
Field Code Changed
Formatted ...
Formatted ...
Formatted: Font: Not Bold, English (Malaysia)
Field Code Changed
Formatted ...
Formatted: Font: Not Bold, English (Malaysia)
Formatted ...
Field Code Changed
Formatted ...
Formatted: Font: Not Bold, English (Malaysia)
Formatted ...
Field Code Changed
Formatted ...
Formatted: Font: Not Bold, English (Malaysia)
Formatted ...
Field Code Changed
Formatted ...
Formatted ...
Formatted: Font: Not Bold, English (Malaysia)
Formatted ...
Formatted ...
Formatted ...
Formatted ...
Field Code Changed
Formatted ...
Formatted ...
Formatted: Font: Not Bold, English (Malaysia)
Formatted ...

growth parameters including shoot length, root length, number of leaves, and weight of fresh shoot and root. However, *P. lilacinum* has been reported to increase the number of nodes and dry biomass of cotton plants [12]. Several factors have been demonstrated fungal endophytes to enhance plants' growth (shoot length, root length number of leaves, and weight of fresh shoot and root) some of them include, the ability to nurture nutrient uptake and to withstand different biotic and abiotic stress [26,29].

The endophytic colonization of tomato by ~~an isolate of~~ *I~~C~~. javanica* and *P. lilacinum* caused a significant reduction in whitefly populations and adult emergence following seed inoculation in comparison with the control plants. Although several studies demonstrated ~~the~~ a significant reduction of insect pests on different host plants when inoculated with EPF [4,7,8,30], but plant inoculations with fungal pathogens ~~in~~ of the genera of ~~*Cordyceps*~~ *Isaria* and *Purpureocellium*have been less reported to cause negative effects on *B. tabaci* and other insect pests. For instance, Sun et al. [26] reported that the inoculation of *C. fumosorosea* isolates in eggplant by seed immersion resulted in a reduction of the *B. tabaci* population. The inoculations of *B. bassiana* isolate in tomato plant by root drenching significantly reduced the number of eggs and nymphs of greenhouse whitefly, *Trialeurodes vaporariorum* (Westwood) (Hemiptera: Aleyrodidae) in comparison to the uninoculated control plants [8]. Similarly, Lopez and Sword [12], reported a reduction in the population of *Helicoverpazea* due to the endophytic action of *B. bassiansa* and *Purpureocillium lilacinum* in cotton (*Gossypium hirsutum*).

The dead *B. tabaci* did not show any symptoms of mycosis. It has been previously reported that dead insects found in endophytically colonized plants show no symptoms of fungal infection [10]. The mechanisms underlying the negative effects of~~caused by~~ endophytic EPF on *B. tabaci* are still largely unclear. However, based on our current study, EPF isolates, Cjc-

Field Code Changed

Formatted: Font: Not Bold, English (Malaysia), Not Superscript/ Subscript

Formatted: Font: Not Bold, English (Malaysia)

Formatted: Font: Not Bold, English (Malaysia), Not Superscript/ Subscript

Field Code Changed

Formatted: Font: Not Bold, English (Malaysia), Not Superscript/ Subscript

Formatted: Font: Not Bold, English (Malaysia)

Formatted: Font: Not Bold, English (Malaysia), Not Superscript/ Subscript

Formatted: Font: Not Bold, English (Malaysia), Not Superscript/ Subscript

Formatted: Font: Not Bold, English (Malaysia), Not Superscript/ Subscript

Field Code Changed

Formatted: Font: Not Bold, English (Malaysia), Not Superscript/ Subscript

Formatted: Font: Not Bold, English (Malaysia)

Formatted: Font: Not Bold, English (Malaysia), Not Superscript/ Subscript

Formatted: Font: Not Bold, English (Malaysia), Not Superscript/ Subscript

Field Code Changed

Formatted: Font: Not Bold, English (Malaysia), Not Superscript/ Subscript

Formatted: Font: Not Bold, English (Malaysia), Not Superscript/ Subscript

Formatted: Font: Not Bold, English (Malaysia)

Formatted: Font: Not Bold, English (Malaysia), Not Superscript/ Subscript

Formatted: Font: Not Bold, English (Malaysia), Not Superscript/ Subscript

Field Code Changed

Formatted: Font: Not Bold, English (Malaysia), Not Superscript/ Subscript

Formatted: Font: Not Bold, English (Malaysia)

Field Code Changed

Formatted: Font: Not Bold, English (Malaysia), Not Superscript/ Subscript

Formatted: Font: Not Bold, English (Malaysia)

Formatted: Font: Not Bold, English (Malaysia), Not Superscript/ Subscript

Formatted: Font: Not Bold, English (Malaysia), Not Superscript/ Subscript

Field Code Changed

Formatted: Font: Not Bold, English (Malaysia), Not Superscript/ Subscript

Formatted: Font: Not Bold, English (Malaysia)

Formatted: Font: Not Bold, English (Malaysia), Not Superscript/ Subscript

03 and TS-01 ~~were~~ applied as seed treatment, dispersed with roots in peatmoss substrate and root and retained the ability to ~~infect~~ kill *B. tabaci*.

Conclusion

In conclusion, this research provides the first report on the endophytic colonization of plants with EPF strains and its direct impact on promoting plant growth in Malaysia and some neighboring countries. Endophytic colonization has been detected with seed immersion techniques after inoculating 1x~~X~~10^8 fungal suspension of one isolate each of *I*~~*C*~~*. javanica* (Cjc-03) ~~and~~ or *P. lilacinum* (TS-01). ~~All the two~~Both EPF ~~were~~ colonized endophytically ~~colonized~~ tomato seedlings and were recovered during 15, 30, and 60 DAI in the leaves, stems, and roots. ~~However, fungal endophytes were not recovered from the control of three plant parts.~~ Moreover, the plants treated with EPF endophyte ha~~ve~~s improved the height and biomass of tomato seedlings in comparison to those ~~in~~ of non-treated seedlings. In addition, both endophytic isolates also reduced the population and adult emergence of *B. tabaci* on tomato seedlings as compared to the control treatment.

~~This research will serve as a base in Malaysia and add to several kinds of literature on the colonization of plant tissues by the EPF, and their effects on plant growth parameters.~~ Furthermore, research on the endophytic inoculation and efficacy of ~~these endophytic~~ EPFs on different plants and insect pests could be utilized for the development of new IPM strategies.

Supporting information

Formatted: Font: Not Bold, Font color: Auto, English (Malaysia), Not Superscript/ Subscript

Formatted: Font: Not Bold, English (Malaysia), Not Superscript/ Subscript

Formatted: Font: Not Bold, Font color: Auto, English (Malaysia), Not Superscript/ Subscript

Formatted: Font: Not Bold, English (Malaysia), Not Superscript/ Subscript

Formatted: Font: Italic

Formatted: Font: Not Bold, English (Malaysia), Not Superscript/ Subscript

Formatted: Font: Not Bold, English (Malaysia)

Formatted: Font: Not Bold, English (Malaysia), Not Superscript/ Subscript

Formatted: Font: Not Bold, English (Malaysia), Not Superscript/ Subscript

Formatted: Font: Not Bold, English (Malaysia), Not Superscript/ Subscript

Formatted: Font: Not Bold, English (Malaysia), Not Superscript/ Subscript

Formatted: Font: English (Malaysia), Not Superscript/ Subscript, Kern at 18 pt

S1 Fig. Treatments used to inoculate tomato seeds.(A). *P. lilacinum* (TS-01); (B). *C. javanica* (Cjc-03); (C) 0.01% Tween 80 solution as control.

S12 Fig. Bioassay cages used to evaluate the effect of endophytic EPF against *B. tabaci*.(A) cage used to examinethe effect of endophytic EPF on *B. tabaci* Population(B)Cage used to examine Adult Emergence of *B. tabaci*

S23 Fig. Examples of endophytic EPF (Cjc-03 and TS-01) re-isolation from the tomato plant tissues. (A) Leaf, (B) stem, and (C) root tissues following seed treatment with a fungal conidia suspensions

S34 Fig. Representative laser scanning confocal microscopy images of 30 days old tomato plant tissues colonized by endophytic EPF. (A) Cjc-03 (*IC. javanica*) (B) TS-01 (*P. lilacinum*)with the noticeable spores indicated that the spores are alive on the plant tissues; Germination of conidia and appressorium formation on the stem tissues of (C) Cjc-03 (*IC. javanica*), (D) TS-01 (*P. lilacinum*).

S1 Table. Fungal endophytes used in the study. Species, accession number, origin, and host of EPF host species

Acknowledgments:

We thank Dr. Razak Terhem and Arisa Azim for technical assistance. The authors would like to express their gratitude to the Tertiary Education Trust Fund (TETFund), Nigeria for providing a Ph.D. scholarship and Universiti Putra Malaysia (UPM) for the general support during the study.

Author Contributions

Conceptualization: I.S., S.J., and J.J.

Data curation: S.A., N.S and I.S.

Formatted: English (Malaysia), Not Superscript/ Subscript
Formatted: Font: Not Bold, English (Malaysia), Not Superscript/ Subscript
Formatted: English (Malaysia), Not Superscript/ Subscript
Formatted: English (Malaysia), Not Superscript/ Subscript
Formatted: Font color: Auto, English (Malaysia), Not Superscript/ Subscript
Formatted: English (Malaysia), Not Superscript/ Subscript
Formatted: Font: Not Bold, English (Malaysia), Not Superscript/ Subscript
Formatted: Font: Not Bold, English (Malaysia), Not Superscript/ Subscript
Formatted: Font: Not Bold, English (Malaysia), Not Superscript/ Subscript
Formatted: Font: Not Bold, Font color: Auto, English (Malaysia), Not Superscript/ Subscript
Formatted: Font: Not Bold, English (Malaysia), Not Superscript/ Subscript
Formatted: Font: Not Bold, Font color: Auto, English (Malaysia), Not Superscript/ Subscript
Formatted: Font: Not Bold, English (Malaysia), Not Superscript/ Subscript
Formatted: English (Malaysia), Not Superscript/ Subscript
Formatted: English (Malaysia), Not Superscript/ Subscript
Formatted: Font: Not Bold, English (Malaysia), Not Superscript/ Subscript
Formatted: Not Superscript/ Subscript
Formatted: Not Superscript/ Subscript
Formatted: Not Superscript/ Subscript
Formatted: Not Superscript/ Subscript
Formatted: Not Superscript/ Subscript
Formatted: Not Superscript/ Subscript
Formatted: Not Superscript/ Subscript
Formatted: Not Superscript/ Subscript
Formatted: English (Malaysia), Not Superscript/ Subscript
Formatted: Font: Not Bold, English (Malaysia), Not Superscript/ Subscript
Formatted: Font: Not Superscript/ Subscript
Formatted: Font color: Auto
Formatted: Font: Not Bold, Not Superscript/ Subscript
Formatted: Font color: Auto
Formatted: English (Malaysia), Not Superscript/ Subscript
Formatted: English (Malaysia), Not Superscript/ Subscript
Formatted: Font: Not Bold, English (Malaysia), Not Superscript/ Subscript
Formatted: English (Malaysia), Not Superscript/ Subscript
Formatted: English (Malaysia), Not Superscript/ Subscript
Formatted: Font: Not Bold, English (Malaysia), Not Superscript/ Subscript
Formatted: English (Malaysia), Not Superscript/ Subscript

Formal analysis: J.J and I.S

Funding acquisition: N.S and S.J

Investigation: I.S., and E.M.H.

Methodology: J.J and I.S

Validation: S.J and S.I.I

Writing – original draft: I.S and JJ.

Writing – review & editing: S.J and S.I.I

References

1. Jiang W, Peng Y, Ye J, Wen Y, Liu G, Xie J. Effects of the entomopathogenic fungus *Metarhizium anisopliae* on the mortality and immune response of *Locusta migratoria*. Insects. 2020;11(1):1–12.
2. Sani I, Jamian S, Ismail SI, Saad N, Abdullah S, Hata EM, et al. Identification of entomopathogenic fungi *Metarhizium anisopliae* and *Purpureocillium lilacinum* from oil palm plantation soils in Universiti Putra Malaysia. Malays J Microbiol. 2022;18(1):105–12.
3. Liu F, Huang W, Wu K, Qiu Z, Huang Y, Ling E. Exploiting Innate Immunity for Biological Pest Control. In: Advances in Insect Physiology ~~[Internet]~~. Academic Press Inc.; 2017 ~~[cited 2020 Sep 7]~~. pp. 199–230. ~~Available from:~~ https://linkinghub.elsevier.com/retrieve/pii/S0065280617300012
4. González-Mas N, Sánchez-Ortiz A, Valverde-García P, Quesada-Moraga E. Effects of endophytic entomopathogenic ascomycetes on the life-history traits of *A~~a~~phis gossypii* glover and its interactions with melon plants. Insects. 2019;10(6):1–16.
5. Wei QY, Li YY, Xu C, Wu YX, Zhang YR, Liu H. Endophytic colonization by *Beauveria bassiana* increases the resistance of tomatoes against *Bemisia tabaci*.

Formatted: English (Malaysia), Not Superscript/ Subscript

Formatted: Font: Not Bold, English (Malaysia), Not Superscript/ Subscript

Formatted: English (Malaysia), Not Superscript/ Subscript

Formatted: Font: Not Bold, English (Malaysia), Not Superscript/ Subscript

Formatted: English (Malaysia), Not Superscript/ Subscript

Formatted: Font: Not Bold, English (Malaysia), Not Superscript/ Subscript

Formatted: English (Malaysia), Not Superscript/ Subscript

Formatted: Font: Not Bold, English (Malaysia), Not Superscript/ Subscript

Formatted: English (Malaysia), Not Superscript/ Subscript

Formatted: Font: Not Bold, English (Malaysia), Not Superscript/ Subscript

Formatted: English (Malaysia), Not Superscript/ Subscript

Formatted: Font: Not Bold, English (Malaysia), Not Superscript/ Subscript

Formatted: English (Malaysia), Not Superscript/ Subscript

Formatted: Font: Not Bold, English (Malaysia), Not Superscript/ Subscript

Formatted: Font: Not Superscript/ Subscript

Formatted: Font color: Auto

Field Code Changed

Formatted: Font: Not Bold, Italic, English (Malaysia), Not Superscript/ Subscript

Formatted: Font: Not Bold, Italic, English (Malaysia), Not Superscript/ Subscript

Formatted: Font: Not Bold, Italic, English (Malaysia), Not Superscript/ Subscript

Formatted: Font: Not Bold, Italic, English (Malaysia), Not Superscript/ Subscript

Formatted: Font: Not Bold, Italic, English (Malaysia), Not Superscript/ Subscript

Formatted: Font: Not Bold, Italic, English (Malaysia), Not Superscript/ Subscript

Formatted: Font: Not Bold, Italic, English (Malaysia), Not Superscript/ Subscript

Formatted: Font: Not Bold, Italic, English (Malaysia), Not Superscript/ Subscript

Arthropod Plant Interact ~~[Internet]~~. 2020;14(3):289–300. ~~Available from:~~ https://doi.org/10.1007/s11829-020-09746-9

6. Vega FE. Insect pathology and fungal endophytes. J Invertebr Pathol. 2008;98(3):277–9.

7. Allegrucci N, Velazquez MS, Russo ML, Perez E, Scorsetti AC. Endophytic colonisation of tomato by the entomopathogenic fungus *Beauveria bassiana:* The use of different inoculation techniques and their effects on the tomato leafminer *Tuta absoluta* (Lepidoptera: Gelechiidae). J Plant Prot Res. 2017;57(4):331–7.

8. Barra-Bucarei L, González MG, Iglesias AF, Aguayo GS, Peñalosa MG, Vera PV. *Beauveria bassiana* multifunction as an endophyte: Growth promotion and biologic control of *Trialeurodes vaporariorum*, (westwood) (hemiptera: Aleyrodidae) in tomato. Insects. 2020;11(9):1–15.

9. Nishi O, Sushida H, Higashi Y, Iida Y. Epiphytic and endophytic colonisation of tomato plants by the entomopathogenic fungus *Beauveria bassiana* strain GHA. Mycology ~~[Internet]~~. 2020;11:1–9. ~~Available from:~~ https://doi.org/10.1080/21501203.2019.1707723

10. Muvea AM, Meyhöfer R, Subramanian S, Poehling HM, Ekesi S, Maniania NK. Colonization of onions by endophytic fungi and their impacts on the biology of *T~~t~~hrips tabaci*. PLoS One. 2014;9(9):1–7.

11. Silva ACL, Silva GA, Abib PHN, Carolino AT, Samuels RI. Endophytic colonization of tomato plants by the entomopathogenic fungus *Beauveria bassiana* for controlling the South American tomato pinworm, *Tuta absoluta*. CABI Agric Biosci ~~[Internet]~~. 2020;1(1):1–9. ~~Available from:~~ https://doi.org/10.1186/s43170-020-00002-x

12. Lopez DC, Sword GA. The endophytic fungal entomopathogens *Beauveria bassiana* and *Purpureocillium lilacinum* enhance the growth of cultivated cotton (*Gossypium*

Formatted: Font: Not Bold, Italic, English (Malaysia), Not Superscript/ Subscript

Formatted: Font: Not Bold, Italic, English (Malaysia), Not Superscript/ Subscript

Formatted: Font: Not Bold, Italic, English (Malaysia), Not Superscript/ Subscript

Formatted: Font: Not Bold, Italic, English (Malaysia), Not Superscript/ Subscript

Formatted: Font: Not Bold, Italic, English (Malaysia), Not Superscript/ Subscript

Formatted: Font: Not Bold, Italic, English (Malaysia), Not Superscript/ Subscript

Formatted: Font: Not Bold, Italic, English (Malaysia), Not Superscript/ Subscript

Formatted: Font: Not Bold, Italic, English (Malaysia), Not Superscript/ Subscript

Formatted: Font: Not Bold, Italic, English (Malaysia), Not Superscript/ Subscript

Formatted: Font: Not Bold, Italic, English (Malaysia), Not Superscript/ Subscript

Formatted: Font: Not Bold, Italic, English (Malaysia), Not Superscript/ Subscript

Formatted: Font: Not Bold, Italic, English (Malaysia), Not Superscript/ Subscript

Formatted: Font: Not Bold, Italic, English (Malaysia), Not Superscript/ Subscript

Formatted: Font: Not Bold, Italic, English (Malaysia), Not Superscript/ Subscript

hirsutum) and negatively affect survival of the cotton bollworm (Helicoverpa zea). Biol Control ~~[Internet]~~. 2015;89:53–60. ~~Available from:~~ http://dx.doi.org/10.1016/j.biocontrol.2015.03.010

13. Vega FE. The use of fungal entomopathogens as endophytes in biological control: a review. Mycologia. 2018;110(1):4–30.
14. Garrido-Jurado, I., Resquı´n-Romero, G., Amarilla, S.P., Rı´os-Moreno, A., Carrasco, L., Quesada-Moraga E. Transient endophytic colonization of melon plants by entomopathogenic fungi after foliar application for the control of *Bemisia tabaci* Gennadius (Hemiptera: Aleyrodidae). J Pest Sci (2004). 2017;90:319–30.
15. Mantzoukas S, Eliopoulos PA. Endophytic entomopathogenic fungi: A valuable biological control tool against plant pests. Appl Sci. 2020;10(1):1–13.
16. Hamzah TNT, Lee SY, Hidayat A, Terhem R, Faridah-Hanum I, Mohamed R. Diversity and characterization of endophytic fungi isolated from the tropical mangrove species, *Rhizophora mucronata*, and identification of potential antagonists against the soil-borne fungus, *Fusarium solani*. Front Microbiol. 2018;9(01707):1–17.
17. Siddiquee S, Yusuf UK, Zainudin NAIM. Morphological and molecular detection of *Fusarium chlamydosporum* from root endophytes of *Dendrobium crumenatum*. African J Biotechnol. 2010;9(26):4081–90.
18. Saad KA, Roff MNM, Hallett RH, Idris AB. Aphid-induced Defences in Chilli Affect Preferences of the Whitefly, *Bemisia tabaci* (Hemiptera: Aleyrodidae). Sci Rep ~~[Internet]~~. 2015;5(13697):1–9. ~~Available from:~~ http://dx.doi.org/10.1038/srep13697
19. Sani, I., Ismail, S.I., Saad, N., Abdullah, S., Jalinas, J., Jamian S. Insect Pests of Vegetables in Malaysia and their Management using Entomopathogenic Fungi. Serangga. 2020;25(3):126–43.
20. Frohlich DR, Torres-Jerez I, Bedford ID, Markham PG, Brown JK. A

Formatted: Font: Not Bold, Italic, English (Malaysia), Not Superscript/ Subscript

Formatted: Font: Not Bold, Italic, English (Malaysia), Not Superscript/ Subscript

Formatted: Font: Not Bold, Italic, English (Malaysia), Not Superscript/ Subscript

Formatted: Font: Not Bold, Italic, English (Malaysia), Not Superscript/ Subscript

Formatted: Font: Not Bold, Italic, English (Malaysia), Not Superscript/ Subscript

Formatted: Font: Not Bold, Italic, English (Malaysia), Not Superscript/ Subscript

Formatted: Font: Not Bold, Italic, English (Malaysia), Not Superscript/ Subscript

Formatted: Font: Not Bold, Italic, English (Malaysia), Not Superscript/ Subscript

Formatted: Font: Not Bold, Italic, English (Malaysia), Not Superscript/ Subscript

Formatted: Font: Not Bold, Italic, English (Malaysia), Not Superscript/ Subscript

Formatted: Font: Not Bold, Italic, English (Malaysia), Not Superscript/ Subscript

Formatted: Font: Not Bold, Italic, English (Malaysia), Not Superscript/ Subscript

Formatted: Font: Not Bold, Italic, English (Malaysia), Not Superscript/ Subscript

phylogeographical analysis of the *Bemisia tabaci* species complex based on mitochondrial DNA markers. Mol Ecol [Internet]. 1999 [cited 2022 Mar 11];8(10):1683–91. Available from: https://pubmed.ncbi.nlm.nih.gov/10583831/

21. Oliveira DGP, Pauli G, Mascarin GM, Delalibera I. A protocol for determination of conidial viability of the fungal entomopathogens *Beauveria bassiana* and *Metarhizium anisopliae* from commercial products. J Microbiol Methods. 2015;119:44–52.

22. Tefera T, Vidal S. Effect of inoculation method and plant growth medium on endophytic colonization of sorghum by the entomopathogenic fungus *Beauveria bassiana*. BioControl. 2009;54(5):663–9.

23. Bamisile BS, Senyo Akutse K, Dash CK, Qasim M, Ramos Aguila LC, Ashraf HJ, et al. Effects of Seedling Age on Colonization Patterns of Citrus limon Plants by Endophytic *Beauveria bassiana* and *Metarhizium anisopliae* and Their Influence on Seedlings Growth. J Fungi. 2020;6(1):29.

24. Canassa F, Tall S, Moral RA, Lara IAR d., Delalibera I, Meyling N V. Effects of bean seed treatment by the entomopathogenic fungi *Metarhizium robertsii* and *Beauveria bassiana* on plant growth, spider mite populations and behavior of predatory mites. Biol Control. 2019;132:199–208.

25. Bocco R, Lee M, Kim D, Ahn S, Park JW, Lee SY, et al. Endophytic isaria javanica pf185 persists after spraying and controls *Mmyzus persicae* (Hemiptera: Aphididae) and *Ccolletotrichum acutatum* (glomerellales: Glomerellaceae) in pepper. Insects. 2021;12(7):1–18.

26. Sun T, Shen Z, Shaukat M, Du C, Ali S. Endophytic isolates of *Cordyceps fumosorosea* to enhance the growth of *Solanum melongena* and reduce the survival of whitefly (*Bemisia tabaci*). Insects. 2020;11(2):8–10.

27. Mantzoukas S, Chondrogiannis C, Grammatikopoulos G. Effects of three endophytic

Formatted: Font: Not Bold, Italic, English (Malaysia), Not Superscript/ Subscript

Formatted: Font: Not Bold, Italic, English (Malaysia), Not Superscript/ Subscript

Formatted: Font: Not Bold, Italic, English (Malaysia), Not Superscript/ Subscript

Formatted: Font: Not Bold, Italic, English (Malaysia), Not Superscript/ Subscript

Formatted: Font: Not Bold, Italic, English (Malaysia), Not Superscript/ Subscript

Formatted: Font: Not Bold, Italic, English (Malaysia), Not Superscript/ Subscript

Formatted: Font: Not Bold, Italic, English (Malaysia), Not Superscript/ Subscript

Formatted: Font: Not Bold, Italic, English (Malaysia), Not Superscript/ Subscript

Formatted: Font: Not Bold, Italic, English (Malaysia), Not Superscript/ Subscript

Formatted: Font: Not Bold, Italic, English (Malaysia), Not Superscript/ Subscript

Formatted: Font: Not Bold, Italic, English (Malaysia), Not Superscript/ Subscript

Formatted: Font: Not Bold, Italic, English (Malaysia), Not Superscript/ Subscript

Formatted: Font: Not Bold, Italic, English (Malaysia), Not Superscript/ Subscript

Formatted: Font: Not Bold, Italic, English (Malaysia), Not Superscript/ Subscript

Formatted: Font: Not Bold, Italic, English (Malaysia), Not Superscript/ Subscript

Formatted: Font: Not Bold, Italic, English (Malaysia), Not Superscript/ Subscript

Formatted: Font: Not Bold, Italic, English (Malaysia), Not Superscript/ Subscript

Formatted: Font: Not Bold, Italic, English (Malaysia), Not Superscript/ Subscript

Formatted: Font: Not Bold, Italic, English (Malaysia), Not Superscript/ Subscript

Formatted: Font: Not Bold, Italic, English (Malaysia), Not Superscript/ Subscript

entomopathogens on sweet sorghum and on the larvae of the stalk borer *Sesamia nonagrioides*. Entomol Exp Appl. 2015;154(1):78–87.

28. Lopez DC, Zhu-Salzman K, Ek-Ramos MJ, Sword GA. The entomopathogenic fungal endophytes *Purpureocillium lilacinum* (formerly *Paecilomyces lilacinus*) and Beauveria bassiana negatively affect cotton aphid reproduction under both greenhouse and field conditions. PLoS One. 2014;9(8):1–8.

29. Elena GJ, Beatriz PJ, Alejandro P, Roberto LE. *Metarhizium anisopliae* (Metschnikoff) Sorokin promotes growth and has endophytic activity in tomato plants. Adv Biol Res (Rennes). 2011;5(1):22–7.

30. Rasool S, Vidkjær NH, Hooshmand K, Jensen B, Fomsgaard IS, Meyling N V. Seed inoculations with entomopathogenic fungi affect aphid populations coinciding with modulation of plant secondary metabolite profiles across plant families. New Phytol. 2021;229(3):1715–27.

Formatted: Font: Not Bold, Italic, English (Malaysia), Not Superscript/ Subscript

Formatted: Font: Not Bold, Italic, English (Malaysia), Not Superscript/ Subscript

Formatted: Font: Not Bold, Italic, English (Malaysia), Not Superscript/ Subscript

Formatted: Font: Not Bold, Italic, English (Malaysia), Not Superscript/ Subscript

Formatted: Font: Not Bold, Italic, English (Malaysia), Not Superscript/ Subscript

Formatted: Indent: Left: 0", First line: 0", Line spacing: Double

Response to Reviewers 1 Comments

(Manuscript Number: PONE-D-22-19772)

The authors are very much thankful to the reviewers for their meticulous review and valuable comments. We have now incorporated all the two reviewers' comments and suggestions to improve the manuscript. Details of our response to each comment, corrections and suggestions are given below along with the track lines in the manuscript in the manuscript that was first/originally submitted.

Abstract

Point 1: d

Response 1: "d" has been added in the word demonstrate

Point 2: ","

Response 2: comma "," has been added

Point 3: ","

Response 3: comma "," has been added

Point 4: s

Response 4: "s" has added in the word "endophyte"

Point 5: s

Response 5: "s" has been placed as recommended

Point 6: of what?

Response 6: "of the population of whitefly". The statement has been modified by replacing population with mortality

Point 7: from plant tissues?

Response 7: Yes, from plant tissues (root, stem and leaf), the statement has been modified

Point 6: *B. tabaci* on seedlings. So caused mortality? Be more direct!

Response 6: the statement has been modified as can be seen in the manuscript at line 31 -32

Point 6: control % mortality is a bit high??

Response 6: Although we have more than 10% mortality in the control treatments, the data were not corrected as suggested by Schneider-Orelli's (1947) formula, because there is significant different between fungal colonized seedlings and those of untreated control seedlings.

Point 6:??

Response 6: means number = mean (average) values recorded.

Point 6: seems unlikly and not the most important result. State the important finding not the geographical novelty!

Response 6: The statement has been modified as can be seen from the manuscript a t line 35-38

Point 6: Normally good to avoid keywords that are already in the title.

Response 6: keywords has been corrected as recommended

Point 6: e

Response 6: endophytic has been changed to endophyte

Introduction

Point 1: inundative

Response 1: inundated has been corrected to "inundative"

Point 2: seem unsure of that. They are sprayed of course!

Response 2: the statement has been modified as "Several EPF have been commercialized for sap-sucking pests control" and affected in the manuscript at line 51-52

Point 3: better to say plants infested with pests.

Response 3: the statement has been changed to “plants infested with pests".

Point 4: specifically about B. tabaci and tomato plants, so maybe you should say that here!

Response 4: "including *B. tabaci* on tomato plants" has been added as recommended

Point 5: have also been

Response 5: are has been replaced with "have also been" as can be seen in the manuscript at line 58

Point 6: s

Response 6: s has been added in the word pest

Point 7: causing

Response 7: "with" has been replaced with causing

Point 8: ","

Response 8: "," has been added after the word tissues

Point 9: al

Response 9: fungi has been changed to fungal

Point 10: just several days??

Response 10: several days has been changed to several month

Point 11: ","

Response 11: comma "," has been added after the word injection

Point 12: Is the geographical aspect that important to state here?

Response 12: Yes. This is to show the first report of EPF as endophytes and promoting growth plants in Malaysia.

Point 13: what does that mean? Explain!

Response 13: The ability to protect tomato plant from insect infestation when EPF colonized tomato artificially with various inoculation methods

Point 14: REF!!

Response 14: References has been cited as can be seen in the manuscript at line 80

Point 15: in

Response 15: "of" has been replaced with in

Point 16: plants?

Response 16: Crops has been replaced with plants

Point 17: management

Response 17: biological control has been replaced IPM

Methods

Point 1: dont know what that means??

Response 1: MT1 is the name of tomato seed variety in Malaysia.

Point 2: was

Response 2: "were" was changed to was

Point 2: ??

Response 2: UPM is the abbreviation of Universiti Putra Malaysia. The full meaning has know been written before the abbreviation

Point 2: more than 24h in a day?

Response 2: corrected (24h (12L: 12D)) as recommended

Point 2: what does that mean?

Response 2: We mean tomato seedlings that damage during rearing of *B. tabaci*

Point 2: The two

Response 2: corrected as recommended

Point 8: more details please!

Response 8: More explanation about Fungal isolates used in the study has been provided as can be seen in the manuscript at line.......

Point: as shown

Response: "can be seen has been replaced with "are shown"

Point: Table S1

Response: corrected as recommended and affected in the manuscript at line 104

Point: were

Response: "are" has been replaced with were

Point: an

Response: the has been replaced with an

Point: Sorgum paper??

Response: Yes, we followed the methods used in the paper. However, other reference from tomato papers has been found and included.

Point: I found this section very confusing. No information on the evaluation methods for mortality etc.

Response: The statement/subheading has been changed by replacing population with mortality. The reduction in the population is what we referred to as mortality.

Point: not really...kill..is better!

Response: effect has been replaced with kill

Point: Just one?

Response: whitefly has been corrected to be in plural "whiteflies"

Point: Why one then about 5 days later another 20-25 adults??

Response: whitefly has been corrected to be in plural "whiteflies" as can be seen in the above at line..........

Point: Dead or alive? What happened to the dead ones..dropped off the plant? No details to understand the "reduction in the population"??

Response: The statement has been changed as "The populations of *B. tabaci* were estimated by counting the number of dead adults dropped off the plant on each cage after 7 days.

Point: confusing!! Do you mean that females had laid eggs on the plants, which became nymphs, which then became adults?? The reductiion in the number of adults was of the F2 population then? Do adults emerge? Whiteflys are hemimetabolous insects!

Response: The statement has been corrected as be seen in the manuscript at line 189 - 194

Results

Point 1: of

Response 1: "in" has been replaced with "of"

Point 2: both of

Response 2: "all" have been replaced with "both of"

Point 3: s

Response 3: "s" has been added in the word "endophyte"

Point 3: was

Response 3: "was" has been added between *P. lilacinum* and re-isolated

Point 4: was

Response 4: "is" was replaced with was

Point 5: at

Response 5: "in" was replaced with "at"

Point 6: ed

Response 6: "ed" was added in the word differ

Point 7: explain better, confusing

Response 7: the statement has been explain better and affected at manuscript at line 216 - 218

Point 8: showed?

Response 8: expressed has been replaced with "showed"

Point 9: s

Response 9: "s" has been added in the word effect

Point 10: number of leaves

Response 10: Yes, number of leaves. leaf has been changed to leaves"

Point 11: no numbers here in the text!!

Response 11: the mean numbers of leaves of the tomato seedlings treatments has been included

Point 12: no numbers??

Response 12: the numbers has been included as recommended and affected in the manuscript at line 238-240

Point 13: s

Response 13: s has been places after shoot

Point 14: why however?

Response 14: However has been removed

Point 15: of what?

Response 15: The statement/subheading has been changed to "Effects of Endophytically-Colonized tomato seedlings on the Mortality, and adult emergence of *B. tabaci*"

Point 16: how did the plants reduce the population?

Response 16: We mean endophytic colonized plants, not plants alone. However, the statement has been rephrased as can be seen in the manuscript at line 258-259

Point 17: when

Response 17: "as" has been replaced with when

Point 18: of

Response 18: in has been replaced with of

Point 19: as I said in the abstract, this control data is strange, I guess you mean mortality in the control treatments was 19%, whilst in treated the mortality was 51% and 45%.

Response 19: the fact that we have 19% mortality in the control treatments, our analysis of variance shows significant different between fungal colonized seedlings and those of untreated control seedlings.

Point 20: dont understand this experiment at all. Adults emerging from where??

Response 20: Adult emergence from the first generation. However, the statement has been corrected as can be seen in the manuscript at line 270-274

Discussion

Point 1: in

Response 1: on has been changed with in

Point 2: ???? economically important plants??

Response 2: Yes, economically important plants. the statement has been corrected.

Point 3: in

Response 3: on has been changed to in

Point 4: period

Response 4: the word "day" has been changed to "period"

Point 5: period

Response 5: the word "day" has been changed with "period"

Point 6: lowest

Response 6: the word "lowest" has been changed with "least"

Point 7: age

Response 7: "age" has been added in the word percent

Point 8: lowest

Response 8: the word "least" has been changed to "lowest"

Point 9: or

Response 9: "and" has been replaced to "or"

Point 10: d

Response 10: promotes has been changed to promoted

Point 11: ,

Response 11: comma "," has been placed after length

Point 12: on what? pests??

Response 12: "negative effect of endophytic EPF on *B. tabaci*. The statement has been corrected"

Point 13: confusing

Response 13: The statement has been corrected and affected in the manuscript at line 317 and 318

Conclusion

Point 1: Is that really an important conclusion, because it is the first study in Malaysia or other neighboring countries (which ones...all of them??)

Response 1: Yes. This is to show the first report of EPF as entophytes and promoting growth plants in Malaysia and some neighboring country

Point 2: or

Response 2: and has been replaced with or

Point 3: both

Response 3: "all the two" has been replaced with both

Point 4: and

Response 4: were has been placed between "and" and "recovered"

Point 5: were

Response 5: were has been placed between "and" and "recovered"

Point 6: plants

Response 6: plants has been placed between "the and "treated"

Point 7: with

Response 7: with has been placed between "treated" and "EPF"

Point 8: of

Response 8: in has been replaced with of

Point 1:

Response 3:

Point 1:

Response 3:

Supporting Information

Point 1 s

Response 2: suspension has been changed to suspensions

Point 2: why is the fungus sporulating inside the plant?

Nothing in the results about this!!

Response 2: Endophytic colonization of both isolates (Cjc-03 and TS-01) in tomato plants was also examined using live imaging by CLSM. A noticeable spore, germination of conidia and appressorium formation observed indicated that the spores are alive on the tomato plant tissues (S4 Fig. 1).

Point 3: in

Response 3: corrected as recommended

Figures

Point 1: s

Response 3: "s" has been added

Point 1: s

Response 3: "s" has been added

Point 1: and evaluated

Response 3: corrected as recommended

Point 1: times after inoculation

Response 3: corrected as recommended

Point 1: leaf

Response 3: corrected as recommended

Point 1: Not part of Fig. 1

Response 3: The statement has been removed as it was part of the supporting statement

Point 1: height isnt better?

Response 3: corrected as recommended

Point 1: Never seen a tomato seedling with 80 leaves!!

Response 3: Leaves number has been corrected as leaflets number, because the mean number of leaflets of each seedling was recorded.

The statement has been changed to "number of leaves (leaflets)"

Point 1: mean numbers of leaves has got to be wrong! Impossible to have 80 leaves. Seems to be mixed up with % of something?

Response 3: Leaves number has been corrected as leaflets number, because the mean number of leaflets of each seedling was recorded.

The statement has been changed to "number of leaves (leaflets)"

Point 1: length from root tip to stem tip??

Response 3: all the shoot part of the plant

Point 1: treated

Response 3: corrected as recommended

Point 1: 2

Response 3: corrected as recommended

Point 1: 2

Response 3: corrected as recommended

Response to Reviewers 2 Comments

(Manuscript Number: PONE-D-22-19772)

The authors are very much thankful to the reviewers for their meticulous review and valuable comments. We have now incorporated all the two reviewers' comments and suggestions to improve the manuscript. Details of our response to each comment, corrections and suggestions are given below along with the track lines in the manuscript in the manuscript that was first/originally submitted.

Abstract

Point 1: fungal entomopathogens

Response 1: The statement has been modified.

Point 2: ","

Response 2: comma "," has been added

Point 3: ","

Response 3: comma "," has been added

Point 4: s

Response 4: "s" has added in the word "endophyte"

Point 5: s

Response 5: "s" has been placed as recommended

Introduction

Response 1: there is no any comment made by reviewer

Methods

Point 1: released

Response 1: placed has been replaced with released

Point 2: please describe the observation of adult emergence from nymphs were taken. How many nymphs were survived.

Response 2: The eggs and nymphs stages were monitored over a period of 35 days but only data of adult's emergence were recorded. However, the statement has been modified and affected in the manuscript at line 193 -198

Point 2: Cjc??? please check

Response 2: name of the isolates "Cjc-03" has been checked and corrected throughout the manuscript

Results

Point 1: please check

Response 1: name of the isolates "Cjc-03" has been checked and corrected throughout the manuscript

Point 2:

Response 2: name of the isolates "Cjc-03" has been checked and corrected throughout the manuscript

Point 3: Cjc??? please check

Response 3: corrected as recommended

Discussion

Point 1: economically important plants

Response 1: corrected as recommended

Conclusion

Response 1: there is no any comment made by reviewer

Supporting Information

Response 1: there is no any comment made by reviewer

Reviewer #1: General comments

Point 1: The paper is not acceptable in present form. Title: Inoculation and Colonization of Entomopathogenic Fungi, 2 Cordyceps javanica and Purpureocillium lilacinum in 3 Tomato Plants, and their effect on Seedlings Growth, 4 Population, and adult Emergence of Whitefly, Bemisia 5 tabaci (Gennadius)

Response 1: The authors are very much thankful to the reviewers for their meticulous review and valuable comments. We have now incorporated all the two reviewers' comments and suggestions to improve the manuscript.

Point 2: The authors should give full names of Cordyceps javanica e.g. should be written as when it appears for the first time and in title e.g. Cordyceps javanica (wf GA17) Purpureocillium lilacinum should be written as Purpureocillium lilacinum (Thom) Luangsa-ard, Hou- braken, Hywel-Jones & Samson (2011) when it appears first time and in the title. There is no problem about control treatments used in the experiment. Thereafter, the two names could be written as C. javanica and P. lilacinum in the annotated text of the paper. There is need to give more information.

Response 2: The full name of *Isaria javanica* and *Purpureocillium lilacinum* "(*Isaria javanica* (Frieder. & Bally) Samson & Hywel-jone 2005 *and Purpureocillium lilacinum* (Thom) Luangsa-ard, Hou- braken, Hywel-Jones & Samson (2011)") has been written first in the abstract as recommended by reviewer. However, writing two species in full in the title will make it too long which could make readers difficult to digest, inducing the reader with little time and commitment to move on to the next article in his or search.

Point 3: The topic is very interesting. Therefore present abstract should be rewritten to give more details about the experiment.

Response 3: the abstract has been modified as recommended by reviewer

Point 4: The authors should give more information about the induced conclusions based on the experimental results.

Response 4: Corrected as recommended

Point 5: Keywords: All words shown in the title must not be shown in the keywords. İs very long. It should be shortened

Response 5: Corrected as recommended

Point 6: authors should not use the language of first or second person. All paper should be written in the language of third person.

Response 6: Corrected as recommended

Reviewer #2: General comments

Point 1: This is an interesting study of the endophytic colonization of tomato plants with two of the less studied species of entomopathogenic fungi. Fungal inoculation was performed by treating the seeds with conidia suspensions. The subsequently inoculated seeds were allowed to germinate and various aspects of the plants were then studies: colonization of different parts of the plants from day 15 to day 60 post inoculation; plant development: seedling height/length, number of leaves, fresh and dry weight of roots, shoots and leaves. Furthermore, the authors also looked at some aspects of the interaction of EFP colonized plants with an important tomato pest, *B. tabaci*. In that case the authors looked at mortality of

B. tabaci adults placed on the plants and an adult emergence test, which I could not understand at all.

Response 1: The authors are very much thankful to the reviewers for their meticulous review and valuable comments. We have now incorporated all the two reviewers' comments and suggestions to improve the manuscript.

Point 2: The results showed the highly positive effects of both fungal isolates on plant development (which has been shown many times before for other EPF endophytes), however, the increased leaf number data is really odd.

Response 2: endophytic fungal isolates (Cjc-03 and TS-01) enhanced the growth of inoculated tomato seedlings, as there were significant differences between fungal treated seedlings and the untreated control seedlings with respect to the average height of seedlings and number of leaves at different weeks post-inoculation.

Point 3; The colonization of different parts of the plants was variable, with the highest % in leaves, followed by stems and a low level of colonization in roots. The peak colonization was seen at 30 days, falling rapidly at 60 days for leaves. The pattern for stem colonization was different to that of leaves, with the highest levels at 15 days, dropping off at 30 days. Importantly, the authors used confocal microscopy to look at plant colonization. However, they give little attention to these results and put the images in the supporting material. In fact, the data for microscopy needs explaining as it is rather confusing, but maybe it is worth exploring in more detail as very few endophyte studies have used this technique.

Response 3: the data of using confocal microscopy for Endophytic colonization of both isolates (Cjc-03 and TS-01) in tomato plants has been included in the manuscript and effected at line 226-229

Point 4: The data for insect-plant interactions is also very confusing, with very little

explanation of the M&M or results. I have made some corrections to the PDF file, but the English needs a general overhaul.

Response 4: We have incorporated all the comments and suggestions to improve the manuscript.

Point 5: Modify the title as very confusing!

Response 5: the title has been modified

Reviewer #3: General comments

Point 1: The hypothesis of the work is wonderful. Eventhough, some minor corrections/typos needs to be corrected. However, it is not clear that how the author has conducted the mortality experiments? How the experiment was conducted? the the adults were relased and observed? Simultaneously how the emergence observation were recorded. This methodology part needs to be described well so as to further understand the esults and conclusion.

Response 1: The authors are very much thankful to the reviewers for their meticulous review and valuable comments. We have now incorporated all the two reviewers' comments and suggestions to improve the manuscript.
