# Supplementary material for: Inoculation and colonization of the entomopathogenic fungi, Isaria javanica and Purpureocillium lilacinum, in tomato plants, and their effect on seedling growth, mortality and adult emergence of Bemisia tabaci (Gennadius)
Source: PLoS One. 2023 May 22;18(5):e0285666. doi: 10.1371/journal.pone.0285666 (PMC10202273; doi:10.1371/journal.pone.0285666)
Supplement: Attachment — Submitted filename: PONE-D-22-19772_R2_reviewer really last chance! 04 03 23.pdf [file pone.0285666.s012.pdf]

PLOS ONE

Inoculation and colonization of the entomopathogenic fungi, Isaria javanica and Purpureocillium lilacinum, in tomato plants, and their effect on seedling growth, mortality and adult emergence of Bemisia tabaci (Gennadius)

--Manuscript Draft--

**Manuscript Number:**	PONE-D-22-19772R2
**Article Type:**	Research Article
**Full Title:**	Inoculation and colonization of the entomopathogenic fungi, Isaria javanica and Purpureocillium lilacinum, in tomato plants, and their effect on seedling growth, mortality and adult emergence of Bemisia tabaci (Gennadius)
**Short Title:**	Endophytic entopathogenic fungi Promote growth of tomato and negatively affected Whitefly, B. tabaci
**Corresponding Author:**	Syari Jamian, Ph.D<br>UPM - Serdang: Universiti Putra Malaysia<br>Serdang, Selangor MALAYSIA
**Keywords:**	Keywords: biocontrol, endophyte, mortality, plant growth,
**Abstract:**	Entomopathogenic fungi (EPF) are natural enemies present in the insect population and have long been recognized as biological control agents against many insect pests. Some isolates have also been established as endophytes, benefiting their host plants without causing any symptoms or negative effects. Here we demonstrated two fungal species, Isaria javanica (Frieder. & Bally) Samson & Hywel-jone 2005 and Purpureocillium lilacinum (Thom) Luangsa-ard, Hou- braken, Hywel-Jones & Samson (2011) as an endophytes in tomato plants by using the seed inoculation method and examining their effect on plant growth, mortality, and adult emergence of B. tabaci.Our study indicated that tomato seeds treated with a fungal suspensions of I. javanica and P. lilacinum allowed their recovery from plant tissues (root, stem and leaf) up to 60 days after inoculation (DAI). Both endophytic isolates also significantly caused mortality of adult B. tabaci on seedlings inoculated with, I. javanica (51.92±4.78%), and P. lilacinum (45.32±0.20%) compared to the control treatment (19.29±2.35). However, the adult emergence mean number was significantly high in the control treatments (57.50±2.66) compared to I. javanica (15.00±1.47) and P. lilacinum (28.75±4.78) treatments.This study provides evidence that native endophytic isolates of I. javanica and P. lilacinum have a biocontrol potentials on whiteflies and could explored as plant growth promoters.
**Order of Authors:**	Ibrahim Sani, PhD
	Syari Jamian, Ph.D
	Norsazilawati Saad, Ph.D
	Sumaiyah Abdullah, Ph.D
	Erneeza Mohd Hata
	Johari Jalinas, Ph.D
	Siti Izera Ismail, Ph.D
**Response to Reviewers:**	The authors are very much thankful to the reviewers for their meticulous review and valuable comments. We have now incorporated all the two reviewers' comments and suggestions to improve the manuscript.
**Additional Information:**	
**Question**	**Response**
**Financial Disclosure**<br><br>Enter a financial disclosure statement that describes the sources of funding for the	This study was funded by the Fundamental Research Grant Scheme (FRGS) from the Ministry of Higher Education Malaysia (Grant no. 5540212; reference code FRGS/1/2019/WAB01/UPM/02/36). he funders had no role in study design, data collection and analysis, decision to publish, or preparation of the manuscript.

Powered by Editorial Manager® and ProduXion Manager® from Aries Systems Corporation

work included in this submission. Review the submission guidelines for detailed requirements. View published research articles from *PLOS ONE* for specific examples.<br><br>This statement is required for submission and **will appear in the published article** if the submission is accepted. Please make sure it is accurate.<br><br>**Unfunded studies**<br>Enter: *The author(s) received no specific funding for this work.*<br><br>**Funded studies**<br>Enter a statement with the following details:<br>• Initials of the authors who received each award<br>• Grant numbers awarded to each author<br>• The full name of each funder<br>• URL of each funder website<br>• Did the sponsors or funders play any role in the study design, data collection and analysis, decision to publish, or preparation of the manuscript?<br>• **NO** - Include this sentence at the end of your statement: *The funders had no role in study design, data collection and analysis, decision to publish, or preparation of the manuscript.*<br>• **YES** - Specify the role(s) played.<br><br>* typeset	
**Competing Interests**<br><br>Use the instructions below to enter a competing interest statement for this submission. On behalf of all authors, disclose any competing interests that could be perceived to bias this work—acknowledging all financial support and any other relevant financial or non-financial competing interests.<br><br>This statement is required for submission and **will appear in the published article** if the submission is accepted. Please make sure it is accurate and that any funding sources listed in your Funding Information	The authors have declared that no competing interests exist.

later in the submission form are also declared in your Financial Disclosure statement.<br><br>View published research articles from *PLOS ONE* for specific examples.<br><br>**NO authors have competing interests**<br><br>Enter: *The authors have declared that no competing interests exist.*<br><br>**Authors with competing interests**<br><br>Enter competing interest details beginning with this statement:<br><br>*I have read the journal's policy and the authors of this manuscript have the following competing interests: [insert competing interests here]*<br><br>* typeset	
**Ethics Statement**<br><br>Enter an ethics statement for this submission. This statement is required if the study involved:<br><br>• Human participants<br>• Human specimens or tissue<br>• Vertebrate animals or cephalopods<br>• Vertebrate embryos or tissues<br>• Field research<br><br>Write "N/A" if the submission does not require an ethics statement.<br><br>General guidance is provided below. Consult the submission guidelines for detailed instructions. **Make sure that all information entered here is included in the Methods section of the manuscript.**	N/A

**Format for specific study types**<br><br>**Human Subject Research (involving human participants and/or tissue)**<br>• Give the name of the institutional review board or ethics committee that approved the study<br>• Include the approval number and/or a statement indicating approval of this research<br>• Indicate the form of consent obtained (written/oral) or the reason that consent was not obtained (e.g. the data were analyzed anonymously)<br><br>**Animal Research (involving vertebrate animals, embryos or tissues)**<br>• Provide the name of the Institutional Animal Care and Use Committee (IACUC) or other relevant ethics board that reviewed the study protocol, and indicate whether they approved this research or granted a formal waiver of ethical approval<br>• Include an approval number if one was obtained<br>• If the study involved *non-human primates*, add *additional details* about animal welfare and steps taken to ameliorate suffering<br>• If anesthesia, euthanasia, or any kind of animal sacrifice is part of the study, include briefly which substances and/or methods were applied<br><br>**Field Research**<br><br>Include the following details if this study involves the collection of plant, animal, or other materials from a natural setting:<br>• Field permit number<br>• Name of the institution or relevant body that granted permission	
**Data Availability**<br><br>Authors are required to make all data underlying the findings described fully available, without restriction, and from the time of publication. PLOS allows rare exceptions to address legal and ethical concerns. See the PLOS Data Policy and FAQ for detailed information.	Yes - all data are fully available without restriction

A Data Availability Statement describing where the data can be found is required at submission. Your answers to this question constitute the Data Availability Statement and **will be published in the article**, if accepted.<br><br>**Important:** Stating 'data available on request from the author' is not sufficient. If your data are only available upon request, select 'No' for the first question and explain your exceptional situation in the text box.<br><br>Do the authors confirm that all data underlying the findings described in their manuscript are fully available without restriction?	
**Describe where the data may be found in full sentences. If you are copying our sample text, replace any instances of XXX with the appropriate details.**<br><br>• If the data are **held or will be held in a public repository**, include URLs, accession numbers or DOIs. If this information will only be available after acceptance, indicate this by ticking the box below. For example: *All XXX files are available from the XXX database (accession number(s) XXX, XXX.).*<br>• If the data are all contained **within the manuscript and/or Supporting Information files**, enter the following: *All relevant data are within the manuscript and its Supporting Information files.*<br>• If neither of these applies but you are able to provide **details of access elsewhere**, with or without limitations, please do so. For example:<br><br>*Data cannot be shared publicly because of [XXX]. Data are available from the XXX Institutional Data Access / Ethics Committee (contact via XXX) for researchers who meet the criteria for access to confidential data.*<br><br>*The data underlying the results presented in the study are available from (include the name of the third party*	All relevant data are within the manuscript and its Supporting Information files.

and contact information or URL).<br>• This text is appropriate if the data are owned by a third party and authors do not have permission to share the data.<br><br>* typeset	
Additional data availability information:	

Powered by Editorial Manager® and ProduXion Manager® from Aries Systems Corporation

Manuscript

Click here to access/download;Manuscript;Revised Manuscript_2_ without track changes_2.docx

Inoculation and colonization of the entomopathogenic fungi, *Isaria javanica* and *Purpureocillium lilacinum*, in tomato plants, and their effect on seedling growth, mortality and adult emergence of Bemisia *tabaci* (Gennadius)

Ibrahim Sani[1,2], Syari Jamian[1,3*], Norsazilawati Saad[1], Sumaiyah Abdullah[1,]Erneeza Mohd Hata[4], Johari Jalinas[5] and Siti Izera Ismail[1,3*]

[1]Department of Plant Protection, Faculty of Agriculture, Universiti Putra Malaysia, Serdang 43400, Malaysia

[2] Department of Biology, Faculty of Natural and Applied Sciences, Umaru Musa Yar'adua University, P.M.B., Katsina 2218, Nigeria

[3] Laboratory of Climate-Smart Food Crop Production, Institute of Tropical Agriculture and Food Security (ITAFoS), Universiti Putra Malaysia, Serdang 43400, Malaysia

[4]Laboratory of Sustainable Agronomy & Crop Protection, Institute of Plantation Studies, Universiti Putra Malaysia, 43400 UPM Serdang, Selangor

[5] Centre for Insect Systematics, Department of Biological Sciences and Biotechnology, Faculty of Science & Technology, Universiti Kebangsaan Malaysia, Bangi 43600, Malaysia

syari@upm.edu.my (SJ);izera@upm.edu.my (SII)

Abstract

Entomopathogenic fungi (EPF) are natural enemies present in the insect population and have long been recognized as biological control agents against many insect pests. Some isolates have also been established as endophytes, benefiting their host plants without causing any symptoms or negative effects. Here we demonstrated two fungal species, *Isaria javanica* (Frieder. & Bally) Samson & Hywel-jone 2005 and *Purpureocillium lilacinum* (Thom) Luangsa-ard, Hou- braken, Hywel-Jones & Samson (2011) as ~~an~~ endophytes in tomato plants by using the seed inoculation method and examining their effect on plant growth, mortality, and adult emergence ~~of *B. tabaci*~~. Our study indicated that tomato seeds treated with a fungal suspension of *I. javanica* and *P. lilacinum* allowed their recovery from plant tissues (root, stem and leaf) up to 60 days after inoculation (DAI). Both endophytic isolates also ~~significantly~~ caused mortality of adult *B. tabaci* on seedlings inoculated with, *I. javanica* (51.92±4.78%), and *P. lilacinum* (45.32±0.20%) compared to the control treatment (19.29±2.35). ~~However, the~~ adult emergence mean number was significantly high in the control treatments (57.50±2.66) compared to *I. javanica* (15.00±1.47) and *P. lilacinum* (28.75±4.78) treatments. This study provides evidence that ~~native~~ endophytic isolates of *I. javanica* and *P. lilacinum* have a biocontrol potentials~~s~~ on whiteflies and could explored as plant growth promoters.

Keywords: biocontrol, endophyte, mortality, plant growth,

Introduction

Entomopathogenic fungi (EPF) are natural enemies of ~~the~~ insect population and are often regarded as effective biological control agents in integrated pest management (IPM) system [1,2]. Insect pests are usually controlled with EPF by either inundative or inoculative application of fungal propagules [3]. Several EPF have been commercialized for sap-sucking pest control [4]. In addition to direct applications, EPF has been discovered to colonize plants and grow endophytically, causing significant mortality of insect pests infesting plants including *B. tabaci* on tomato plants [5].

In many different plants, numerous genera of EPF have been isolated as endophytes. Some of them were identified as endophytes that occur naturally while others have been introduced into the plant artificially using different methods of inoculation [6]. ~~The~~ ascomycete~~s~~ fungi such as *Beauveria bassiana* and *Metarhizium anisopliae* are typical examples of fungal endophytes that naturally colonize plants and have also been artificially introduced into a wide variety of plants to reduce damage caused by insect pests including leaf-mining insects, and sap-sucking pests [4,7–10]. For example, the endophytic colonization of tomato~~es~~ by *B. bassiana* can significantly suppress *Tuta obsoluta* populations causing approximately 90% mortality [7,11].

In recent years, EPF species from the genera *Isaria* and *Purpureocillium* have been reported to artificially colonized plant tissues, promoting plant growth and mortality of insect pests [12,13]. ~~However,~~ fungal-plant interactions have been demonstrated to last for several months after inoculation, protecting the plants against insect attacks [11,14]. Depending on the inoculation techniques, endophytes vary in their ability to colonize various plant parts and survive over a plant growth cycle [10]. Inoculation techniques include seed treatment, root dipping, stem injection soil drenching, and foliar application [15]. A range of these

techniques has been studied for the endophytic colonization of tomato plants and efficiently promoting seedlings' growth and protecting them from attack by several insect pests.

In Malaysia where this study was conducted, little or no information can be found on the effects of EPF endophytes on the host plants and insect herbivores. However, there are some reports of endophytic colonization of some plants by other fungal isolates [16,17], but no reports are yet to be available on the EPF establishment as an endophyte in tomatoes and any other plants.

The use of EPF as an artificial endophyte in tomatoes could reduce damage caused by insect pests especially the whitefly, *Bemisia tabaci*, which has recently become one of the major threats to tomato production in Malaysia [18,19]. Therefore, the present study was conducted to evaluate the endophytic capability in tomato plants by some of the new native strains of EPF in Malaysia and their influence on plant growth, mortality, and adult emergence of *B. tabaci* as part of the IPM of whitefly..

Materials and methods

Plants, insects, and endophytic fungal isolates

Tomato seeds (MT1 variety) used in this study were obtained from the Malaysian Agricultural Research and Development Institute (MARDI). The stock culture of whiteflies that colonized tomato plants in the greenhouse was collected, reared, and maintained in a greenhouse under 25-30°C and 60-80% RH with a photoperiod of 12L:12D h light: dark, at the Faculty of Agriculture, Universiti Putra Malaysia (UPM). *B. tabaci* was identified by using mitochondrial COI (mt COI) sequencing with the primers (C1-J-2195 and L2-N-3014) as described by Frohlich et al. [20]. The sequencing data was registered and assigned the accession number OM638559 (GenBank). *B. tabaci* population were reared on tomato plants

for over 10 generations in a rearing cage at the Ladang 15, Faculty of Agriculture, Universiti Putra Malaysia (UPM) at (25-30°C and 60-80% RH with a photoperiod of 12L:12D h .Tomato plants were checked daily, and the damaged seedlings were replaced with new seedlings. The two fungal isolates, *I. javanica* (Cjc-03) and *P. lilacinum* (TS-01) used in this study were found naturally infesting ~~the whiteflies,~~ *B. tabaci* on chili and tomato plants and their virulence were evaluated against nymphs and adult *B. tabaci* under laboratory and glasshouse condition. The isolates were identified by morphological characterization and molecular identification (ITS region amplification).

The sequence data of the 2 isolates have been deposited in the NCBI GenBank database and the details of the isolates can be seen in Table S1 ~~table~~

Preparation of conidial suspension

The isolates were sub-cultured on potato dextrose agar medium (PDA) medium supplemented with 50mg/ml each of penicillin, streptomycin, and tetracycline. Conidia were harvested from 14 days old culture grown on PDA slants by flooding 0.01% of tween 80 on sporulated cultures and scrapped using a sterile spatula. Following that, the collected conidia were suspended in a 50 mL plastic tube, vortexed for 2 minutes, and filtered through four layers of muslin cloth to remove debris and create a clean stock suspension. ~~The~~ conidia viability was determined by inoculating 150µl of each fungal isolate suspension on the surface of fresh PDA plates [19]. Before incubation, a sterile microscope coverslip was placed on top of the agar on each plate. At 18-20 hours after incubation, the viability assessment was conducted under a light microscope at 400X magnifications by counting the number of germinated conidia out of 100 conidia in one randomly selected area. When the diameter of the conidium's germ tubes exceeded half of the diameter of the conidium, the

conidia were considered viable [10]. For each isolate, an average of three replicate counts was calculated.

Tomato seed inoculation

The seed immersion method was performed in this experiment by following the method of Allegrucci et al [7]. Seeds were surface sterilized using 70% ethanol for 5 minutes and rinsed 5 times with sterile distilled water. The treated seeds were placed on sterile tissue filter papers to dry for 30 minutes in a laminar flow before being inoculated and planted [7,11,22]. About 10 g of surface-sterilized seeds were subdivided into three equal portions, out of which two portions were individually soaked in a tube containing 10 ml of a conidial suspension of 1x10^{8} conidia/ ml prepared from each of the two isolates (Cjc-03 and TS-01). The third portion was soaked in sterile distilled water having 0.01% Tween 80 solution and served as a control treatment. At 24 hours after inoculation, the ~~inoculated~~ seeds were dried on sterile tissue paper in a laminar flow cabinet for 30 min, sown in multipots trays at a depth of 2 cm, containing approximately 20g of sterile peat moss planting media (previously autoclaved at 121° C for 30 minutes). The trays were maintained in the glasshouse at 25-30° C and 60-80%RH. During the experiments, plants were watered as needed and no fertilizer was used.

Assessment of endophytic colonization in inoculated tomato seedlings

Endophytic colonization was examined using two methods: (i) surface sterilization and PDA media plating, and (ii) live imaging using confocal laser-scanning microscopy (CLSM). For the first method, at each observation point, 5 seedlings were randomly selected per treatment for endophytic colonization assessment using a destructive sampling method [23]. The seedlings were then transported to the laboratory, washed, separated into leaves,

stems, and roots, and trimmed into smaller pieces of about 1 cm^2 with sterile stainless scissors. These parts were surface sterilized with 70% ethanol for 5 min, rinsed 5 times in sterile distilled water, and then placed on sterile tissue paper to dry in a laminar flow cabinet [22]. Five pieces of each leaf, stem, and root from each inoculated seedling were randomly taken and placed in a Petri dish containing a PDA slant, and each of the plates was labeled and replicated 3 times.

The Petri dishes containing the plant tissues were then incubated at 25±2 °C and inspected daily to assess them for fungal growth. Plant pieces that exhibited fungal growth were recorded. A total of 60 plants and 180 plant pieces were examined and the presence or absence of fungal endophytes on leaves, stems, and roots was recorded after 15, 30, 45, and 60 DAI.

Re-isolated fungal colonies were morphologically identified by comparing the mycelia and growth pattern with the original culture and by observing the conidia and conidiophores from prepared slides using a light microscope.

The data were expressed as:

$$\text{Colonization frequency} = \left(\frac{\text{Number of Plant Pieces Colonized}}{\text{Total Number of Plant pieces}}\right) \times 100$$

The second method, CLSM live imaging, was carried out as previously described by Nishi et al. [9]. A sterile stainless steel scissor was used to trim the sample of plant tissue to fit a glass microscope slide. CLSM observed samples within 1–2 hours of collection. About fifteen different plant parts were observed from each treatment. Selected images are representative of all images taken for a particular sample type.

Assessment of endophytic entomopathogenic fungi on tomato plant growth

The plant growth parameters: plant height, fresh weight, and dry weight were evaluated from the tomato plant used for endophytic colonization ~~of the tomato plant experiment~~ as described in the above section. The height of the tomato plant was measured with a ruler at 15, 30, 45, and 60 DAI. At 60 DAI, five seedlings from each of the treated and untreated control plants were harvested and taken to the laboratory for assessment of fresh and dry weight of the roots and aerial parts (stem and leaves). The fresh weight of the aerial parts and roots were weighed with an electronic balance and the same plant parts were placed inside a 20x20cm paper bag and placed in the oven at 60° C. After 3 days, the dried aerial and root parts were weighed using the same electronic balance [24].

Effect of endophytic entomopathogenic fungi on *B. tabaci* mortality and adult emergence

The hypothesis tested in this experiment was that tomato seedlings inoculated as seed ~~treatments~~ with two EPF isolates, Cjc-03 and TS-01 retains the ability to kill *B. tabaci* fed on colonized tomato plants. At 21 DAI, 6 seedlings from each treatment were transplanted in a 250ml pot (S1 Fig A). A transparent plastic container (15 cm heigh X 8 cm width) with fine mesh at the top was placed inside the rim of pots covering the aerial part of the plant (S1 Fig A). At 72 hours after transplanting, adult whiteflies were collected from a rearing cage with a manual aspirator and exposed to the aerial part of seedlings that had been grown from seeds inoculated with conidia of either *I. javanica* or *P. lilacinum* and untreated control seedlings. Afterward, 20-25 adult *B. tabaci* (≤ 5 days) were released in each confined pot with seedlings and adults were allowed to feed and oviposit for 7 days. The mortality of *B. tabaci* were recorded by counting the number of dead adults dropped off the plant in each cage. At 7 days

post *B. tabaci* release, all surviving adult insects were removed from the aerial parts of treated and control plants. Eggs laid on the leaflets were observed and seedlings were transferred to a new cage (20 cm height x10 cm width) for observing emergence of the first-generation adults (S1 Fig B). The eggs and subsequent stages were monitored over a period of 35 days, while quantifying the number of first-generation adults' *B.* tabaci.

Data analysis

The data of plant height were analyzed using factorial analysis of the variance, while the data for endophytic colonization, fresh and dry weight, population, and adult emergence of *B. tabaci* were analyzed with a one-way analysis of variance (ANOVA) using the package *R* statistical software Version 3.6.1, and significant differences between the means were compared with the LSD-Fischer test (p = 0.05).

Results

Assessment of endophytic colonization of inoculated tomato Seedlings

The result of this study on endophytic colonization of tomatoes showed that both of the EPF isolates examined (Cjc-03 and TS-01) successfully developed as endophytes in tomato plants (re-isolated from leaves, stem, and root) (S2 fig) following seed treatment. There was a decrease in colonization as assessed from the plant tissues plated on PDA medium after 30 days of inoculation and no *P. lilacinum* was re-isolated at 60 days after inoculation (Fig 1). No fungal isolates were re-isolated from the control plants.

The colonization frequency observed for both two isolates showed higher colonization on the stem and leaves at 15 and 30 days of assessment respectively and showed no significant difference of colonization for each day of assessment (15, 30 and 60 DAI), but

significantly differed in the day interval (stem: F = 0.31 p = 0.60; F = 5.15 p = 0.029: leaves: F = 4.35, p = 0.06; F = 17.58, p = 0.0005) (Figs 1A and B). On the other hand, the highest root colonization by two fungal isolates was recorded at 30 days and significantly differed at each day of assessment (15, 30 and 60 DAI) (F= 5.21 p = 0.045; F = 14.15, p = 0.001) (Fig 1C).Endophytic colonization of both isolates (Cjc-03 and TS-01) in tomato plants were also examined using live imaging by CLSM. ~~A noticeable~~ spore, germination ~~of~~ conidia and appressorium formation observed indicat~~ed~~ that the conidia ~~had~~ germinated on the tomato plant tissues (S3 Fig.1).

Effect of Endophytic entomopathogenic fungi on tomato seedling growth

The effects of inoculated EPF on the growth of tomato plants; height, number of leaves (leaflets), and fresh and dry weight are presented in figs 2 and 3. Data analyzed showed a significant increase of these parameters for tomato plants inoculated with EPF as compared to the control plant. At 15, 30, and 60 DAI, the following mean plant heights ± SE were recorded respectively: Cjc-03; 3.56 cm, 13.76 cm, 45.04 cm: TS-01; 5.90 cm, 14.06 cm, 46.28 cm (Fig 2A). The factorial analysis of variance showed significant effects of fungal endophyte, day after inoculation and their interaction on the height of tomato seedling (F =. 32.93, df = 2,32, $p < 0.001$; F = 820.91, df = 2,32, $p < 0.001$; F = 9.64, df = 4,32, $p < 0.01$. (Fig 3A).

The leaves (leaflets) number of tomato seedlings showed a significantly higher mean number when seeds were inoculated with Cjc-03 and TS-01 isolates (74.67and 80.67) in comparison to control (47.67) (F = 27.26, df = 2,6, $p< 0.001$) (Fig 2B).

At 60 DAI, analysis of variance (ANOVA) showed a significant increase in fresh and dry shoot and root biomass in seeds treated with Cjc-03 and TS-01 as compared to the control

seedlings. Fresh shoot and root showed a significant higher values weight (14.14g; 14.21 and 16.74g; 16.81g) of tomato seedlings colonized by Cjc-03 and TS-01 respectively, in comparison to the control plant (7.31g; 7.51g) (shoot of seedling F = 9.67, df = 2,6, p = 0.013; seedlings roots F = 4.83, df = 2,6, p = 0.046) (Figs 2A and B).

The mean value of the dry weight of shoots and roots was significantly higher in fungal colonized plants than in the control plants. (Shoot dry weight: F = 9.1143, df = 2,6, p = 0.015; root dry weight: F = 3.91df = 3,6 p = 0.05) (Figs 4A and B).

Effects of endophytically-colonized tomato seedlings on the mortality, and adult emergence of *B. tabaci*

The endophytic fungal isolates, *I. javanica* (Cjc-03) and *P. lilacinum* (TS-01) have significantly increased mortality rates of *B. tabaci* which fed on colonized tomato seedlings at 7 days after exposure to the aerial parts when compared with those of the control treatment. However, seedlings treated with Cjc-03 caused the highest reduction (51.92±4.78%) of *B. tabaci* population than those treated with TS-01 (45.32±0.20%) and there are no significant differences found among the plants treated with two isolates, but significantly differed from those of untreated control seedlings (19.29±2.35), (F = 31.36, df = 2,9, p = < 0.01) (Fig 5A).

Endophytic fungi caused reduction in adult *B. tabaci* emergence in the first generation. Both two isolates caused a significant reduction in number of adults emerging at assessment day (35 days post-exposure). A significantly high mean number of adults emergence was recorded in the control treatment (57.50±2.66) in comparison to the plants treated with Cjc-03 (15.00±1.47) and TS-0 (28.75±4.78) (F = 43.89, df = 2,9, p = < 0.01) (Fig 5B).

Discussion

The result of this research indicated that two EPF isolates have the capacity to colonize tomato plants endophytically which persisted in roots, stems, and leaves until the end of the trials (60 days). In addition, ~~inoculated~~ tomato seeds with *I. javanica*, and *P. lilacinum* isolates ~~have~~ significantly promoted plant growth and reduced survuval of *B. tabaci* adults which has fed on colonized tomato plants and also reduced adult emergence in comparison to control plants.

Previous research findings have reported the successful establishment of *I. javanica,* and *P. lilacinum* as endophytes for the control of insect pests in tomato plants and other economically important plants. Seed treatment of *Isaria* spp. has been successfully shown to be effective for endophytic colonization of pepper [25], eggplant [26], sorghum [27], and English oak [13]. Likewise, inoculation of *P. lilacinum* via seed treatment has been shown to colonize cotton [12,28].

The percentage of colonization of each isolate was shown to be proportional to the day of assessment with the second period (30 DAI) observed to have the highest percent colonization and the third period (60 DAI) to have the lowest percentage. A similar trend was observed by Mantzoukas et al. [27] who recorded high percent colonization of sorghum leaves and stem by *B. bassiana, M. robertsii,* and *Isaria fumosorosea* at 30 DAI. Similarly, Sun et al. [26] recorded the lowest percentage colonization of plant tissues of eggplant (leaf, stem, and root) by *C. fumosorosea* at 60 DAI.

The current study found that tomato seed inoculated with an isolate of *I. javanica* or *P. lilacinum* promoted growth and increased plant biomass when compared with the uninoculated control plants. There are few reports of plant inoculations with EPF species of

the genera *Isaria* and *Purpureocillium* promoting plant growth. For example, Sun et al. [26] reported that colonization of eggplant seedlings by *C. fumosorosea* increased plant height and other growth parameters including shoot length, root length, number of leaves, and weight of fresh shoot and root. However, *P. lilacinum* has been reported to increase the number of nodes and dry biomass of cotton plants [12]. Several factors have shown to enhance plant growth (shoot length, root length number of leaves, and weight of fresh shoot and root) some of them include, the ability to nurture nutrient uptake and to withstand different biotic and abiotic stress [26,29].

The endophytic colonization of tomato by *I. javanica* and *P. lilacinum* caused a significant mortality to whitefly and adult emerging at assessment day (35 days post-exposure) in comparison with the control plants. Although several studies demonstrated a significant reduction in the number of insect pests on different host plants when inoculated with EPF [4,7,8,30], but plant inoculations with fungal pathogens of the genera of *Isaria* and *Purpureocellium* were less reported to cause negative effects on *B. tabaci* and other insect pests. For instance, Sun et al. [26] reported that the inoculation of *C. fumosorosea* isolates in eggplant by seed immersion resulted in a reduction of the *B. tabaci* population. The inoculations of *B. bassiana* isolate in tomato plant by root drenching significantly reduced the number of eggs and nymphs of the greenhouse whitefly, *Trialeurodes vaporariorum* (Westwood) (Hemiptera: Aleyrodidae) in comparison to the uninoculated control plants [8]. Similarly, Lopez and Sword [12], reported a reduction in the population of *Helicoverpa zea* due to the endophytic action of *B. bassiana* and *Purpureocillium lilacinum* in cotton (*Gossypium hirsutum*).

Dead *B. tabaci* did not show any symptoms of mycosis. It has been previously reported that dead insects found in endophytically colonized plants show no symptoms of

fungal infection [10]. The mechanisms underlying the negative effects of endophytic EPF on *B. tabaci* are still largely unclear.

Conclusion

In conclusion, this research provides the first report on the endophytic colonization of plants with EPF ~~strains~~ and its direct impact on promoting plant growth ~~in Malaysia and some neighbouring countries~~. Endophytic colonization was detected following seed immersion techniques after inoculating 1x10^8 fungal suspension of *I. javanica* (Cjc-03) or *P. lilacinum* (TS-01). Both EPF endophytically colonized tomato seedlings and were recovered during 15, 30, and 60 DAI in the leaves, stems, and roots. Moreover, the plants treated with ~~EPF~~ endophyte ha~~ve~~ increased height and biomass ~~of tomato~~ in comparison to ~~those of~~ non-treated seedlings. In addition, both endophytic isolates ~~also~~ killed and reduced adult emergence ~~of *B. tabaci*~~.

Supporting information

S1 Fig. Bioassay cages used to evaluate the effect of endophytic EPF against *B. tabaci*. (A) Cage used to examine the effect of endophytic EPF on *B. tabaci* Population (B) Cage used to examine Adult Emergence of *B. tabaci*

S2 Fig. Examples of endophytic EPF (Cjc-03 and TS-01) re-isolation from the tomato plant tissues. (A) Leaf, (B) stem, and (C) root tissues following seed treatment with ~~a fungal~~ conidia suspensions

S3 Fig. Representative laser scanning confocal microscopy images of 30 days old tomato plant tissues colonized by endophytic EPF. (A) Cjc-03 (*I. javanica*) (B) TS-01 (*P. lilacinum*) with the noticeable spores indicated that the spores are alive on the plant tissues;

Germination of conidia and appressorium formation on the stem tissues of (C) Cjc-03 (*I. javanica*), (D) TS-01 (*P. lilacinum*).

S1 Table. Fungal endophytes used in the study. Species, accession number, origin, and EPF host species

Acknowledgments:

We thank Dr. Razak Terhem and Arisa Azim for technical assistance.

Author Contributions

Conceptualization: I.S., S.J., and J.J.

Data curation: S.A., N.S and I.S.

Formal analysis: J.J and I.S

Funding acquisition: N.S and S.J

Investigation: I.S., and E.M.H.

Methodology: J.J and I.S

Validation: S.J and S.I.I

Writing – original draft: I.S and JJ.

Writing – review & editing: S.J and S.I.I

References

1. Jiang W, Peng Y, Ye J, Wen Y, Liu G, Xie J. Effects of the entomopathogenic fungus *Metarhizium anisopliae* on the mortality and immune response of *Locusta migratoria.* Insects. 2020;11(1):1–12.
2. Sani I, Jamian S, Ismail SI, Saad N, Abdullah S, Hata EM, et al. Identification of entomopathogenic fungi *Metarhizium anisopliae* and *Purpureocillium lilacinum* from oil palm plantation soils in Universiti Putra Malaysia. Malays J Microbiol. 2022;18(1):105–12.
3. Liu F, Huang W, Wu K, Qiu Z, Huang Y, Ling E. Exploiting Innate Immunity for Biological Pest Control. In: Advances in Insect Physiology . Academic Press Inc.; 2017 . pp. 199–230. https://linkinghub.elsevier.com/retrieve/pii/S0065280617300012
4. González-Mas N, Sánchez-Ortiz A, Valverde-García P, Quesada-Moraga E. Effects of endophytic entomopathogenic ascomycetes on the life-history traits of *Aphis gossypii* glover and its interactions with melon plants. Insects. 2019;10(6):1–16.

5. Wei QY, Li YY, Xu C, Wu YX, Zhang YR, Liu H. Endophytic colonization by *Beauveria bassiana* increases the resistance of tomatoes against *Bemisia tabaci*. Arthropod Plant Interact . 2020;14(3):289–300. https://doi.org/10.1007/s11829-020-09746-9
6. Vega FE. Insect pathology and fungal endophytes. J Invertebr Pathol. 2008;98(3):277–9.
7. Allegrucci N, Velazquez MS, Russo ML, Perez E, Scorsetti AC. Endophytic colonisation of tomato by the entomopathogenic fungus *Beauveria bassiana:* The use of different inoculation techniques and their effects on the tomato leafminer *Tuta absoluta* (Lepidoptera: Gelechiidae). J Plant Prot Res. 2017;57(4):331–7.
8. Barra-Bucarei L, González MG, Iglesias AF, Aguayo GS, Peñalosa MG, Vera PV. *Beauveria bassiana* multifunction as an endophyte: Growth promotion and biologic control of *Trialeurodes vaporariorum*, (westwood) (hemiptera: Aleyrodidae) in tomato. Insects. 2020;11(9):1–15.
9. Nishi O, Sushida H, Higashi Y, Iida Y. Epiphytic and endophytic colonisation of tomato plants by the entomopathogenic fungus *Beauveria bassiana* strain GHA. Mycology . 2020;11:1–9. https://doi.org/10.1080/21501203.2019.1707723
10. Muvea AM, Meyhöfer R, Subramanian S, Poehling HM, Ekesi S, Maniania NK. Colonization of onions by endophytic fungi and their impacts on the biology of *Thrips tabaci*. PLoS One. 2014;9(9):1–7.
11. Silva ACL, Silva GA, Abib PHN, Carolino AT, Samuels RI. Endophytic colonization of tomato plants by the entomopathogenic fungus *Beauveria bassiana* for controlling the South American tomato pinworm, *Tuta absoluta*. CABI Agric Biosci . 2020;1(1):1–9. https://doi.org/10.1186/s43170-020-00002-x
12. Lopez DC, Sword GA. The endophytic fungal entomopathogens *Beauveria bassiana* and *Purpureocillium lilacinum* enhance the growth of cultivated cotton (*Gossypium hirsutum*) and negatively affect survival of the cotton bollworm (*Helicoverpa zea*). Biol Control . 2015;89:53–60. http://dx.doi.org/10.1016/j.biocontrol.2015.03.010
13. Vega FE. The use of fungal entomopathogens as endophytes in biological control: a review. Mycologia. 2018;110(1):4–30.
14. Garrido-Jurado, I., Resquı´n-Romero, G., Amarilla, S.P., Rı´os-Moreno, A., Carrasco, L., Quesada-Moraga E. Transient endophytic colonization of melon plants by entomopathogenic fungi after foliar application for the control of *Bemisia tabaci* Gennadius (Hemiptera: Aleyrodidae). J Pest Sci (2004). 2017;90:319–30.
15. Mantzoukas S, Eliopoulos PA. Endophytic entomopathogenic fungi: A valuable biological control tool against plant pests. Appl Sci. 2020;10(1):1–13.
16. Hamzah TNT, Lee SY, Hidayat A, Terhem R, Faridah-Hanum I, Mohamed R. Diversity and characterization of endophytic fungi isolated from the tropical mangrove species, *Rhizophora mucronata*, and identification of potential antagonists against the soil-borne fungus, *Fusarium solani*. Front Microbiol. 2018;9(01707):1–17.
17. Siddiquee S, Yusuf UK, Zainudin NAIM. Morphological and molecular detection of *Fusarium chlamydosporum* from root endophytes of *Dendrobium crumenatum*. African J Biotechnol. 2010;9(26):4081–90.
18. Saad KA, Roff MNM, Hallett RH, Idris AB. Aphid-induced Defences in Chilli Affect Preferences of the Whitefly, *Bemisia tabaci* (Hemiptera: Aleyrodidae). Sci Rep . 2015;5(13697):1–9. http://dx.doi.org/10.1038/srep13697
19. Sani, I., Ismail, S.I., Saad, N., Abdullah, S., Jalinas, J., Jamian S. Insect Pests of Vegetables in Malaysia and their Management using Entomopathogenic Fungi. Serangga. 2020;25(3):126–43.
20. Frohlich DR, Torres-Jerez I, Bedford ID, Markham PG, Brown JK. A phylogeographical analysis of the *Bemisia tabaci* species complex based on mitochondrial

DNA markers. Mol Ecol . 1999 ;8(10):1683–91. https://pubmed.ncbi.nlm.nih.gov/10583831/
21. Oliveira DGP, Pauli G, Mascarin GM, Delalibera I. A protocol for determination of conidial viability of the fungal entomopathogens *Beauveria bassiana* and *Metarhizium anisopliae* from commercial products. J Microbiol Methods. 2015;119:44–52.
22. Tefera T, Vidal S. Effect of inoculation method and plant growth medium on endophytic colonization of sorghum by the entomopathogenic fungus *Beauveria bassiana*. BioControl. 2009;54(5):663–9.
23. Bamisile BS, Senyo Akutse K, Dash CK, Qasim M, Ramos Aguila LC, Ashraf HJ, et al. Effects of Seedling Age on Colonization Patterns of Citrus limon Plants by Endophytic *Beauveria bassiana* and *Metarhizium anisopliae* and Their Influence on Seedlings Growth. J Fungi. 2020;6(1):29.
24. Canassa F, Tall S, Moral RA, Lara IAR d., Delalibera I, Meyling N V. Effects of bean seed treatment by the entomopathogenic fungi *Metarhizium robertsii* and *Beauveria bassiana* on plant growth, spider mite populations and behavior of predatory mites. Biol Control. 2019;132:199–208.
25. Bocco R, Lee M, Kim D, Ahn S, Park JW, Lee SY, et al. Endophytic *Isaria javanica* pf185 persists after spraying and controls *Myzus persicae* (Hemiptera: Aphididae) and *Colletotrichum acutatum* (Glomerellales: Glomerellaceae) in pepper. Insects. 2021;12(7):1–18.
26. Sun T, Shen Z, Shaukat M, Du C, Ali S. Endophytic isolates of *Cordyceps fumosorosea* to enhance the growth of *Solanum melongena* and reduce the survival of whitefly (*Bemisia tabaci*). Insects. 2020;11(2):8–10.
27. Mantzoukas S, Chondrogiannis C, Grammatikopoulos G. Effects of three endophytic entomopathogens on sweet sorghum and on the larvae of the stalk borer *Sesamia nonagrioides*. Entomol Exp Appl. 2015;154(1):78–87.
28. Lopez DC, Zhu-Salzman K, Ek-Ramos MJ, Sword GA. The entomopathogenic fungal endophytes *Purpureocillium lilacinum* (formerly *Paecilomyces lilacinus*) and Beauveria bassiana negatively affect cotton aphid reproduction under both greenhouse and field conditions. PLoS One. 2014;9(8):1–8.
29. Elena GJ, Beatriz PJ, Alejandro P, Roberto LE. *Metarhizium anisopliae* (Metschnikoff) Sorokin promotes growth and has endophytic activity in tomato plants. Adv Biol Res (Rennes). 2011;5(1):22–7.
30. Rasool S, Vidkjær NH, Hooshmand K, Jensen B, Fomsgaard IS, Meyling N V. Seed inoculations with entomopathogenic fungi affect aphid populations coinciding with modulation of plant secondary metabolite profiles across plant families. New Phytol. 2021;229(3):1715–27.

Figure
Click here to access/download;Figure;Figs_PlosOne_Manuscrip_With track

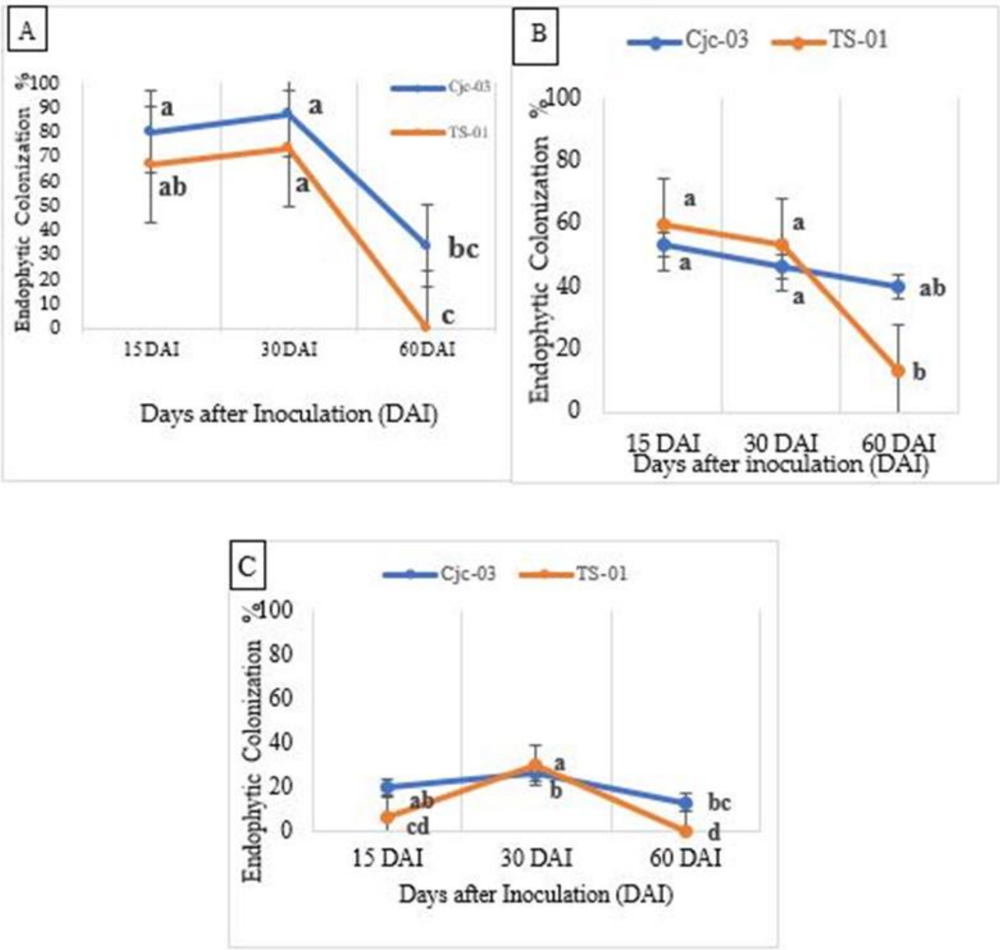

Fig 1. Percentage colonization of different plant parts (leaves, roots, and stems) of tomato ~~seedlings~~ plants inoculated with fungal isolates (Cjc-03 and TS-01) and evaluated at different time after inoculation (15, 30- and 60~~45~~-days). (A: leave ; B: Stem ; C: Root).

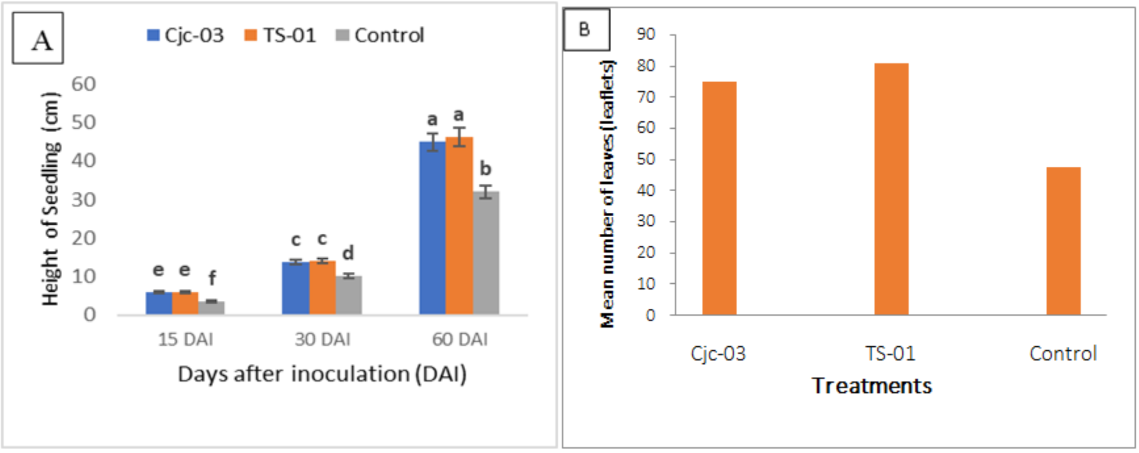

Fig 2. Mean height (±SE) of tomato seedlings and number of leaves (leaflets) after inoculations of tomato seeds with fungal isolates (Cjc-03 and TS-01) or control (sterile distilled water containing 0.01% Tween 80). (A): Length of tomato plants (shoot) measured at 15, 30, and 60 days after inoculations of tomato seeds with fungal suspension or control. (B): the number of leaves (leaflet) counted at 60 DAI of tomato seeds in fungal suspension or control. The different letters over the bars represent significant differences among the treatments according to Fisher's LSD test ($p < 0.05$).

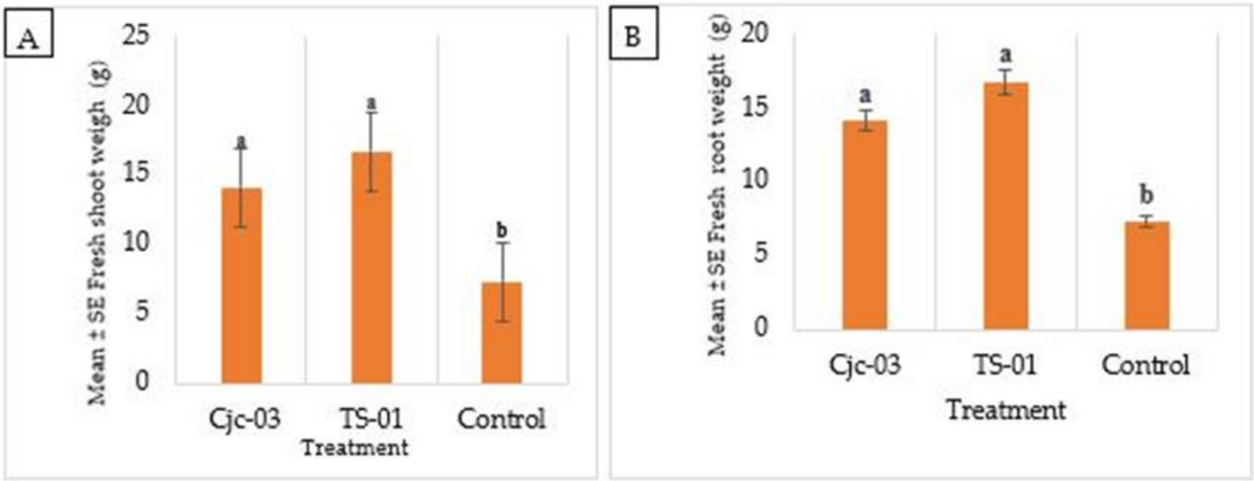

Fig 3.Mean (± SE) fresh weight (g) of tomato ~~seedlings~~ plants at 60 DAI treated with 2 EPF isolates of *C.* (Cjc-03 and TS-01) or sterile distilled water containing 0.01% Tween 80 (Control).(A) shoot weight (B), root weight. The different letters over the bars represent significant differences among the treatments according to Fisher's LSD test ($p < 0.05$).

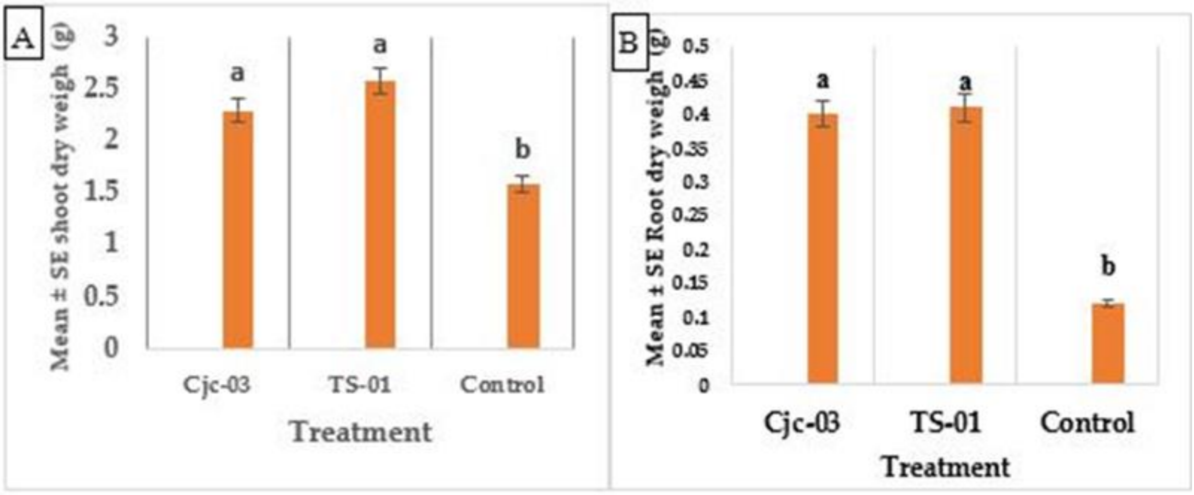

Fig 4.Mean (± SE) dry weight (g) of tomato seedlings at 60 DAI treated with 2 EPF isolates of (Cjc-03 and TS-01) or sterile distilled water containing 0.01% Tween 80 (Control). (A) Shoots weight (B), root weight. The different letters over the bars represent significant differences among the treatments according to Fisher's LSD test ($p < 0.05$).

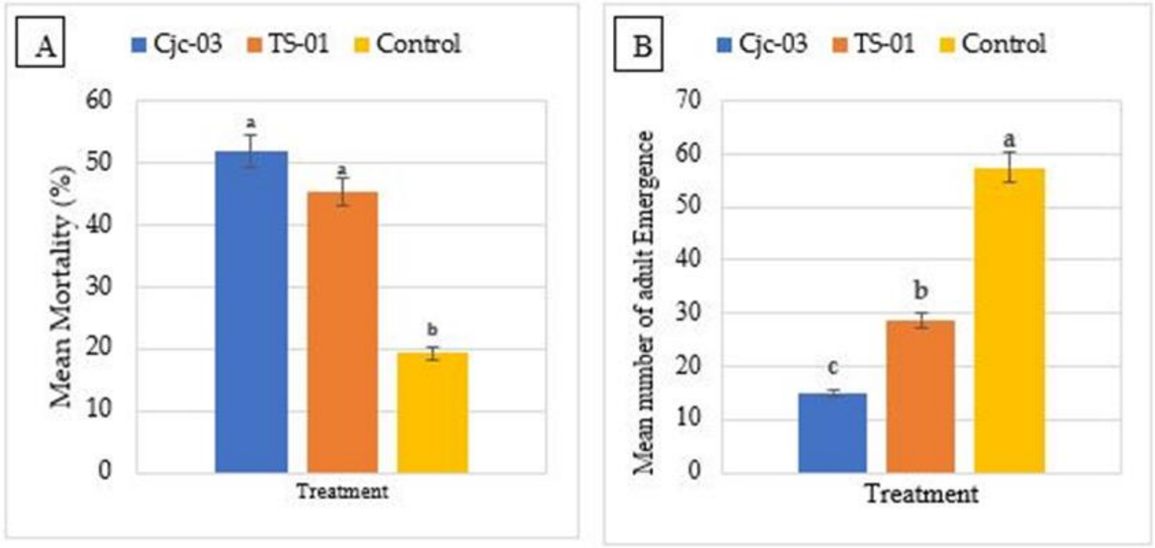

Fig 5. Effect of endophytic EPF isolates (Cjc-03 and TS-01) on the mortality and adult emergence of *B. tabaci* exposed to the inoculated tomato seedling (A): Mean percent mortality (SE ±) of adult *B. tabaci* exposed to tomato seedlings at 21 DAI. (B): Mean number (SE ±) of adult *B. tabaci* emergence on inoculated plants at 35 days after exposing adult *B.*

tabaci on inoculated tomato seedlings. Treatments with the same letter were not significantly different according to Fisher's LSD test at $p = 0.05$.

Figure

Click here to access/download;Figure;Figs_Manuscrip_Without track changes.docx

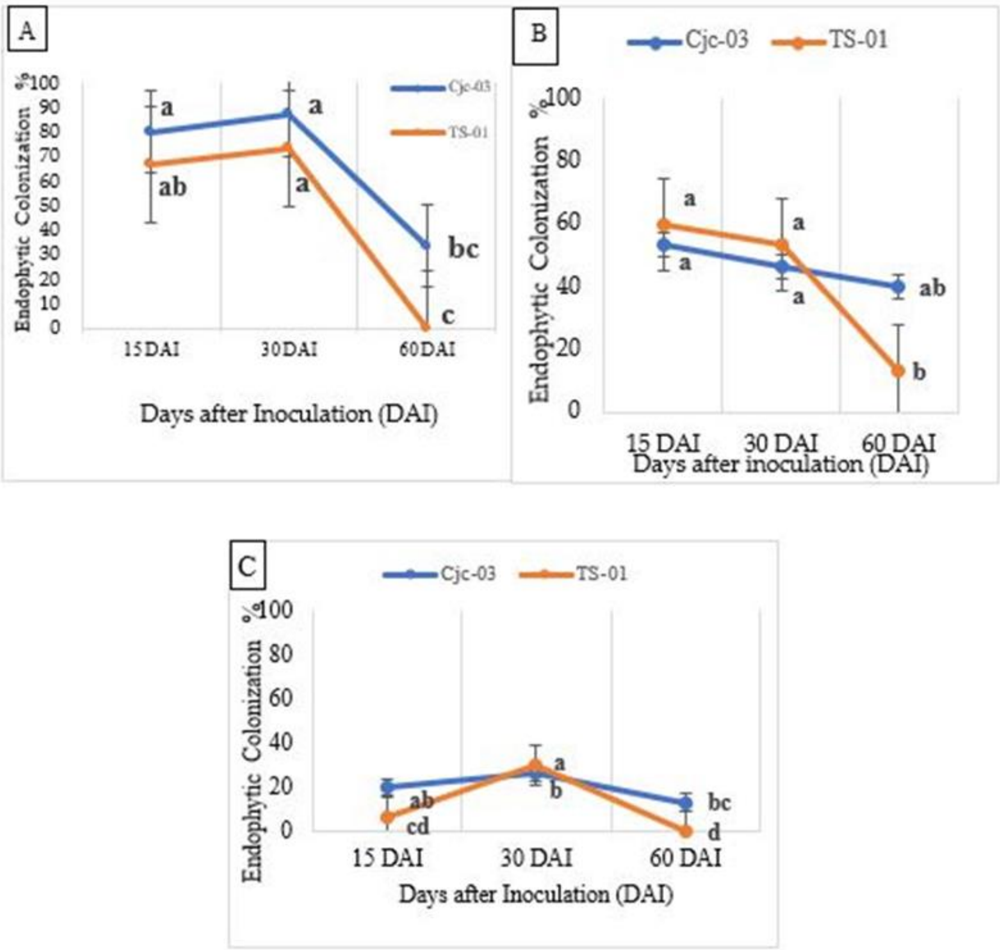

Fig 1. Percentage colonization of different plant parts (leaves, roots, and stems) of tomato plants inoculated with fungal isolates (Cjc-03 and TS-01) and evaluated at different time after inoculation (15, 30- and 60-days). (A: leave; B: Stem; C: Root).

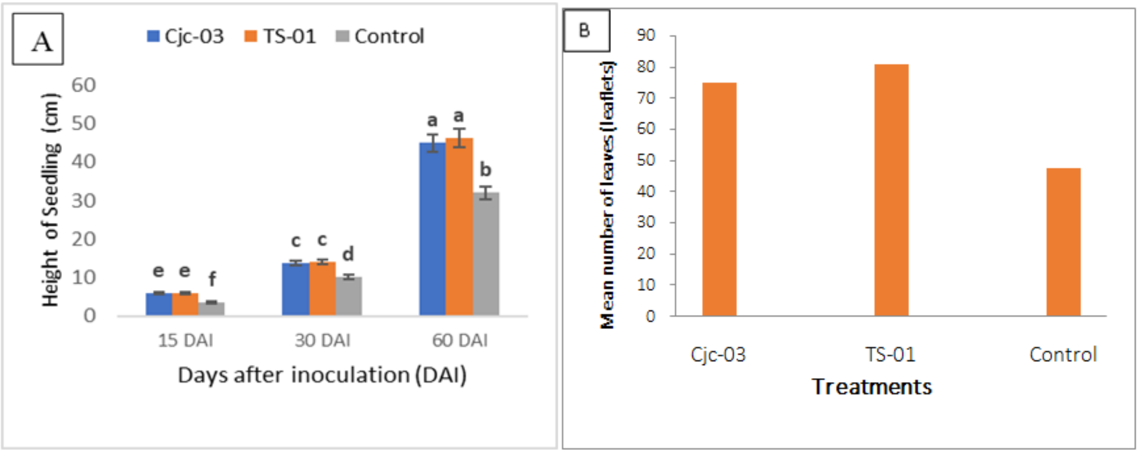

Fig 2. Mean height (±SE) of tomato seedlings and number of leaves (leaflets) after inoculations of tomato seeds with fungal isolates (Cjc-03 and TS-01) or control (sterile distilled water containing 0.01% Tween 80). (A): Length of tomato plants (shoot) measured at 15, 30, and 60 days after inoculations of tomato seeds with fungal suspension or control. (B): the number of leaves (leaflet) counted at 60 DAI of tomato seeds in fungal suspension or control. The different letters over the bars represent significant differences among the treatments according to Fisher's LSD test ($p < 0.05$).

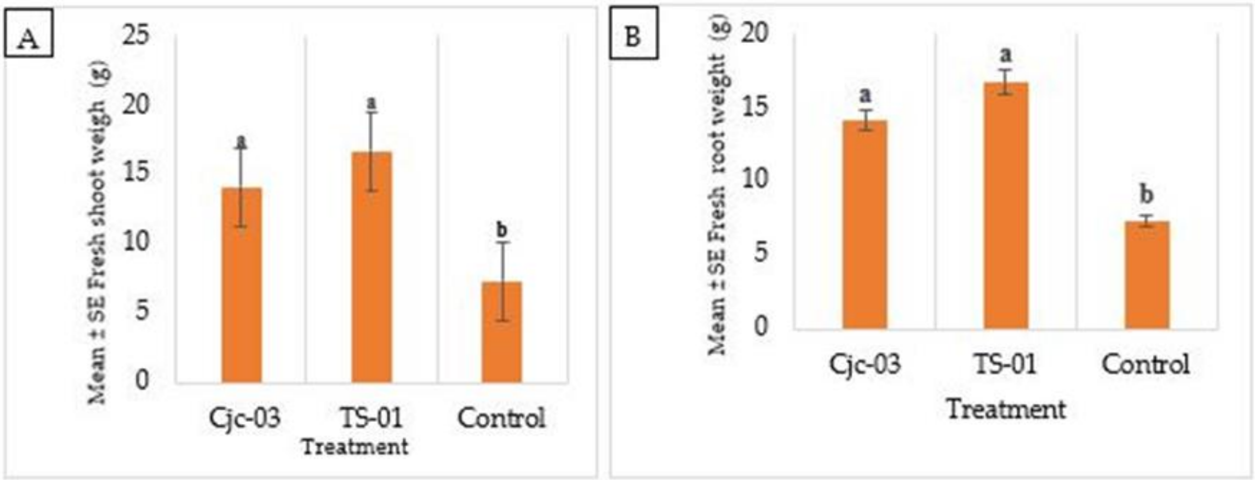

Fig 3.Mean (± SE) fresh weight (g) of tomato plants at 60 DAI treated with 2 EPF isolates of *C.* (Cjc-03 and TS-01) or sterile distilled water containing 0.01% Tween 80 (Control).(A) shoot weight (B), root weight. The different letters over the bars represent significant differences among the treatments according to Fisher's LSD test ($p < 0.05$).

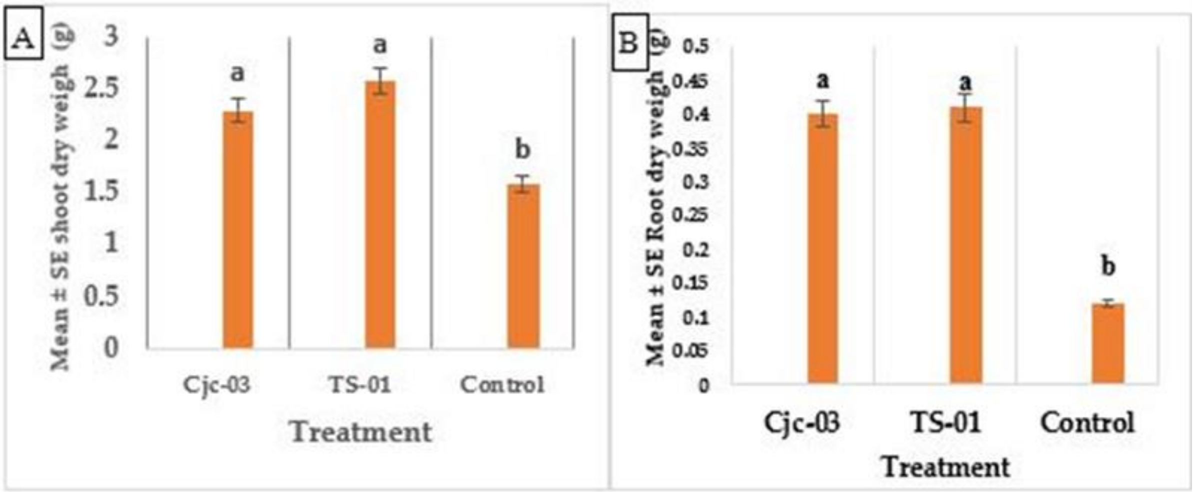

Fig 4.Mean (± SE) dry weight (g) of tomato seedlings at 60 DAI treated with 2 EPF isolates of (Cjc-03 and TS-01) or sterile distilled water containing 0.01% Tween 80 (Control). (A) Shoots weight (B), root weight. The different letters over the bars represent significant differences among the treatments according to Fisher's LSD test ($p < 0.05$).

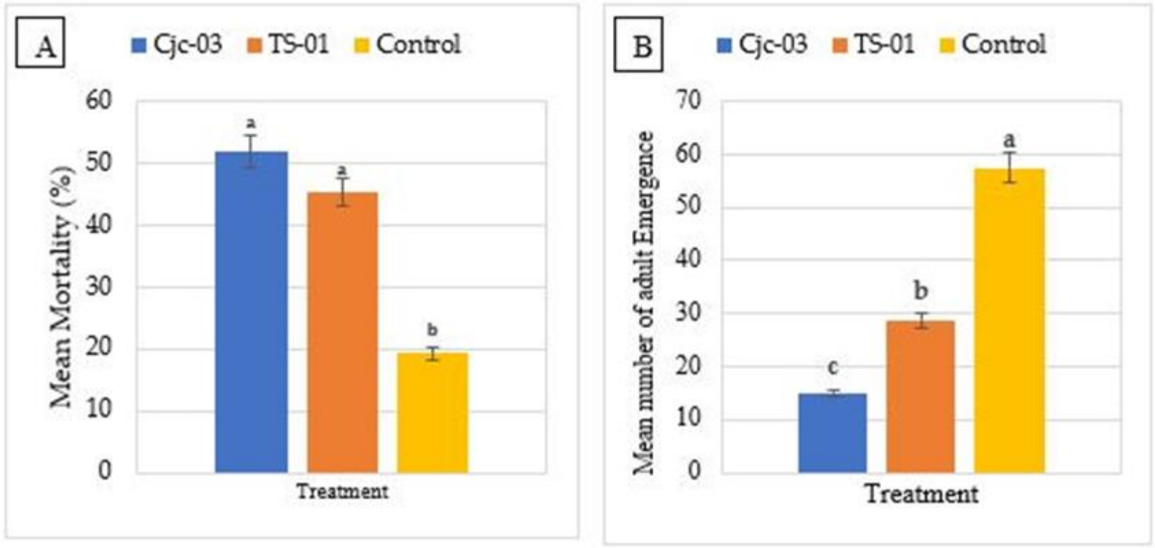

Fig 5. Effect of endophytic EPF isolates (Cjc-03 and TS-01) on the mortality and adult emergence of *B. tabaci* exposed to the inoculated tomato seedling (A): Mean percent mortality (SE ±) of adult *B. tabaci* exposed to tomato seedlings at 21 DAI. (B): Mean number (SE ±) of adult *B. tabaci* emergence on inoculated plants at 35 days after exposing adult *B.*

tabaci on inoculated tomato seedlings. Treatments with the same letter were not significantly different according to Fisher's LSD test at $p = 0.05$.

Click here to access/download
Supporting Information
Supporting information _No track changes.docx

Click here to access/download
Supporting Information
Supporting information _Plos one.docx

Inoculation and colonization of the entomopathogenic fungi, *Isaria javanica* and *Purpureocillium lilacinum*, in tomato plants, and their effect on seedling growth, mortality and adult emergence of Bemisia *tabaci* (Gennadius)

Ibrahim Sani[1,2], Syari Jamian[1,3*],Norsazilawati Saad[1], Sumaiyah Abdullah[1,]Erneeza Mohd Hata[4], Johari Jalinas[5] and Siti Izera Ismail[1,3*]

[1]Department of Plant Protection, Faculty of Agriculture, Universiti Putra Malaysia, Serdang 43400, Malaysia

[2] Department of Biology, Faculty of Natural and Applied Sciences, Umaru Musa Yar'adua University, P.M.B., Katsina 2218, Nigeria

[3] Laboratory of Climate-Smart Food Crop Production, Institute of Tropical Agriculture and Food Security (ITAFoS), Universiti Putra Malaysia, Serdang 43400, Malaysia

[4]Laboratory of Sustainable Agronomy & Crop Protection, Institute of Plantation Studies, Universiti Putra Malaysia, 43400 UPM Serdang, Selangor

[5] Centre for Insect Systematics, Department of Biological Sciences and Biotechnology, Faculty of Science & Technology, Universiti Kebangsaan Malaysia, Bangi 43600, Malaysia

syari@upm.edu.my (SJ);izera@upm.edu.my (SII)

Abstract

Entomopathogenic fungi (EPF) are natural enemies present in the insect population and have long been recognized as biological control agents against many insect pests. Some isolates have also been established as endophytes, benefiting their host plants without causing any symptoms or negative effects. Here we demonstrated two fungal species, *Isaria javanica* (Frieder. & Bally) Samson & Hywel-jone 2005 and *Purpureocillium lilacinum* (Thom) Luangsa-ard, Hou- braken, Hywel-Jones & Samson (2011) as an endophytes in tomato plants by using the seed inoculation method and examining their effect on plant growth, mortality, and adult emergence of *B. tabaci*.Our study indicated that tomato seeds treated with a fungal suspensions of *I. javanica* and *P. lilacinum* allowed their recovery from plant tissues (root, stem and leaf) up to 60 days after inoculation (DAI). Both endophytic isolates also significantly caused mortality of adult *B. tabaci* on seedlings inoculated with, *I. javanica* (51.92±4.78%), and *P. lilacinum* (45.32±0.20%) compared to the control treatment (19.29±2.35). However, the adult emergence mean number was significantly high in the control treatments (57.50±2.66) compared to *I. javanica* (15.00±1.47) and *P. lilacinum* (28.75±4.78) treatments.This study provides evidence that native endophytic isolates of *I. javanica* and *P. lilacinum* have a biocontrol potentials on whiteflies and could explored as plant growth promoters.

Keywords: biocontrol, endophyte, mortality, plant growth,

Introduction

Entomopathogenic fungi (EPF) are natural enemies of the insect population and are often regarded as effective biological control agents in an integrated pest management (IPM) system [1,2]. Insect pests are usually controlled with EPF by either inundative or inoculative application of fungal propagules [3]. Several EPF have been commercialized for sap-sucking pests control [4]. In addition to direct applications, EPF has been discovered to colonize plants and grow endophytically, causing significant mortality of insect pests infestingamong plants infested with pests including *B. tabaci* on tomato plants [5].

In many different plants, numerous genera of EPF have been isolated as endophytes. Some of them were identified as endophytes that occur naturally while others were have been introduced into the plant artificially using different methods of inoculation [6]. The ascomycetes fungi such as *Beauveria bassiana* and *Metarhizium anisopliae* are typical examples of fungal endophytes that naturally colonize plants and have also been artificially introduced into a wide variety of plants to reduce damage caused by insect pests including leaf-mining insects, and sap-sucking pests [4,7–10]. For example, the endophytic colonization of tomatoes by *B. bassiana* can significantly suppress *Tuta obsoluta* populations causing approximately 90% mortality [7,11].

In recent years, EPF species from the genera *Isaria* and *Purpureocillium* have been reported to artificially colonized plant tissues, promoting plant growth and mortality of insect pests [12,13]. However, fungal-plant interactions haves been demonstrated to last for several months after inoculation, protecting the plants against insect attacks [11,14]. Depending on the inoculation techniques, endophytes vary in their ability to colonize various plant parts and survive over a plant growth cycle [10]. Inoculation techniques include seed treatment, root dipping, stem injection soil drenching, and foliar application [15]. A range of these

techniques has been ~~artificially~~ studied for the endophytic colonization of tomato plants and efficiently promoting seedlings' growth and protecting them from ~~the~~ attack ~~of~~ by several insect pests.

In Malaysia where this study was conducted, little or no information can be found on the effects of ~~inoculated~~ EPF endophytes on the host plants and insect herbivores. However, there are some reports of endophytic colonization of some plants by other fungal isolates [16,17], but no reports are yet to be available on the EPF establishment as an endophyte in tomatoes and any other plants.

The use of EPF as an artificial endophyte in tomatoes could reduce damage caused by insect pests especially the whitefly, *Bemisia~~.~~ tabaci*, which has recently become one of the major threats to tomato production in Malaysia [18,19]. Therefore, the present study was conducted to evaluate the endophytic capability in tomato plants by some of the new native strains of EPF in Malaysia and their influence on plant growth, mortality, and adult emergence of *B. tabaci* as part of the IPM of whitefly..

Materials and methods

Plants, insects, and endophytic fungal isolates

Tomato seeds (MT1 variety) used in this study were obtained from the Malaysian Agricultural Research and Development Institute (MARDI). The stock culture of whiteflies that colonized tomato plants in the greenhouse was collected, reared, and maintained in a greenhouse under~~,~~ 25-30°C and 60-80% RH with a photoperiod of 12L:12D h light: dark~~,~~ at the Faculty of Agriculture, Universiti Putra Malaysia (UPM). *B. tabaci* was identified by using mitochondrial COI (mt COI) sequencing with the primers (C1-J-2195 and L2-N-3014) as described by Frohlich et al. [20]. The sequencing data was registered and assigned the

Field Code Changed

Field Code Changed

Formatted: Font color: Red

Formatted: Font color: Red

Formatted: Font: (Default) Times New Roman, 12 pt

Formatted: Font color: Red

Field Code Changed

accession number OM638559 (GenBank). *B. tabaci* population were reared on tomato plants for over 10 generations in a rearing cage at the Ladang 15, Faculty of Agriculture, Universiti Putra Malaysia (UPM) at (25-30°C and 60-80% RH with a photoperiod of 12L:12D h .Tomato plants were checked daily, and the damaged seedlings were replaced with new seedlings. The two fungal isolates, *I. javanica* (Cjc-03) and *P. lilacinum* (TS-01) used in this study were found naturally infesting the whiteflies, *B. tabaci* on chili and tomato plants and their virulence were evaluated against nymphs and adult *B. tabaci* under laboratory and glasshouse condition. The isolates were identified by morphological characterization and molecular identification (ITS region amplification).

The sequence data of the 2 isolates have been deposited in the NCBI GenBank database and the details of the isolates can be seen in Table S1 table

Preparation of conidial suspension

The isolates were sub-cultured on potato dextrose agar medium (PDA) medium supplemented with 50mg/ml each of penicillin, streptomycin, and tetracycline. Conidia were harvested from 14 days old culture grown on PDA slants by flooding 0.01% of tween 80 on sporulated cultures and scrapped using a sterile spatula. Following that, the collected conidia were suspended in a 50 mL plastic tube, vortexed for 2 minutes, and filtered through four layers of muslin cloth to remove debris and create a clean stock suspension. The conidia viability was determined by inoculating 150µl of each fungal isolate suspension on the surface of fresh PDA plates [19]. Before incubation, a sterile microscope coverslip was placed on top of the agar on each plate. At 18-20 hours after incubation, the viability assessment was conducted under a light microscope at 400X magnifications by counting the number of germinated conidia out of 100 conidia in one randomly selected area. When the diameter of the conidium's germ tubes exceeded~~s~~ half of the diameter of the conidium, the

Formatted: Font: Font color: Red

Field Code Changed

conidia were considered viable [10]. For each isolate, an average of three replicate counts was calculated.

Field Code Changed

Tomato seed inoculation

The seed immersion method was performed in this experiment by following the method of Allegrucci et al [7]. Seeds were surface sterilized using 70% ethanol for 5 minutes and rinsed 5 times with sterile distilled water. The treated seeds were placed on sterile tissue filter papers to dry for 30 minutes in a laminar flow before being inoculated and planted [7,11,22]. About 10 g of surface-sterilized seeds were subdivided into three equal portions, out of which two portions were individually soaked in a tube containing 10 ml of a conidial suspension of $1x10^8$ conidia/ ml prepared from each of the two isolates (Cjc-03 and TS-01) . The third portion was soaked in sterile distilled water having 0.01% Tween 80 solution and served as a control treatment. At 24 hours after inoculation, the inoculated seeds were dried on sterile tissue paper in a laminar flow cabinet for 30 min, sown in multipots trays at a depth of 2 cm, containing approximately 20g of sterile peat moss planting media (previously autoclaved at 121° C for 30 minutes). The trays were maintained in the glasshouse at 25-30° C and 60-80%RH. During the experiments, plants were watered as needed and no fertilizer was used.

Field Code Changed

Field Code Changed

Assessment of endophytic colonization in inoculated tomato seedlings

Endophytic colonization was examined using two methods: (i) surface sterilization and PDA media plating, and (ii) live imaging using confocal laser-scanning microscopy (CLSM). For the first method, at each observation point, 5 seedlings were randomly selected per treatment for endophytic colonization assessment using a destructive sampling method [23]. The seedlings were then transported to the laboratory, washed, separated into leaves,

Field Code Changed

stems, and roots, and trimmed into smaller pieces of about 1 cm^2 with sterile stainless scissors. These parts were surface sterilized with 70% ethanol for 5 min, rinsed 5 times in sterile distilled water, and then placed on sterile tissue paper to dry in a laminar flow cabinet [22]. Five pieces of each leaf, stem, and root from each inoculated seedling were randomly taken and placed in a P~~p~~etri dish containing a PDA slant, and each of the plates was labeled and replicated 3 times.

The Petri dishes containing the plant tissues were then incubated at 25±2 °C and inspected daily to assess them for fungal growth. Plant pieces that exhibited fungal growth were recorded. A total of 60 plants and 180 plant pieces were examined and the presence or absence of fungal endophytes on leaves, stems, and roots was recorded after 15, 30, 45, and 60 DAI.

Re-isolated fungal colonies were morphologically identified by comparing the mycelia and growth pattern with the original culture and by observing the conidia and conidiophores from prepared slides using a light microscope.

The data were expressed as:

$$\text{Colonization frequency} = \left(\frac{\text{Number of Plant Pieces Colonized}}{\text{Total Number of Plant pieces}}\right) \times 100$$

The second method, CLSM live imaging, was carried out as previously described by Nishi et al. [9]. A sterile stainless steel scissor was used to trim the sample of plant tissue to fit a glass microscope slide. CLSM observed samples within 1–2 hours of collection. about fifteen different plant parts were observed from each treatment. Selected images are representative of all images taken for a particular sample type.

Field Code Changed

Formatted: Font: Times New Roman

Formatted: Font: Times New Roman

Formatted: Font: Times New Roman

Formatted: Font: Times New Roman

Formatted: Font: Times New Roman

Formatted: Font: Times New Roman

Formatted: Font: Times New Roman

Formatted: Font: Times New Roman

Formatted: Font: Times New Roman

Formatted: Font: Times New Roman

Field Code Changed

Assessment of endophytic entomopathogenic fungi on tomato plant growth

The plant growth parameters: plant height, fresh weight, and dry weight were evaluated from the tomato plant used ~~in the~~for endophytic colonization of the tomato plant experiment as described in the above section. The height of the tomato plant was measured with a ruler at 15, 30, 45, and 60 DAI. At 60 DAI, five seedlings from each of the treated and untreated control plants were harvested and taken to the laboratory for ~~the~~ assessment of ~~the~~ fresh and dry weight of the roots and aerial parts (stem and leaves). The fresh weight of the aerial parts and roots were weighed with an electronic balance and the same plant parts were placed inside a 20x20cm paper bag and placed in the oven at 60° C. After 3 days, the dried aerial and root parts were weighed using the same electronic balance [24].

Formatted: Font color: Red

Field Code Changed

Effect of endophytic entomopathogenic fungi on *B. tabaci* mortality and adult emergence

Formatted: Heading 2, Left, Indent: First line: 0", Adjust space between Latin and Asian text, Adjust space between Asian text and numbers

The hypothesis tested in this experiment was that tomato seedlings inoculated as seed treatments with two EPF isolates, Cjc-03 and TS-01 ~~applied as seed treatment dispersed with roots in peatmoss substrate to stem and root and~~ retains the ability to kill *B. tabaci* fed on colonized tomato plants. At 21 DAI, 6 seedlings from each treatment were transplanted in a 250ml pot (S1 Fig A). A transparent plastic container (15 cm heigh X 8 cm width) with fine mesh at the top was placed inside the rim of pots covering the aerial part of the plant (S1 Fig A). At 72 hours after transplanting, ~~an~~ adult whiteflies were collected from a rearing cage with a manual aspirator and exposed to the aerial part of seedlings that had been grown from seeds inoculated with conidia of either *I. javanica* or *P. lilacinum* and untreated control

Formatted: Font color: Red

Formatted: Font color: Red

seedlings. Afterward, 20-25 adult *B. tabaci* (≤ 5 days)were released in each confined pot with seedlings and adults were allowed to feed and oviposit for 7 days. The mortality of *B. tabaci* were recorded by counting the number of dead adults dropped off the plant ion each cage. At 7 days post *B. tabaci* release,- all surviving adult insects were removed from the aerial parts of treated and control plants. Eggs laid on the leaflets were observed and seedlings were transferred to a new cage (20 cm height x10 cm width) for observing adult emergence of the first-generation adults (S1 Fig B). The eggs and subsequent stages were monitored over a period of 35 days, while quantifying the number of recording first-generation adults *B.* tabaci.

Formatted: English (Malaysia)

Data analysis

The data of plant height were analyzed using factorial analysis of the variance, while the data for endophytic colonization, fresh and dry weight, population, and adult emergence of *B. tabaci* were analyzed with a one-way analysis of variance (ANOVA) using the package *R* statistical software Version 3.6.1, and significant differences between the means were compared with the LSD-Fischer test (p = 0.05).

Results

Assessment of endophytic colonization of inoculated tomato Seedlings

The result of this study on endophytic colonization of tomatoes showed that both of the –EPF isolates examined (Cjc-03 and TS-01) successfully developed as endophytes in tomato plants (re-isolated from leaves, stem, and root) (S2 fig) following seed treatment. There was a decrease in colonization as assessed from the surface sterilized plant tissues plated on PDA medium after 30 days of inoculation and no *P. lilacinum* was re-isolated at 60 days after inoculation (Fig 1). No fungal isolates were re-isolated from the control plants.

~~However, surface sterilization is effective and successful because there was no fungal growth on the PDA medium that was plated with the last rinses of distilled water used for surface sterilization of tomato tissues.~~

The ~~result of~~ colonization frequency observed for both two isolates ~~resulted~~ showed ~~in~~ higher colonization on the stem and leaves at 15 and 30 days of assessment respectively and showed no significant difference of colonization ~~on~~ for each day of assessment (15, 30 and 60 DAI), but significantly differed in the day interval ~~of assessment~~ (stem: F = 0.31 *p* = 0.60 ; F = 5.15 *p* = 0.029: leaves: F = 4.35, *p* = 0.06; F = 17.58, *p* = 0.0005) (Figs 1A and B). On the other hand, the highest root colonization by two fungal isolates was recorded at 30 days ~~of assessment~~ and significantly differed at each day of assessment (15, 30 and 60 DAI) (F= 5.21 *p* = 0.045; F = 14.15, *p* = 0.001) (Fig 1C).

Endophytic colonization of both isolates (Cjc-03 and TS-01) in tomato plants was also examined using live imaging by CLSM. A noticeable spore, germination of conidia and appressorium formation observed indicated that the ~~spores~~ conidia ~~are~~ had ~~alive~~ germinated on the tomato plant tissues (S3 Fig.1).

Formatted: Not Highlight

Formatted: Not Highlight

Formatted: Not Highlight

Formatted: Font: (Default) Times New Roman, 12 pt, Not Italic, English (United States)

Effect of Endophytic entomopathogenic fungi on tomato seedling growth

The effects of inoculated EPF on the growth of tomato plants; height, number of leaves (leaflets), and fresh and dry weight are presented in figs 2 and 3. Data analyzed showed a significant increase of these parameters ~~on~~ for tomato plants inoculated with EPF as compared to the control plant. At 15, 30, and 60 DAI, the following mean plant heights ± SE were recorded respectively: Cjc-03; 3.56 cm, 13.76 cm, 45.04 cm: TS-01; 5.90 cm, 14.06 cm, 46.28 cm (Fig 2A). The factorial analysis of variance showed significant effects of fungal endophyte, day after inoculation and their interaction on the height of tomato seedling (F =.

32.93, df = 2,32, p < 0.001; F = 820.91, df = 2,32, p < 0.001; F = 9.64, df = 4,32, p < 0.01. (Fig 3A).

The leaves (leaflets) number of tomato seedlings showed a significantly higher mean number when seeds were inoculated ~~by~~ with Cjc-03 and TS-01 isolates ~~showed a high and significant mean number~~ (74.67and 80.67) in comparison~~ed~~ to ~~those in~~ control ~~tomato plants~~ (47.67) (F = 27.26, df = 2,6, p< 0.001) (Fig 2B).

At 60 DAI, analysis of variance (ANOVA) showed a significant increase in fresh and dry shoot and root biomass in seeds~~lings~~ treated ~~seeds~~ with Cjc-03 and TS-01 ~~isolates~~ as compared to the control seedlings. Fresh shoot and root showed a significant higher values weight (14.14g; 14.21 and 16.74g; 16.81g) of tomato seedlings colonized by Cjc-03 and TS-01 respectively, in comparison to the control plant (7.31g; 7.51g) (shoot of seedling F = 9.67, df = 2,6, p = 0.013; ~~root of~~ seedlings roots F = 4.83, df = 2,6, p = 0.046) (Figs 2A and B).

The mean value of the dry weight of shoots and roots ~~parts~~ was significantly higher in fungal colonized plants than in the control plants. (Shoot dry weight: F = 9.1143, df = 2,6, p = 0.015; root dry weight: F = 3.91df = 3,6 p = 0.05) (Figs 4A and B).

Effects of endophytically-colonized tomato seedlings on the mortality, and adult emergence of *B. tabaci*

The endophytic fungal isolates, *I. javanica* (Cjc-03) and *P. lilacinum* (TS-01) have significantly increased mortality rates ~~-~~of *B. tabaci* ~~feeding~~ which fed on colonized tomato seedlings at 7 days after exposure to the aerial parts- when compared with those of the control treatment. However, seedlings treated with Cjc-03 ~~isolate~~ caused the highest reduction (51.92±4.78%) of *B. tabaci* population than those treated with TS-01 (45.32±0.20%) ~~isolate~~ and there are no significant differences found among the plants treated with two isolates, but

significantly differed ~~with~~ from those of untreated control seedlings (19.29±2.35), ($F = 31.36$, df = 2,9, $p = < 0.01$) (Fig 5A).

.

Endophytic fungi ~~mediated~~ caused reduction in adult *B. tabaci* emergence in the first generation. Both two isolates ~~induced~~ caused a significant reduction in number of adults emergingi~~ed~~ at ~~assesment~~assessment day (35 days post-exposure). A significantly high mean number of adults emergence was recorded in the control treatment (57.50±2.66) in comparison to the plants treated with Cjc-03 (15.00±1.47) and TS-0 (28.75±4.78) ($F = 43.89$, df = 2,9, $p = < 0.01$) (Fig 5B).

Discussion

The result of this research indicated~~s~~ that two EPF isolates have the capacity to colonize tomato plants endophytically which persisted in roots, stems, and leaves until the end of the trials (60 days). In addition, inoculated tomato seeds~~-~~ with *I. javanica*, and *P. lilacinum* isolates have significantly promoted plant growth and reduced, survuval of *B. tabaci* adults which has fed on colonized tomato plants ~~the population~~ and also reduced adult emergence ~~of *B. tabaci*~~ in comparison to control plants.

Previous research findings have reported the successful establishment of *I. javanica*, and *P. lilacinum* as endophytes for the control of insect pests in tomato plants and other economically important plants. Seed treatment of *Isaria* spp. has been successfully shown to be effective for endophytic colonization of p~~P~~epper [25], eggplant [26], sorghum [27], and English oak [13]. Likewise, inoculation of *P. lilacinum* via seed treatment has been shown to colonize cotton [12,28].

Formatted: Font: (Default) Times New Roman

Formatted: Font: (Default) Times New Roman, Italic

Formatted: Font: (Default) Times New Roman

Field Code Changed

Field Code Changed

Field Code Changed

Field Code Changed

Field Code Changed

The percentage of colonization of each isolate was shown to be proportional to the day of assessment with the second period (30 DAI) observed to have the highest percent colonization and the third period (60 DAI) to have the lowest percentage. A similar trend was observed by Mantzoukas et al. [27] who recorded high percent colonization of sorghum leaves and stem by *B. bassiana*, *M. robertsii*, and *Isaria fumosorosea* at 30 DAI. Similarly, Sun et al. [26] recorded the lowest percentage colonization of plant tissues of eggplant (leaf, stem, and root) by *C. fumosorosea* at 60 DAI.

The current study found that tomato seed inoculated with an isolate of *I. javanica* or *P. lilacinum* promoted growth and increased plant biomass when compared with the uninoculated control plants. There are few reports of plant inoculations with EPF species of the genera *Isaria* and *Purpureocillium* promoting plant growth. For example, Sun et al. [26] reported that colonization of eggplant seedlings by *C. fumosorosea* increased plant height and other growth parameters including shoot length, root length, number of leaves, and weight of fresh shoot and root. However, *P. lilacinum* has been reported to increase the number of nodes and dry biomass of cotton plants [12]. Several factors have ~~been demonstrated~~ shown to ~~fungal endophytes to~~ enhance plants~~'~~ growth (shoot length, root length number of leaves, and weight of fresh shoot and root) some of them include, the ability to nurture nutrient uptake and to withstand different biotic and abiotic stress [26,29].

The endophytic colonization of tomato by~~–~~ *I. javanica* and *P. lilacinum* caused a significant ~~reduction~~ mortality ~~in~~ to whitefly ~~populations~~ and adult emergening~~ce~~ at assessment day (35 days post-exposure) ~~following seed inoculation~~ in comparison with the control plants. Although several studies demonstrated a significant reduction in the number of insect pests on different host plants when inoculated with EPF [4,7,8,30], but plant inoculations with fungal pathogens of the genera of *Isaria* and *Purpureocellium* ~~have been~~were less reported to cause negative effects on *B. tabaci* and other insect pests. For

Field Code Changed

Field Code Changed

Field Code Changed

Field Code Changed

Field Code Changed

Formatted: Not Highlight

Formatted: Not Highlight

Formatted: Not Highlight

Field Code Changed

Formatted: Not Highlight

instance, Sun et al. [26] reported that the inoculation of *C. fumosorosea* isolates in eggplant by seed immersion resulted in a reduction of the *B. tabaci* population. The inoculations of *B. bassiana* isolate in tomato plant by root drenching significantly reduced the number of eggs and nymphs of the greenhouse whitefly, *Trialeurodes vaporariorum* (Westwood) (Hemiptera: Aleyrodidae) in comparison to the uninoculated control plants [8]. Similarly, Lopez and Sword [12], reported a reduction in the population of *Helicoverpa zea* due to the endophytic action of *B. bassiansa* and *Purpureocillium lilacinum* in cotton (*Gossypium hirsutum*).

~~The~~ ~~D~~Ddead *B. tabaci* did not show any symptoms of mycosis. It has been previously reported that dead insects found in endophytically colonized plants show no symptoms of fungal infection [10]**.** The mechanisms underlying the negative effects of endophytic EPF on *B. tabaci* are still largely unclear. ~~However, based on our current study, EPF isolates, Cjc-03 and TS-01 applied as seed treatment, dispersed with roots in peatmoss substrate and root and retained the ability to kill *B. tabaci*.~~

Conclusion

In conclusion, this research provides the first report on the endophytic colonization of plants with EPF strains and its direct impact on promoting plant growth in Malaysia and some neighboring countries. Endophytic colonization ~~has been~~was detected ~~with~~ following seed immersion techniques after inoculating 1x10^8 fungal suspension ~~of one isolate each~~ of *I. javanica* (Cjc-03) or *P. lilacinum* (TS-01). Both EPF endophytically colonized ~~endophytically~~ tomato seedlings and were recovered during 15, 30, and 60 DAI in the leaves, stems, and roots. Moreover, the plants treated with EPF endophyte have ~~improved~~ increased ~~the~~ height and biomass of tomato ~~seedlings~~ in comparison to those of non-treated seedlings.

Field Code Changed

Field Code Changed

Field Code Changed

Field Code Changed

Formatted: Font: (Default) Times New Roman, Kern at 18 pt

In addition, both endophytic isolates also killed~~reduced the population~~ and reduced adult emergence of *B. tabaci*. ~~on tomato seedlings as compared to the control treatment~~.

~~Furthermore, research on the endophytic inoculation and efficacy of EPFs on different plants and insect pests could be utilized for the development of new IPM strategies.~~

Supporting information

S1 Fig. Bioassay cages used to evaluate the effect of endophytic EPF against *B. tabaci*.(A) cage used to examine the effect of endophytic EPF on *B. tabaci* Population(B)Cage used to examine Adult Emergence of *B. tabaci*

S2 Fig. Examples of endophytic EPF (Cjc-03 and TS-01) re-isolation from the tomato plant tissues. (A) Leaf, (B) stem, and (C) root tissues following seed treatment with a fungal conidia suspensions

S3 Fig. Representative laser scanning confocal microscopy images of 30 days old tomato plant tissues colonized by endophytic EPF. (A) Cjc-03 (*I. javanica*) (B) TS-01 (*P. lilacinum*) with the noticeable spores indicated that the spores are alive on the plant tissues; Germination of conidia and appressorium formation on the stem tissues of (C) Cjc-03 (*I. javanica*), (D) TS-01 (*P. lilacinum*).

S1 Table. Fungal endophytes used in the study. Species, accession number, origin, and EPF host species

Acknowledgments:

We thank Dr. Razak Terhem and Arisa Azim for technical assistance.

Author Contributions

Conceptualization: I.S., S.J., and J.J**.**

Data curation: S.A., N.S and I.S**.**

Formal analysis: J.J and I.S

Funding acquisition: N.S and S.J

Investigation: I.S., and E.M.H.

Methodology: J.J and I.S

Validation: S.J and S.I.I

Writing – original draft: I.S and JJ.

Writing – review & editing: S.J and S.I.I

References

1. Jiang W, Peng Y, Ye J, Wen Y, Liu G, Xie J. Effects of the entomopathogenic fungus *Metarhizium anisopliae* on the mortality and immune response of *Locusta migratoria.* Insects. 2020;11(1):1–12.
2. Sani I, Jamian S, Ismail SI, Saad N, Abdullah S, Hata EM, et al. Identification of entomopathogenic fungi *Metarhizium anisopliae* and *Purpureocillium lilacinum* from oil palm plantation soils in Universiti Putra Malaysia. Malays J Microbiol. 2022;18(1):105–12.
3. Liu F, Huang W, Wu K, Qiu Z, Huang Y, Ling E. Exploiting Innate Immunity for Biological Pest Control. In: Advances in Insect Physiology . Academic Press Inc.; 2017 . pp. 199–230. https://linkinghub.elsevier.com/retrieve/pii/S0065280617300012
4. González-Mas N, Sánchez-Ortiz A, Valverde-García P, Quesada-Moraga E. Effects of endophytic entomopathogenic ascomycetes on the life-history traits of *Aphis gossypii* glover and its interactions with melon plants. Insects. 2019;10(6):1–16.
5. Wei QY, Li YY, Xu C, Wu YX, Zhang YR, Liu H. Endophytic colonization by *Beauveria bassiana* increases the resistance of tomatoes against *Bemisia tabaci*. Arthropod Plant Interact . 2020;14(3):289–300. https://doi.org/10.1007/s11829-020-09746-9
6. Vega FE. Insect pathology and fungal endophytes. J Invertebr Pathol. 2008;98(3):277–9.
7. Allegrucci N, Velazquez MS, Russo ML, Perez E, Scorsetti AC. Endophytic colonisation of tomato by the entomopathogenic fungus *Beauveria bassiana:* The use of different inoculation techniques and their effects on the tomato leafminer *Tuta absoluta* (Lepidoptera: Gelechiidae). J Plant Prot Res. 2017;57(4):331–7.
8. Barra-Bucarei L, González MG, Iglesias AF, Aguayo GS, Peñalosa MG, Vera PV. *Beauveria bassiana* multifunction as an endophyte: Growth promotion and biologic control of *Trialeurodes vaporariorum*, (westwood) (hemiptera: Aleyrodidae) in tomato. Insects. 2020;11(9):1–15.

Field Code Changed

9. Nishi O, Sushida H, Higashi Y, Iida Y. Epiphytic and endophytic colonisation of tomato plants by the entomopathogenic fungus *Beauveria bassiana* strain GHA. Mycology . 2020;11:1–9. https://doi.org/10.1080/21501203.2019.1707723
10. Muvea AM, Meyhöfer R, Subramanian S, Poehling HM, Ekesi S, Maniania NK. Colonization of onions by endophytic fungi and their impacts on the biology of *Thrips tabaci*. PLoS One. 2014;9(9):1–7.
11. Silva ACL, Silva GA, Abib PHN, Carolino AT, Samuels RI. Endophytic colonization of tomato plants by the entomopathogenic fungus *Beauveria bassiana* for controlling the South American tomato pinworm, *Tuta absoluta*. CABI Agric Biosci . 2020;1(1):1–9. https://doi.org/10.1186/s43170-020-00002-x
12. Lopez DC, Sword GA. The endophytic fungal entomopathogens *Beauveria bassiana* and *Purpureocillium lilacinum* enhance the growth of cultivated cotton (*Gossypium hirsutum*) and negatively affect survival of the cotton bollworm (*Helicoverpa zea*). Biol Control . 2015;89:53–60. http://dx.doi.org/10.1016/j.biocontrol.2015.03.010
13. Vega FE. The use of fungal entomopathogens as endophytes in biological control: a review. Mycologia. 2018;110(1):4–30.
14. Garrido-Jurado, I., Resquı´n-Romero, G., Amarilla, S.P., Rı´os-Moreno, A., Carrasco, L., Quesada-Moraga E. Transient endophytic colonization of melon plants by entomopathogenic fungi after foliar application for the control of *Bemisia tabaci* Gennadius (Hemiptera: Aleyrodidae). J Pest Sci (2004). 2017;90:319–30.
15. Mantzoukas S, Eliopoulos PA. Endophytic entomopathogenic fungi: A valuable biological control tool against plant pests. Appl Sci. 2020;10(1):1–13.
16. Hamzah TNT, Lee SY, Hidayat A, Terhem R, Faridah-Hanum I, Mohamed R. Diversity and characterization of endophytic fungi isolated from the tropical mangrove species, *Rhizophora mucronata*, and identification of potential antagonists against the soil-borne fungus, *Fusarium solani*. Front Microbiol. 2018;9(01707):1–17.
17. Siddiquee S, Yusuf UK, Zainudin NAIM. Morphological and molecular detection of *Fusarium chlamydosporum* from root endophytes of *Dendrobium crumenatum*. African J Biotechnol. 2010;9(26):4081–90.
18. Saad KA, Roff MNM, Hallett RH, Idris AB. Aphid-induced Defences in Chilli Affect Preferences of the Whitefly, *Bemisia tabaci* (Hemiptera: Aleyrodidae). Sci Rep . 2015;5(13697):1–9. http://dx.doi.org/10.1038/srep13697
19. Sani, I., Ismail, S.I., Saad, N., Abdullah, S., Jalinas, J., Jamian S. Insect Pests of Vegetables in Malaysia and their Management using Entomopathogenic Fungi. Serangga. 2020;25(3):126–43.
20. Frohlich DR, Torres-Jerez I, Bedford ID, Markham PG, Brown JK. A phylogeographical analysis of the *Bemisia tabaci* species complex based on mitochondrial DNA markers. Mol Ecol . 1999 ;8(10):1683–91. https://pubmed.ncbi.nlm.nih.gov/10583831/
21. Oliveira DGP, Pauli G, Mascarin GM, Delalibera I. A protocol for determination of conidial viability of the fungal entomopathogens *Beauveria bassiana* and *Metarhizium anisopliae* from commercial products. J Microbiol Methods. 2015;119:44–52.
22. Tefera T, Vidal S. Effect of inoculation method and plant growth medium on endophytic colonization of sorghum by the entomopathogenic fungus *Beauveria bassiana*. BioControl. 2009;54(5):663–9.
23. Bamisile BS, Senyo Akutse K, Dash CK, Qasim M, Ramos Aguila LC, Ashraf HJ, et al. Effects of Seedling Age on Colonization Patterns of Citrus limon Plants by Endophytic *Beauveria bassiana* and *Metarhizium anisopliae* and Their Influence on Seedlings Growth. J Fungi. 2020;6(1):29.
24. Canassa F, Tall S, Moral RA, Lara IAR d., Delalibera I, Meyling N V. Effects of bean seed treatment by the entomopathogenic fungi *Metarhizium robertsii* and *Beauveria bassiana*

on plant growth, spider mite populations and behavior of predatory mites. Biol Control. 2019;132:199–208.
25. Bocco R, Lee M, Kim D, Ahn S, Park JW, Lee SY, et al. Endophytic *Isaria javanica* pf185 persists after spraying and controls *Myzus persicae* (Hemiptera: Aphididae) and *Colletotrichum acutatum* (Glomerellales: Glomerellaceae) in pepper. Insects. 2021;12(7):1–18.
26. Sun T, Shen Z, Shaukat M, Du C, Ali S. Endophytic isolates of *Cordyceps fumosorosea* to enhance the growth of *Solanum melongena* and reduce the survival of whitefly (*Bemisia tabaci*). Insects. 2020;11(2):8–10.
27. Mantzoukas S, Chondrogiannis C, Grammatikopoulos G. Effects of three endophytic entomopathogens on sweet sorghum and on the larvae of the stalk borer *Sesamia nonagrioides*. Entomol Exp Appl. 2015;154(1):78–87.
28. Lopez DC, Zhu-Salzman K, Ek-Ramos MJ, Sword GA. The entomopathogenic fungal endophytes *Purpureocillium lilacinum* (formerly *Paecilomyces lilacinus*) and Beauveria bassiana negatively affect cotton aphid reproduction under both greenhouse and field conditions. PLoS One. 2014;9(8):1–8.
29. Elena GJ, Beatriz PJ, Alejandro P, Roberto LE. *Metarhizium anisopliae* (Metschnikoff) Sorokin promotes growth and has endophytic activity in tomato plants. Adv Biol Res (Rennes). 2011;5(1):22–7.
30. Rasool S, Vidkjær NH, Hooshmand K, Jensen B, Fomsgaard IS, Meyling N V. Seed inoculations with entomopathogenic fungi affect aphid populations coinciding with modulation of plant secondary metabolite profiles across plant families. New Phytol. 2021;229(3):1715–27.

Formatted: Font: (Default) Times New Roman

Response to Reviewers 1 Comments

(Manuscript Number: PONE-D-22-19772)

The authors are very much thankful to the reviewers for their meticulous review and valuable comments. We have now incorporated all the two reviewers' comments and suggestions to improve the manuscript. Details of our response to each comment, corrections and suggestions are given below along with the track lines in the manuscript in the manuscript that was first/originally submitted.

Abstract

Response 1: there is no any comment made by reviewer

Introduction

Point 1: s

Response 1: "s" has added in the word "system"

Point 2: pests

Response 2: "s" has been removed from the word "pests"

Point 3: have been

Response 3: "were" has been replaced with "have been" as recommended by reviewer

Point 4: populations

Response 4: population has been changed to populations

Point 5: s

Response 5: "s" has added in the word "interaction"

Point 6: has

Response 6: "has" has been changed to "have"

Point 7: the

Response 7: "a" has been replaced with the

Point 8: doesnt make sense!

Response 8: The statement has been modified

Point 9: by

Response 9: corrected as recommended

Point 10: in full

Response 10: *B. tabaci* has been written in full (*Bemisia tabaci*)

Point 11: new to Malaysia or new in what respect?

Response 11: Both the study and isolates is new to Malaysia

Point 12: whitefly

Response 12: The statement has been modified as recommended

Methods

Point 1: how?

Response 1: The statement has been rephrased as "maintained in a greenhouse under25-30°C and 60-80% RH with a photoperiod of 12L:12D h light: dark" and affected in the manuscript at line 89-90

Point 2: X

Response 2: corrected as recommended

Point 3: “ed”

Response 3: corrected as recommended

Point 4: previously

Response 4: corrected as recommended

Point 5: steel

Response 5: corrected as recommended

Point 6: p

Response 6: corrected as recommended

Point 7: for

Response 7: “in the” has been replaced with for

Point: 8: Not a seedling at 60 days old!

Response 8: Seedlings at 60 days after inoculation (DAI)

Point 9: s

Response 9: "s" has been added after root as recommended

Point 10: s

Response 10: "s" has been added after part as recommended

Point 11: s

Response 11: "s" has been added after root as recommended

Point 12: eggs transfered to control seedlings????

Response 12: The statement has been rephrased

Point 13: quantifying the number of

Response 13: corrected as recommended

Results

Point 1: methods, not really a result but a methodology check!

Response 1: The statement has been removed from the result parts as recommended by reviewer.

Point 2: as assessed

Response 2: Corrected as recommended

Point 3: showed

Response 3: Corrected as recommended

Point 3: for

Response 3: Corrected as recommended

Point 4: what are spores doing inside a host?

Response 4: The statement has been modified as can be seen in the manuscript at line 221-222

Point 5: for

Response 5: on has been replaced with "for"

Point 6: inoculated the seeds not the seedlings!

Response 6: corrected as recommended

Point 7: confusing!

Response 7: The statement has been rephrased as can be seen in the manuscript at line 234-236

Point 8: of

Response 8: in has been replaced with “of”

Point 9: s

Response 9: corrected as recommended

Point 10: not good construct

Response 10: the statements has been reconstructed as can be seen in the manuscript at line 177-180

Point 11: s

Response 11: "s" has been added after shoot

Point 12: which fed

Response 12: corrected as recommended

Point 13: ed

Response 13: "ed" has been added after differ

Point 14: from

Response 14: "with" has been replaced with "from"

Point 15: caused a

Response 15: corrected as recommended

Point 16: s

Response 16: "s" has been added after adult

Point 17: ing

Response 17: corrected as recommended

Discussion

Point 1: d

Response 1: corrected as recommended

Point 2: reduced survuval of B. tabaci adults which has fed on colonized tomato plants

Response 2: corrected as recommended

Point 3: increased

Response 3: corrected as recommended

Point 4: in the number

Response 4: corrected as recommended

Point 5: reword as in first part of discussion.

Response 5: the statement has been reworded as recommended and affected in the manuscript as can be seen at line 296-297

Point 6: poor

Response 6: the statement has been modified

Point 7: Dead

Response 7: corrected as recommended

Conclusion

Point 1: First time this has been done in Malaysia, so worth publishing, even if done in Thailand?

Response 1: Yes. To our knowledge, this is the first report in my Malaysia and some neighboring countries including Thailand

Point 2: was

Response 2: have been has been replaced with "was"

Point 3: following

Response 3: corrected as recommended

Point 4: increased

Response 4: "improved has been replaced with increased

Point 5: survival/killed etc

Response 5: corrected as recommended

Supporting Information

Response 1: there is no any comment made by reviewer

Figures

Point 1: plants

Response 1: corrected as recommended

Point 2: 60 days!!!!!!!!!!!!!!!!!!!

Response 2: corrected as recommended

Point 3: 60 leaves per plant seems a lot?

Response 3: No, we mean leaflet (small portion of leaf) per plants not entire single leaf

Point 1: plants

Response 3: corrected as recommended

Point 1: leaf

Response 3: corrected as recommended

Point 1: Not part of Fig. 1

Response 3: The statement has been removed as it was part of the supporting statement

Point 1: height isnt better?

Response 3: corrected as recommended

Point 1: Never seen a tomato seedling with 80 leaves!!

Response 3: Leaves number has been corrected as leaflets number, because the mean number of leaflets of each seedling was recorded.

The statement has been changed to "number of leaves (leaflets)”

Point 1: mean numbers of leaves has got to be wrong! Impossible to have 80 leaves. Seems to be mixed up with % of something?

Response 3: Leaves number has been corrected as leaflets number, because the mean number of leaflets of each seedling was recorded.

The statement has been changed to "number of leaves (leaflets)”

Point 1: length from root tip to stem tip??

Response 3: all the shoot part of the plant

Point 1: treated

Response 3: corrected as recommended

Point 1: 2

Response 3: corrected as recommended

Point 1: 2

Response 3: corrected as recommended
