# Supplementary material for: Inoculation and colonization of the entomopathogenic fungi, Isaria javanica and Purpureocillium lilacinum, in tomato plants, and their effect on seedling growth, mortality and adult emergence of Bemisia tabaci (Gennadius)
Source: PLoS One. 2023 May 22;18(5):e0285666. doi: 10.1371/journal.pone.0285666 (PMC10202273; doi:10.1371/journal.pone.0285666)
Supplement: Attachment — Submitted filename: Reviewers General comments.docx [file pone.0285666.s008.docx]

**Reviewer #1: General comments**

**Point 1:** The paper is not acceptable in present form. Title: Inoculation and Colonization of Entomopathogenic Fungi, 2 Cordyceps javanica and Purpureocillium lilacinum in 3 Tomato Plants, and their effect on Seedlings Growth, 4 Population, and adult Emergence of Whitefly, Bemisia 5 tabaci (Gennadius)

**Response 1:** The authors are very much thankful to the reviewers for their meticulous review and valuable comments. We have now incorporated all the two reviewers’ comments and suggestions to improve the manuscript.

**Point 2:** The authors should give full names of Cordyceps javanica e.g. should be written as when it appears for the first time and in title e.g. Cordyceps javanica (wf GA17)
Purpureocillium lilacinum should be written as Purpureocillium lilacinum (Thom) Luangsa-ard, Hou- braken, Hywel-Jones & Samson (2011) when it appears first time and in the title.
There is no problem about control treatments used in the experiment.
Thereafter, the two names could be written as C. javanica and P. lilacinum in the annotated text of the paper. There is need to give more information.

**Response 2:** The full name of *Isaria javanica* and *Purpureocillium* *lilacinum* “(*Isaria javanica* (Frieder. & Bally) Samson & Hywel-jone 2005 *and* *Purpureocillium lilacinum* (Thom) Luangsa-ard, Hou- braken, Hywel-Jones & Samson (2011)”) has been written first in the abstract as recommended by reviewer. However, writing two species in full in the title will make it too long which could make readers difficult to digest, inducing the reader with little time and commitment to move on to the next article in his or search.

**Point 3:** The topic is very interesting. Therefore present abstract should be rewritten to give more details about the experiment.

**Response 3:** the abstract has been modified as recommended by reviewer
**Point 4:** The authors should give more information about the induced conclusions based on the experimental results.

**Response 4:** Corrected as recommended
**Point 5:** Keywords: All words shown in the title must not be shown in the keywords. İs very long. It should be shortened

**Response 5:** Corrected as recommended
**Point 6:** authors should not use the language of first or second person. All paper should be written in the language of third person.

**Response 6:** Corrected as recommended

**Reviewer #2: General comments**

**Point 1:** This is an interesting study of the endophytic colonization of tomato plants with two of the less studied species of entomopathogenic fungi. Fungal inoculation was performed by treating the seeds with conidia suspensions. The subsequently inoculated seeds were allowed to germinate and various aspects of the plants were then studies: colonization of different parts of the plants from day 15 to day 60 post inoculation; plant development: seedling height/length, number of leaves, fresh and dry weight of roots, shoots and leaves. Furthermore, the authors also looked at some aspects of the interaction of EFP colonized plants with an important tomato pest, *B. tabaci*. In that case the authors looked at mortality of *B. tabaci* adults placed on the plants and an adult emergence test, which I could not understand at all.

**Response 1:** The authors are very much thankful to the reviewers for their meticulous review and valuable comments. We have now incorporated all the two reviewers’ comments and suggestions to improve the manuscript.

**Point 2:** The results showed the highly positive effects of both fungal isolates on plant development (which has been shown many times before for other EPF endophytes), however, the increased leaf number data is really odd.

**Response 2:** endophytic fungal isolates (Cjc-03 and TS-01) enhanced the growth of inoculated tomato seedlings, as there were significant differences between fungal treated seedlings and the untreated control seedlings with respect to the average height of seedlings and number of leaves at different weeks post-inoculation.

**Point 3;** The colonization of different parts of the plants was variable, with the highest % in leaves, followed by stems and a low level of colonization in roots. The peak colonization was seen at 30 days, falling rapidly at 60 days for leaves. The pattern for stem colonization was different to that of leaves, with the highest levels at 15 days, dropping off at 30 days.
Importantly, the authors used confocal microscopy to look at plant colonization. However, they give little attention to these results and put the images in the supporting material. In fact, the data for microscopy needs explaining as it is rather confusing, but maybe it is worth exploring in more detail as very few endophyte studies have used this technique.

**Response 3:** the data of using confocal microscopy for Endophytic colonization of both isolates (Cjc-03 and TS-01) in tomato plants has been included in the manuscript and effected at line 226-229
**Point 4:** The data for insect-plant interactions is also very confusing, with very little explanation of the M&M or results. I have made some corrections to the PDF file, but the English needs a general overhaul.

**Response 4:** We have incorporated all the comments and suggestions to improve the manuscript.

**Point 5:** Modify the title as very confusing!

**Response 5:** the title has been modified

**Reviewer #3: General comments**

**Point 1:** The hypothesis of the work is wonderful. Eventhough, some minor corrections/typos needs to be corrected. However, it is not clear that how the author has conducted the mortality experiments? How the experiment was conducted? the the adults were relased and observed? Simultaneously how the emergence observation were recorded. This methodology part needs to be described well so as to further understand the esults and conclusion.

**Response 1:** The authors are very much thankful to the reviewers for their meticulous review and valuable comments. We have now incorporated all the two reviewers’ comments and suggestions to improve the manuscript.
