# Supplementary material for: Inoculation and colonization of the entomopathogenic fungi, Isaria javanica and Purpureocillium lilacinum, in tomato plants, and their effect on seedling growth, mortality and adult emergence of Bemisia tabaci (Gennadius)
Source: PLoS One. 2023 May 22;18(5):e0285666. doi: 10.1371/journal.pone.0285666 (PMC10202273; doi:10.1371/journal.pone.0285666)
Supplement: Attachment — Submitted filename: Response to Reviewers 1 extra Comments.docx [file pone.0285666.s011.docx]

**Response to Reviewers 1 Comments**

(Manuscript Number: PONE-D-22-19772)

The authors are very much thankful to the reviewers for their meticulous review and valuable comments. We have now incorporated all the two reviewers’ comments and suggestions to improve the manuscript. Details of our response to each comment, corrections and suggestions are given below along with the track lines in the manuscript in the manuscript that was first/originally submitted.

**Abstract**

**Response 1:** there is no any comment made by reviewer

**Introduction**

**Point 1:** s

**Response 1:** "s" has added in the word "system"

**Point 2:** pests

**Response 2**: "s" has been removed from the word "pests"

**Point 3:** have been

**Response 3:** “were” has been replaced with “have been” as recommended by reviewer

**Point 4:** populations

**Response 4:** population has been changed to populations

**Point 5:** s

**Response 5:** "s" has added in the word "interaction"

**Point 6:** has

**Response 6:** "has" has been changed to "have"

**Point 7:** the

**Response 7:** "a" has been replaced with the

**Point 8:** doesnt make sense!

**Response 8:** The statement has been modified

**Point 9:** by

**Response 9:** corrected as recommended

**Point 10:** in full

**Response 10:** *B. tabaci* has been written in full (*Bemisia tabaci*)

**Point 11:** new to Malaysia or new in what respect?

**Response 11**: Both the study and isolates is new to Malaysia

**Point 12:** whitefly

**Response 12**: The statement has been modified as recommended

**Methods**

**Point 1:** how?

**Response 1:** The statement has been rephrased as "maintained in a greenhouse under25-30°C and 60-80% RH with a photoperiod of 12L:12D h light: dark" and affected in the manuscript at line 89-90

**Point 2:** X

**Response 2:** corrected as recommended

**Point 3:** “ed”

Response 3: corrected as recommended

**Point 4:** previously

Response 4: corrected as recommended

**Point 5:** steel

Response 5: corrected as recommended

**Point 6:** p

Response 6: corrected as recommended

**Point 7:** for

**Response 7:** “in the” has been replaced with for

**Point: 8:** Not a seedling at 60 days old!

**Response 8:** Seedlings at 60 days after inoculation (DAI)

**Point 9:** s

**Response 9:** "s" has been added after root as recommended

**Point 10: s**

**Response 10:** "s" has been added after part as recommended

**Point 11:** s

**Response 11:** "s" has been added after root as recommended

**Point 12:** eggs transfered to control seedlings????

**Response 12:** The statement has been rephrased

**Point 13:** quantifying the number of

**Response 13:** corrected as recommended

**Results**

**Point 1:** methods, not really a result but a methodology check!

**Response 1:** The statement has been removed from the result parts as recommended by reviewer.

**Point 2:** as assessed

**Response 2:** Corrected as recommended

**Point 3:** showed

**Response 3:** Corrected as recommended

**Point 3:** for

**Response 3:** Corrected as recommended

**Point 4:** what are spores doing inside a host?

**Response 4:** The statement has been modified as can be seen in the manuscript at line 221-222

**Point 5:** for

**Response 5:** on has been replaced with "for"

**Point 6:** inoculated the seeds not the seedlings!

**Response 6:** corrected as recommended

**Point 7:** confusing!

**Response 7:** The statement has been rephrased as can be seen in the manuscript at line 234-236

**Point 8:** of

**Response 8:** in has been replaced with “of”

**Point 9:** s

**Response 9:** corrected as recommended

**Point 10:** not good construct

**Response 10:** the statements has been reconstructed as can be seen in the manuscript at line 177-180

**Point 11: s**

**Response 11:** "s" has been added after shoot

**Point 12:** which fed

**Response 12:** corrected as recommended

**Point 13:** ed

**Response 13:** "ed" has been added after differ

**Point 14:** from

**Response 14:** "with" has been replaced with "from"

**Point 15:** caused a

**Response 15:** corrected as recommended

**Point 16:** s

**Response 16:** "s" has been added after adult

**Point 17:** ing

**Response 17:** corrected as recommended

**Discussion**

**Point 1: d**

**Response 1:** corrected as recommended

**Point 2:** reduced survuval of B. tabaci adults which has fed on colonized tomato plants

**Response 2:** corrected as recommended

**Point 3:** increased

**Response 3:** corrected as recommended

**Point 4:** in the number

**Response 4:** corrected as recommended

**Point 5:** reword as in first part of discussion.

**Response 5:** the statement has been reworded as recommended and affected in the manuscript as can be seen at line 296-297

**Point 6:** poor

**Response 6:** the statement has been modified

**Point 7: Dead**

**Response 7:** corrected as recommended

**Conclusion**

**Point 1:** First time this has been done in Malaysia, so worth publishing, even if done in Thailand?

**Response 1:** Yes. To our knowledge, this is the first report in my Malaysia and some neighboring countries including Thailand

**Point 2: was**

**Response 2:** have been has been replaced with "was"

**Point 3:** following

**Response 3:** corrected as recommended

**Point 4:** increased

**Response 4:** "improved has been replaced with increased

**Point 5:** survival/killed etc

**Response 5:** corrected as recommended

**Supporting Information**

**Response 1:** there is no any comment made by reviewer

**Figures**

**Point 1:** plants

**Response 1:** corrected as recommended

**Point 2:** 60 days!!!!!!!!!!!!!!!!!!!

**Response 2:** corrected as recommended

**Point 3:** 60 leaves per plant seems a lot?

**Response 3:** No, we mean leaflet (small portion of leaf) per plants not entire single leaf

**Point 1:** plants

**Response 3:** corrected as recommended

**Point 1: leaf**

**Response 3:** corrected as recommended

**Point 1:** Not part of Fig. 1

**Response 3:** The statement has been removed as it was part of the supporting statement

Point 1: height isnt better?

**Response 3:** corrected as recommended

**Point 1:** Never seen a tomato seedling with 80 leaves!!

**Response 3:** Leaves number has been corrected as leaflets number, because the mean number of leaflets of each seedling was recorded.

The statement has been changed to "number of leaves (leaflets)”

**Point 1:** mean numbers of leaves has got to be wrong! Impossible to have 80 leaves. Seems to be mixed up with % of something?

**Response 3:** Leaves number has been corrected as leaflets number, because the mean number of leaflets of each seedling was recorded.

The statement has been changed to "number of leaves (leaflets)”

**Point 1:** length from root tip to stem tip??

**Response 3:** all the shoot part of the plant

**Point 1:** treated

**Response 3:** corrected as recommended

**Point 1:** 2

**Response 3:** corrected as recommended

**Point 1:** 2

**Response 3:** corrected as recommended
