# Supplementary material for: Inoculation and colonization of the entomopathogenic fungi, Isaria javanica and Purpureocillium lilacinum, in tomato plants, and their effect on seedling growth, mortality and adult emergence of Bemisia tabaci (Gennadius)
Source: PLoS One. 2023 May 22;18(5):e0285666. doi: 10.1371/journal.pone.0285666 (PMC10202273; doi:10.1371/journal.pone.0285666)
Supplement: Attachment — Submitted filename: Response to Reviewers.docx [file pone.0285666.s013.docx]

**Response to Reviewers Comments**

(**Manuscript Number: PONE-D-22-19772**)

The authors are very much thankful to the reviewers for their meticulous review and valuable comments. We have now incorporated all the two reviewers’ comments and suggestions to improve the manuscript. Details of our response to each comment, corrections and suggestions are given below along with the track lines in the manuscript in the manuscript that was first/originally submitted.

**Abstract**

**Point 1:** which affect

**Response 1:** "present in the" has been deleted and replaced with “which affect”

**Point 2:** entomopathogenic fungi

**Response 2**: entomopathogenic fungi has been placed between the words “two and fungal"

**Point 3:** ed

**Response 3:** examining has been changed to "examined"

**Point 4:** enabled

**Response 4:** the word allowed has been deleted and replaced with enabled

**Point 5:** significant

**Response 5:** significant has been placed between the words “caused and mortality"

**Point 6:** A

**Response 6:** small letter **“**a” has been capitalized

**Point 7:** %????

**Response 7:** percentage (%)

**Point 8:** rates

**Response 8:** mean number has been removed and replaced with rate

**Point 9:** were

**Response 9:** was has been changed to were

**Point 10:** use against

**Response 10:** on has been replaced with for use against

**Point 11:** also be

**Response 11:** "also be" has been placed between "could and explored"

**Introduction**

**Point 1:** programs

**Response 1:** system has been replaced with programs

**Point 2:** normally

**Response 2:** usually has been replaced with normally

**Point 3:** insect

**Response 3:** “insect” has been placed after sap-sucking

**Point 4:** ve

**Response 4:** "has" has been changed to have

**Point 5:** s

**Response 5:** "s" has been added in the word plant

**Point 6:** A

**Response 6:** Corrected as recommended

**Point 7:** F

**Response 7:** Corrected as recommended

**Point 8:** tissues

**Response 8:** parts has been replaced with tissues

**Point 9:** y introduced

**Response 9:** The statement has been modified and effected in the manuscript at line 80-81

**Point 10:** recently isolated

**Response 10:** corrected as recommended and affected in the manuscript at line 84

**Point 11:** that could become

**Response 11:** corrected as recommended and affected in the manuscript at line 85

**Materials and methods**

**Point 1:** s

**Response 1:** "s" has been added in the word population

**Point 2:** was

**Response 2:** were has been changed to was

**Point 3:** s

**Response 3:** “s” has been added placed after condition

**Point 4: .**

**Response 4:** full stop has been placed after S1

**Point 5: s**

**Response 5:** “s” has been added placed after suspension

**Point 6: s**

**Response 6:** “s” has been added placed after culture

**Point 7:** strange germ. homogenous might be better

**Response 7:** corrected as recommended

**Point 8: C**

**Response 8:** corrected as recommended

**Point 9: l**

**Response 9:** conidia has been changed to conidial as directed

**Point 10:** to

**Response 10:** to has been place after on

**Point 11:** using

**Response 11:** the word under has been removed and replaced with “using” as directed by reviewer

**Point 12:** used

**Response 12:** the word calculated has been replaced with used as directed.

**Point 13:** A

**Response 13:** corrected as recommended

**Point 14:** described by

**Response 14:** corrected as recommended

**Point 15:** with

**Response 15:** of has been removed and replaced with “with”

**Point 16: +**

**Response 16:** having has been removed and replaced with the symbol +

**Point 17:** that

**Response 17:** and has been removed and replaced with that

**Point 18:** green

**Response 18:** glasshouse has been changed to greenhouse

**Point 19:** slant usually in tubes not dishes??

**Response 19:** PDA slant has been changed to PDA plate

**Point 20:** i

**Response 20:** on has been changed to in

**Point 21:** was used to

**Response 22:** corrected as recommended

**Point 23:** were

**Response 23:** are has been removed and replaced with were

**Point 24:** the effects

**Response 24:** corrected as recommended and affected in the manuscript at line 165

**Point 25: s**

**Response 25:** "s" has been added in the word plant

**Point 26: s**

**Response 26:** "s" has been added in the word plant

**Point 27:** were

**Response 27:** was has been changed to were

**Point 28:** measured

**Response 28:** weighted has been removed and replaced with measured

**Point 29: s**

**Response 29:** "s" has been added in the word seed

**Point 30:** have

**Response 30:** retain has been removed and replaced with the word “have”

**Point 31:** that

**Response 32:** "that" has been placed after adults

**Results**

**Point 1: s**

**Response 1:** corrected as recommended

**Point 2:** however

**Response 2:** and has been removed and replaced with however

**Point 3: of**

**Response 3:** on has been removed and replaced with of

**Point 4:** period

**Response 4:** period has been placed after assessment

**Point 5: s**

**Response 5:** corrected as recommended

**Point 6:** germinating

**Response 6:** germination has been changed to germinating

**Point 7:** in

**Response 7:** on has been changed to in

**Point 8:** were

**Response 8:** were has been placed after formation

**Point 9:** indicating

**Response 9:** indicated has been changed to indicating

**Point 10:** in

**Response 10:** on has been changed to in

**Point 11:** the

**Response 12:** the has been place after effect of

**Point 13:** s

**Response 13:** corrected as recommended

**Point 14: s**

**Response 14:** "s" has been added in the word shoots

**Point 15: s**

**Response 15:** "s" has been added in the word roots

**Point 16:** ly

**Response 16:** significant has been changed to significantly

**Point 17:** had

**Response 17:** corrected as recommended

**Point 18:** told you to stop using the term population. You are looking at a group of experimental insects, not a population in the field!!!

**Response 18:** population has been removed as recommended

**Point 19:** these results were

**Response 19:** corrected as recommended

**Point 20:** a

**Response 20:** corrected as recommended

**Point 21:** the

**Response 21:** the has been placed after “in”

**Point 22:** er

**Response 22:** the word high has been changed to higher

**Point 23:** %??? or number?

**Response 23:** mean number of adult emerged

**Discussion**

**Point 1:** the

**Response 1:** the has been placed after “that”

**Point 2**: tested here

**Response 2:** corrected as recommended

**Point 3:** inoculated

**Response 3:** inoculated has been placed after seeds

**Point 4:** survival

**Response 4:** corrected as recommended

**Point 5:** had

**Response 5:** “has” has been changed to had

**Point 6:** by

**Response 6:** of has been deleted and replaced with by

**Point 7:** be nice to know how this happens!!

**Response 7:** noted with thanks

**Point 8: of**

**Response 8:** to has been deleted and replaced with “of”

**Point 9:** reduced

**Response 9:** corrected as recommended

**Point 10:** have been rarely

**Response 10:** corrected as directed

**Point 11: s**

**Response 11:** corrected as recommended

**Point 12:** actually there are 4 proposals (Vega review), but I guess you cant be bothered to discuss them here!

**Response 12:** the statement has been removed as can be seen in the

**Conclusion**

**Point 1:** in Malaysia

**Response 1:** The statement has been modified and effected in the manuscript at line 30-32

**Point 2:** their

**Response 2:** its has been deleted and replaced with their

**Point 3: s**

**Response 3:** corrected as recommended

**Point 4:** whiteflies

**Response 4:** corrected as recommended

**Point 5:** *B. tabaci*

**Response 5:** corrected as recommended

**Supporting information**

**Point 1:** i

**Response 2:** on has been changed to in

**Figures**

**Response 1:** there is no any comment made by reviewer
